# Chemical and transcriptomic analyses of leaf trichomes from *Cistus creticus* subsp. *creticus* reveal the biosynthetic pathways of certain labdane-type diterpenoids and their acetylated forms

**DOI:** 10.1093/jxb/erae098

**Published:** 2024-03-23

**Authors:** Antigoni S Papanikolaou, Dimitra Papaefthimiou, Dragana Matekalo, Christina-Vasiliki Karakousi, Antonios M Makris, Angelos K Kanellis

**Affiliations:** Group of Biotechnology of Pharmaceutical Plants, Laboratory of Pharmacognosy, Department of Pharmaceutical Sciences, Aristotle University of Thessaloniki, 54124 Thessaloniki, Macedonia, Greece; Group of Biotechnology of Pharmaceutical Plants, Laboratory of Pharmacognosy, Department of Pharmaceutical Sciences, Aristotle University of Thessaloniki, 54124 Thessaloniki, Macedonia, Greece; Group of Biotechnology of Pharmaceutical Plants, Laboratory of Pharmacognosy, Department of Pharmaceutical Sciences, Aristotle University of Thessaloniki, 54124 Thessaloniki, Macedonia, Greece; Group of Biotechnology of Pharmaceutical Plants, Laboratory of Pharmacognosy, Department of Pharmaceutical Sciences, Aristotle University of Thessaloniki, 54124 Thessaloniki, Macedonia, Greece; Institute of Applied Biosciences, Centre for Research & Technology, Hellas (CERTH), 57001 Thessaloniki, Macedonia, Greece; Group of Biotechnology of Pharmaceutical Plants, Laboratory of Pharmacognosy, Department of Pharmaceutical Sciences, Aristotle University of Thessaloniki, 54124 Thessaloniki, Macedonia, Greece; The James Hutton Institute, UK

**Keywords:** BAHD acetyltransferase, *C. creticus* subsp. *creticus*, diterpenoid synthases (diTPSs), gene expression, labda-7,13(*E*)-dien-15-ol, labda-7,13(*E*)-dien-15-yl acetate, labda-13(*E*)-ene-8*α*-ol-15-yl acetate, labdane-type diterpenoids, RNA-seq

## Abstract

Labdane-related diterpenoids (LRDs), a subgroup of terpenoids, exhibit structural diversity and significant commercial and pharmacological potential. LRDs share the characteristic decalin–labdanic core structure that derives from the cycloisomerization of geranylgeranyl diphosphate (GGPP). Labdanes derive their name from the oleoresin known as ‘Labdanum’, ‘Ladano’, or ‘Aladano’, used since ancient Greek times. Acetylated labdanes, rarely identified in plants, are associated with enhanced biological activities. Chemical analysis of *Cistus creticus* subsp. *creticus* revealed labda-7,13(*E*)-dien-15-yl acetate and labda-7,13(*E*)-dien-15-ol as major constituents. In addition, novel labdanes such as *cis*-abienol, neoabienol, *ent*-copalol, and one as yet unidentified labdane-type diterpenoid were detected for the first time. These compounds exhibit developmental regulation, with higher accumulation observed in young leaves. Using RNA-sequencing (RNA-seq) analysis of young leaf trichomes, it was possible to identify, clone, and eventually functionally characterize labdane-type diterpenoid synthase (diTPS) genes, encoding proteins responsible for the production of labda-7,13(*E*)-dien-15-yl diphosphate (*endo*-7,13-CPP), labda-7,13(*E*)-dien-15-yl acetate, and labda-13(*E*)-ene-8*α*-ol-15-yl acetate. Moreover, the reconstitution of labda-7,13(*E*)-dien-15-yl acetate and labda-13(*E*)-ene-8*α*-ol-15-yl acetate production in yeast is presented. Finally, the accumulation of LRDs in different plant tissues showed a correlation with the expression profiles of the corresponding genes.

## Teaser

Trichome transcriptomic analysis was utilized to identify and characterize enzymes responsible for the biosynthesis of the labdanes labda-7,13(*Ε* )-dien-15-yl diphosphate, labda-7,13(*Ε* )-dien-15-yl acetate, and labda-13(*Ε* )-ene-8*α* -ol-15-yl acetate in *Cistus creticus* subsp. *creticus.*

## Introduction

*Cistus creticus* subsp*. creticus,* a member of the *Cistaceae* family, is an endemic (sub-)species in the Mediterranean flora that thrives in adverse environmental conditions. The name *Cistus* originates from the Greek word ‘kistos—κίστος’, which loosely translates to ‘basket’ or ‘box’. It is known to produce ‘Ladano’, a sticky, fragrant resinous exudate that fully covers the aerial parts. Historically, ‘Ladano’ has been used in Mediterranean folk medicine (Duke, 2008; Izzo *et al.*, 2021), as incense, and as a perfume fixative (Langenheim, 2003). Moreover, this resin contains an extraordinary number of high-value secondary metabolites, consisting mainly of labdane-related diterpenoids (LRDs) and flavonoids (Papaefthimiou *et al.*, 2014; Aničić *et al*., 2021; Skorić *et al.*, 2022).

LRDs consist of a group of high-value plant natural products (Papaefthimiou *et al.*, 2014), which are characterized by the presence of a bicyclic decalin ring in the labdadienyl/copalyl diphosphate (CPP) intermediate. The core structure is formed by the protonation-initiated bicyclization of geranylgeranyl diphosphate (GGPP) (Peters, 2010). In *C. creticus* subsp. *creticus*, glandular capitate-type trichomes (Falara *et al.*, 2008; Skorić *et al.*, 2022) produce and store these metabolites (Falara *et al.*, 2008; Skorić *et al.*, 2022). This process follows a tissue-specific and developmental-regulated pattern, with young leaves serving as the main production site (Falara *et al.*, 2008, 2010).

LRDs were first isolated, characterized, and named from *Cistus ladaniferus* (Cocker and Halsall, 1956; Cocker *et al.*, 1956). They have been reported to be the dominant fraction among specialized metabolites isolated from populations of *C. creticus* subsp. *creticus* plants grown in various regions, including Greece (Demetzos *et al.*, 1994b, 1995; Falara *et al.*, 2008; Skorić *et al.*, 2012, 2022; Szeremeta *et al.*, 2017; Kostas, 2018; Rauwald *et al.*, 2019; Izzo *et al.*, 2021), Italy (Sicily) (Loizzo *et al.*, 2013), (Sardinia) (Mastino *et al.*, 2017), Croatia (Politeo *et al.*, 2018), Jordan (Abu-Orabi *et al.*, 2020), and Morocco (Lahcen *et al.*, 2020). LRDs found in *C. creticus* exhibit a wide range of biological properties (Papaefthimiou *et al.*, 2014; Zalegh *et al.*, 2021; Skorić *et al.*, 2022), with relevance to both humans (Chinou *et al.*, 1994; Demetzos *et al.*, 1994a, 1995, 1997, 1999, 2001; Dimas *et al.*, 1998, 1999a, b, 2001, 2006; Kalpoutzakis *et al.*, 1998, 2001; Anastasaki *et al.*, 1999; Fokialakis *et al.*, 2006; Skorić *et al.*, 2022) and plants (Bailey *et al.*, 1975; Seo *et al.*, 2012; Morales-Flores *et al.*, 2013).

The terpene synthase (TPS) gene family is responsible for the production of terpenes (Bohlmann *et al.*, 1998; Chen *et al.*, 2011). A number of TPSs function as terpene cyclases (TCs), catalyzing the cyclization of prenyl diphosphate substrates such as geranyl diphosphate (GPP), farnesyl diphosphate (FPP), and GGPP (Lesburg *et al.*, 1998; Trapp and Croteau, 2001). This reaction involves a three-step electrophilic cascade reaction mechanism consisting of initiation, proliferation, and termination (Ueberbacher *et al.*, 2012).

In the initiation step, enzyme-induced multistep carbocation rearrangements set the foundation for terpene cyclization. Proliferation occurs when the intermediate ion attacks π-electrons from an adjacent C=C bond, generating another carbenium ion. This process can be repeated sequentially, depending on the availability of other C=C bonds in the correct orientation within the substrate. Termination can occur via various alternative routes that may involve: (i) deprotonation of the terminal carbocation, leading to C=C bond formation, or (ii) quenching with a nucleophile, resulting in the formation of alcohol or a diphosphate ester moiety (Bohlmann *et al.*, 1998; Christianson, 2008; Ueberbacher *et al.*, 2012). The vast chemical diversity of natural metabolites is a direct result of the alternative initiation and termination reactions facilitated by TPS enzymes.

Class II TPSs (or TCs) initiate the formation of carbocations by protonating the terminal C=C double bond or the corresponding epoxide (e.g. oxidosqualene) (Wendt *et al.*, 2000). The N-terminal highly conserved ‘aspartate-rich’ DxDD motif is located in the interface of γ and β domains (Wendt and Schulz, 1998; Christianson, 2008; Gao *et al.*, 2012; Tholl, 2015; Pemberton *et al.*, 2017). Within this motif, the ‘middle’ aspartate functions as the catalytic acid, serving as a general acid (Wendt *et al.*, 2000; Prisic *et al.*, 2007). Class II activity is a common feature of all land plants, as the production of *ent*-labda-8(17),13-dienyl diphosphate, commonly known as *ent*-copalyl diphosphate (*ent*-CPP), initiates the biosynthetic pathway that leads to the formation of plant phytohormones.

Dephosphorylation of the terpenoid diphosphate moiety usually occurs through a single-step reaction in a TPS-based pathway. Class I TPSs utilize a conserved trinuclear metal cluster to facilitate the molecular recognition of the substrate diphosphate group and initiate catalysis. This process is triggered by the ionization of the isoprenoid diphosphate substrate, leading to the formation of an allylic cation and inorganic pyrophosphate (PPi) (Wendt and Schulz, 1998; Aaron and Christianson, 2010). However, alternative pathways exist (Sun *et al.*, 2016), involving endogenous phosphatases, Nudix hydrolases, or even a combination of both. A subset of the Nudix hydrolase superfamily have been shown to act as monophosphatases by removing the first phosphate group on non-nucleoside diphosphate-bearing terpenic substrates (Magnard *et al.*, 2015; Henry *et al.*, 2018; Liu *et al.*, 2018; Li *et al.*, 2020; Sun *et al.*, 2020; Bergman *et al.*, 2021).

The terpenic scaffold can be further modified by tailoring enzymes such as BAHD acyltransferases (Moghe *et al.*, 2023). This superfamily of plant-specific, cytosolic (Fujiwara *et al.*, 1998), acyl-CoA-dependent, monomeric enzymes introduces acyl groups into oxygen, nitrogen, or thiol nucleophiles, producing acyl ester derivatives. These enzymes participate in the synthesis of polymers and secondary metabolites, including lignin, volatile esters, anthocyanins, phytoalexins, and alkaloids. The identification of BAHD enzymes is based on several factors, including the presence of the HXXXD catalytic motif located in the middle of the protein sequence and the conserved C-terminal DFGWG structural motif. Members of this enzyme family typically have an average molecular mass of 48–55 kDa. They consist of ~445 amino acids, lack a signal peptide sequence, and exhibit low overall sequence identity (25–34%) (D’Auria, 2006). Prediction of the possible function of uncharacterized BAHD enzymes based solely on amino acid sequences is challenging due to the large number of various putative substrates and the low overall sequence identity among members of this family.

In previous studies conducted by our group, the trichome-specific class II diterpenoid cyclase gene responsible for encoding copal-8-ol diphosphate synthase (CcCLS) was identified (Falara *et al.*, 2010). While the biosynthetic route of copal-8-ol diphosphate has been studied, the formation of many LRD compounds in the glandular trichomes of *C. creticus* remains largely unexplored.

This study reports the isolation, cloning, and functional characterization of *CcLDDS1* and *CcLDDS2*, two distinct class II *endo*-7,13-CPP-encoding genes, along with *CcLAT*, a gene encoding a BAHD acetyltransferase capable of acetylating labdane-type diterpenoids such as labda-7,13(*E*)-dien-15-ol and labda-13(*E*)-ene-8*α*,15-diol. The identification of these sequences was achieved through RNA-sequencing (RNA-seq) analysis on trichomes isolated from young leaves. Subsequently, the sequences were cloned and introduced into heterologous expression systems, such as *Escherichia coli*, *Nicotiana benthamiana*, and/or *Saccharomyces cerevisiae.* The LRDs obtained from transformed cultures and/or plants were identified using GC-MS analysis. Moreover, the reconstitution of labda-7,13(*E*)-dien-15-yl acetate and labda-13(*E*)-ene-8*α*-ol-15-yl acetate production in yeast is presented. Additionally, this study reports the presence of labdanes *cis*-abienol, neoabienol, *ent*-copalol, and an as yet unidentified labdane-type diterpenoid in *C. creticus* subsp. *creticus* for the first time. Furthermore, the potential involvement of Nudix hydrolases in the dephosphorylation reaction of 8-OH-CPP and *endo*-7,13-CPP diphosphate esters is discussed. Finally, the accumulation of LRDs in different plant tissues (leaf stages 1–4, stem, flower, blossom, fruit, and root) and the expression profiles [quantitative real-time PCR (qRT-PCR) analysis] of the corresponding genes were examined.

## Materials and methods

### Plant material

Samples of various *C. creticus* subsp. *creticus* tissues, including four stages (S1–S4) of leaf development (S1, 0.5–1 cm; S2, 1–2 cm; S3, 2–3 cm; and S4, 3–4 cm) (Falara *et al.*, 2008, 2010), as well as stem, flower, blossom, fruit, and root, were collected from 2-year-old, healthy, pest-free acclimated and propagated transplants grown to a height of ~60 cm. These transplants originated from Sises Mylopotamos (Rethymno, Crete, Greece) and have been cultivated in the greenhouses of the Floriculture Laboratory (Aristotle University of Thessaloniki Farm, Thermi, Macedonia). Samples were collected from three distinct groups of plants, with each group consisting of 1–2 different plants. Seeds of *N. benthamiana* plants, which stably express the p19 silencing suppressor gene (*N. benthamiana*-p19), used in the infiltration experiments, were generously provided by Professor K. Kalantidis (Biology Department, University of Crete, Heraklion, Greece).

The plants were cultivated under controlled temperature conditions (25/18 °C, day/night, winter; 32/20 °C, day/night, summer) and natural photoperiod. The greenhouse was equipped with climate control systems and a data logger. Tissue samples collected were stored in Falcon tubes, flash-frozen in liquid nitrogen, and kept at –80 °C until further use, including total RNA and *n*-hexane extraction.

### Isolation of trichomes

*Cistus creticus* subsp. *creticus* trichomes were isolated from young leaves of developmental stage S2, following the protocol as described in Falara *et al.* (2008). Subsequently, RNA-seq and cDNA synthesis were performed on the extracted RNA from these trichomes.

### Chemical analysis of plant tissues

**Fig. 1.**
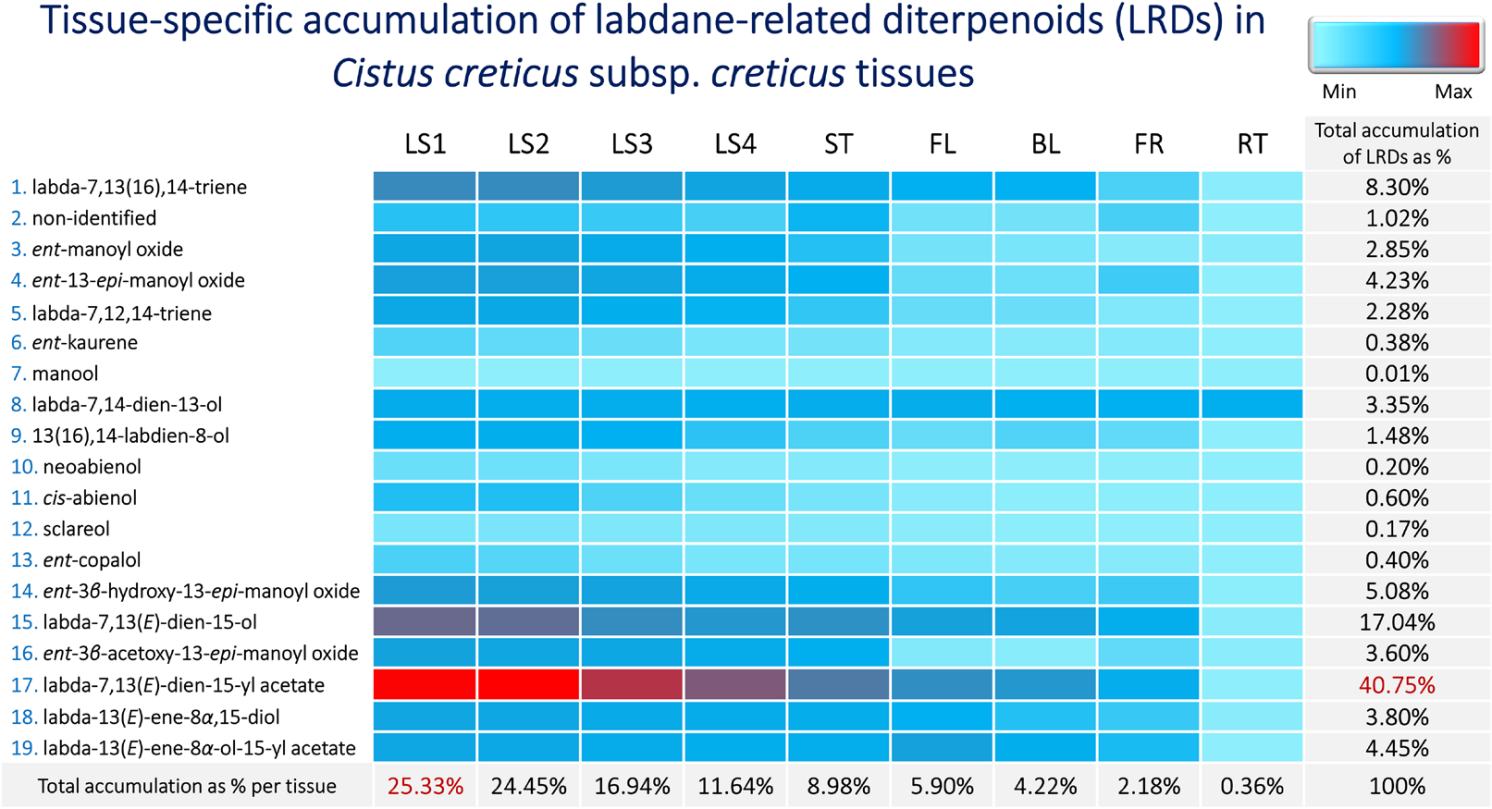
Tissue-specific accumulation of labdane-related diterpenoids (LRDs). Heat map of normalized peak areas of LRDs detected by GC-MS in *C. creticus* subsp. *creticus n*-hexane extracts from different plant tissues: leaves at four developmental stages (LS1-4), stem (ST), flower (FL), blossom (BL), fruit (FR), and root (RT), using nonadecane as the internal control standard (*n*=3, ± SE) (Supplementary Fig. S1; Supplementary Table S1).

**Fig. 2.**
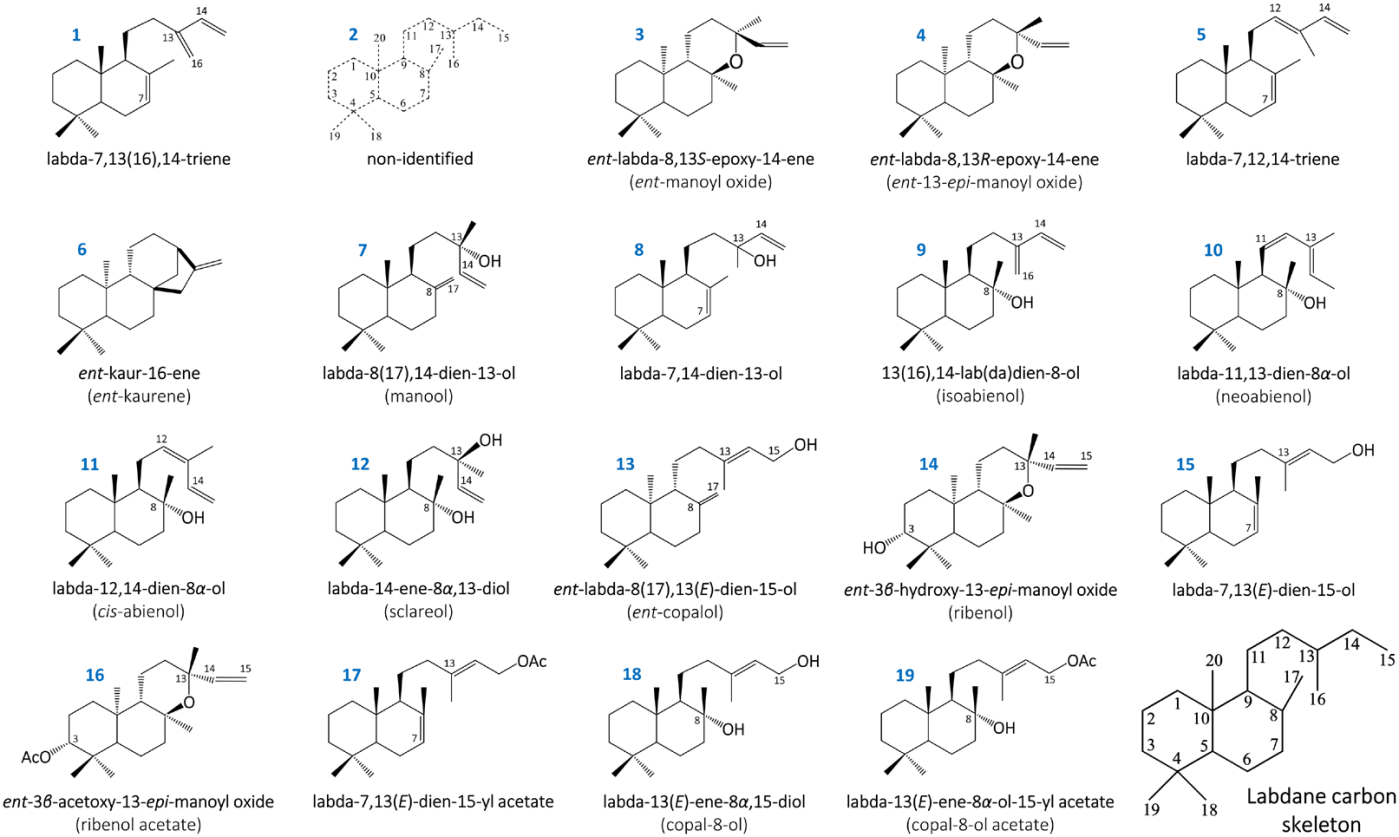
Structures of the 19 LRDs identified by GC-MS in *C. creticus* subsp. *creticus* tissue *n*-hexane extracts. Eighteen compounds are structurally related to labdane-type diterpenoids and one to a tetracyclic hydrocarbon (*ent*-kaurene) (Fig. 1; Supplementary Figs S1, S2; Supplementary Tables S1, S2). The carbon skeleton of labdanes is presented in the lower-right corner.

For the analysis, 200 mg of homogenized tissue, including leaves at stages 1–4, stem, flower, blossom, fruit, and root (Figs 1, 2; Supplementary Figs S1, S2; Supplementary Tables S1, S2), were used. Additionally, 1 mΜ nonadecane (Sigma-Aldrich Co., St. Louis, MO, USA), introduced into the powdered material before the extraction step, was employed as an internal control standard.

The tissues were extracted three times in Erlenmeyer flasks, with each extraction using 20 ml of high-purity *n*-hexane (Sigma-Aldrich Co.). Cells were disrupted by applying a brief incubation period (15 min) with mild agitation (100 rpm) at room temperature, followed by water bath sonication (15 min, room temperature). MgSO_4_, used as a drying agent, was promptly removed using Whatman No.1 filter paper. The resulting 60 ml of extracted tissue samples were evaporated under a stream of air for volume reduction until they could be transferred to a 2 ml screw-cap PTFE septum GC-MS vial.

### RNA extraction and cDNA synthesis

Total RNA extraction from *C. creticus* subsp. *creticus* tissues was performed using the Spectrum Plant Total RNA Kit (Sigma-Aldrich Co.). To eliminate potential genomic DNA contamination, the On-Column DNase I Digest Set (Sigma-Aldrich Co.) was employed. The trichome RNA intended for cDNA synthesis was subjected to an additional purification step using the Rneasy MinElute Cleanup Kit (Qiagen, Valencia, CA, USA). The concentration and quality of the extracted RNA samples were initially assessed using the NanoDrop™ 2000c (Thermo Fisher Scientific Inc., Waltham, MA, USA) and agarose bleach gel electrophoresis with a 2% chlorine solution (Aranda *et al.*, 2012). Finally, the integrity of the RNA samples was confirmed through analysis on the Agilent 2100 bioanalyzer (Agilent Technologies, Inc., Santa Clara, CA, USA).

cDNAs were synthesized from 1 μg of high-quality total RNA using the Superscript III first-strand synthesis kit (Thermo Fisher Scientific Inc.). Random primers (Thermo Fisher Scientific Inc.) were incorporated into the synthesis of cDNA templates used for qRT-PCR analysis. All steps of RNA extraction, purification, and cDNA synthesis were conducted according to the manufacturer’s instructions.

### RNA-seq

Library preparation, transcriptome sequencing, and bioinformatic analysis of trichome RNA were conducted by Beijing Genomics Institute (BGI-Group, Shenzhen, Guangdong, China) using the Illumina HiSEq2000 genome sequencer (Illumina Inc., San Diego, CA, USA). To perform transcriptome sequencing, a minimum concentration of 0.3 μg (15 μl, 20 ng μl^–1^) of high-quality RNA was required to be shipped with dry ice.

Using Trinity (Grabherr *et al.*, 2011), raw reads were analyzed to assemble high-quality contiguous sequences known as contigs. Unigenes were subsequently categorized into two classes through clustering: one cluster consisted of those with the prefix CL (distinct cluster) and another cluster included singletons (distinct singleton) with the prefix unigenes. The functional classification of unigenes against the Clusters of Orthologous Group (COG) database is presented in Supplementary Fig. S3.

### Identification of contigs sharing high similarity with sequences of interest

Candidate contigs were fished-out utilizing BLAST searches against sequences of: (i) characterized class I, II, and I/II plant diTPSs (tBLAST-n search) (selected from Supplementary Table S3), and (ii) BAHD acyltransferase-encoding genes (D’Auria, 2006), including a partial candidate sequence previously detected in *C. creticus* subsp. *creticus* trichomes EST analysis (Falara *et al.*, 2008) (BLAST-n search).

The tBLAST-n (NCBI local BLAST) analysis, employing the Bioedit package (Hall, 1999), was conducted utilizing contigs identified from RNA-seq, set as -nucleotide database-, against diTPS protein sequences, set as -query sequences-. Additionally, a second BLAST-n search was performed against nucleotide query sequences of plant BAHD alcohol acyltransferase-encoding genes, including a partial sequence of a candidate BAHD alcohol acetyltransferase gene from *C. creticus* subsp. *creticus*. In both instances, contig sequences with a BLAST E-value of 1.0E-10 or better, based on the BLOSUM62 matrix, were retained for further analysis. Candidate contigs retrieved from local tBLAST-n (NCBI local BLAST) were translated into amino acid sequences using ExPASy (Gasteiger *et al.*, 2003).

The translated amino acid sequences of candidate contigs were queried against the BLAST-p NCBI database (http://www.ncbi.nlm.nih.gov/BLAST/) (Altschul *et al.*, 1990) to verify sequence identities against known protein sequences and tentatively classify those lacking the motif sequence in their partial read sequences. The top BLAST hits for each translated sequence were collected, and the presence or absence of a motif sequence is reported (Supplementary Table S4).

The ORF is defined as the part of a reading frame that can be translated into a protein. Contigs of interest were analyzed using the ORFfinder service (https://www.ncbi.nlm.nih.gov/orffinder/). Additional ORF sequences were explored by combining overlapping partial contig sequences identified in this study (Supplementary Table S4) with sequences generated from: (i) an EST analysis of *C. creticus* subsp. *creticus* trichomes (Falara *et al.*, 2008), and (ii) an RNA-seq project on *C. creticus* subsp. *creticus* wounded tissue (BioProject ID: PRJNA1074482).

### Phylogenetic analysis and prediction program

**Fig. 3.**
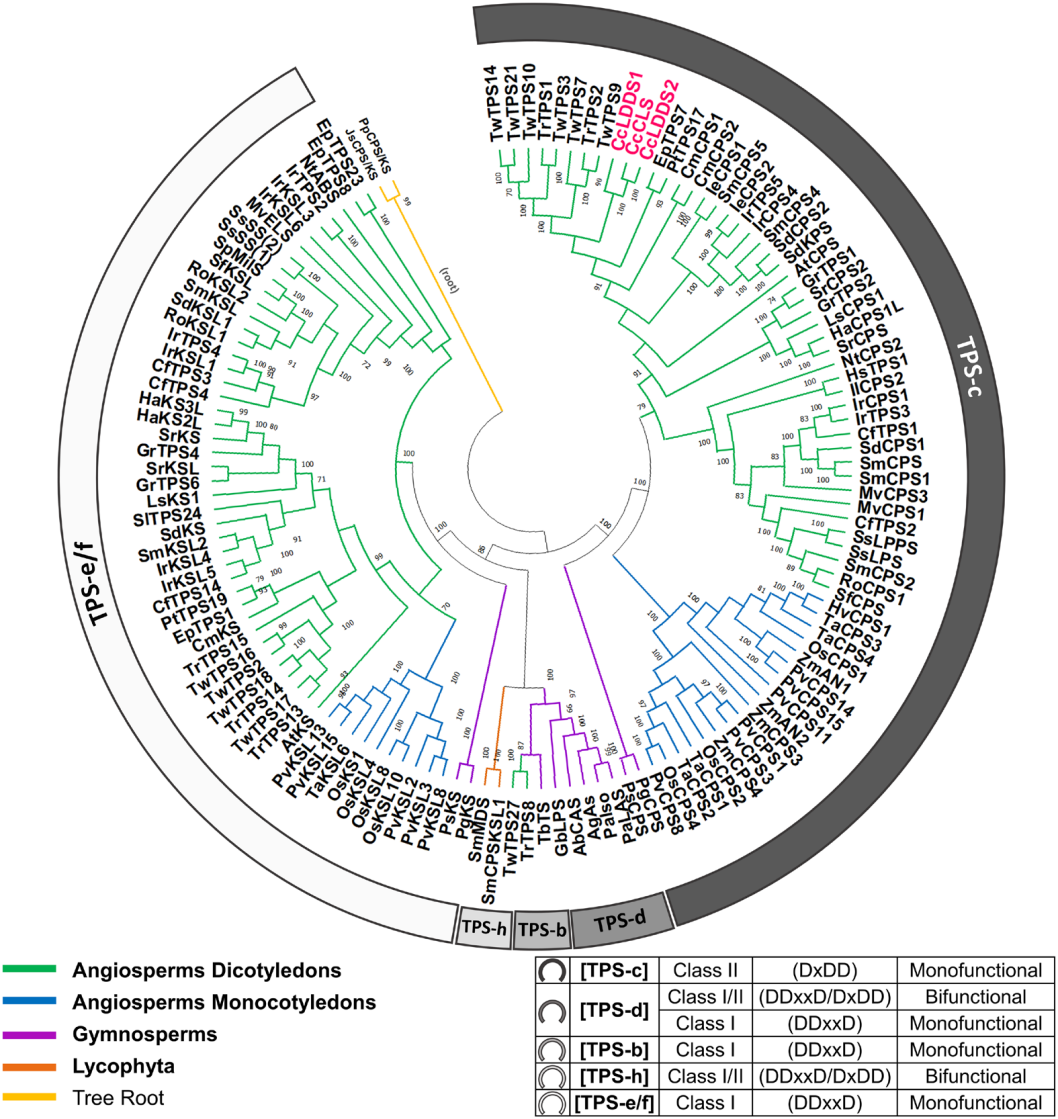
Phylogenetic tree showing evolutionary relationships among characterized plant diterpenoid synthases (diTPSs). Protein sequences of 125 monofunctional and nine bifunctional diTPSs were collected from a total of 42 plant species (Supplementary Table S3). *C. creticus* subsp*. creticus* labda-7,13(*E*)-dien-15-yl diphosphate (*endo*-7,13-CPP) synthases (CcLDDS1 and CcLDDS2) and copal-8-ol diphosphate (8-OH-CPP) synthase (CcCLS) are set in pink font color. Color-coded patterns used in tree branches differentiate among plant groups. Terpenoid synthase subfamilies TPS-c, TPS-d, TPS-b, TPS-h, and TPS-e/f are inscribed along the tree, while information on their enzymatic activity is presented in the table in the lower-right corner. Bootstrap confidence percentages >70% are shown.

Phylogenetic trees were constructed using the Molecular Evolutionary Genetics Analysis (MEGA X) software (Kumar *et al*., 2018). Protein sequences were aligned using the ClustalW algorithm. The optimal diTPSs tree (Fig. 3; Supplementary Table S3) was constructed using the Maximum Likelihood algorithm, employing the Poisson correction model (Zuckerkandl and Pauling, 1965) and 500 bootstrap repetitions (Felsenstein, 1985). The diTPSs tree was rooted using the bryophyte *Physcomitrella patens* (PpCPS/KS, Hayashi *et al.*, 2006) and the liverwort *Jungermannia subulata* (JsCPS/KS, Kawaide *et al.*, 2011).

**Fig. 4.**
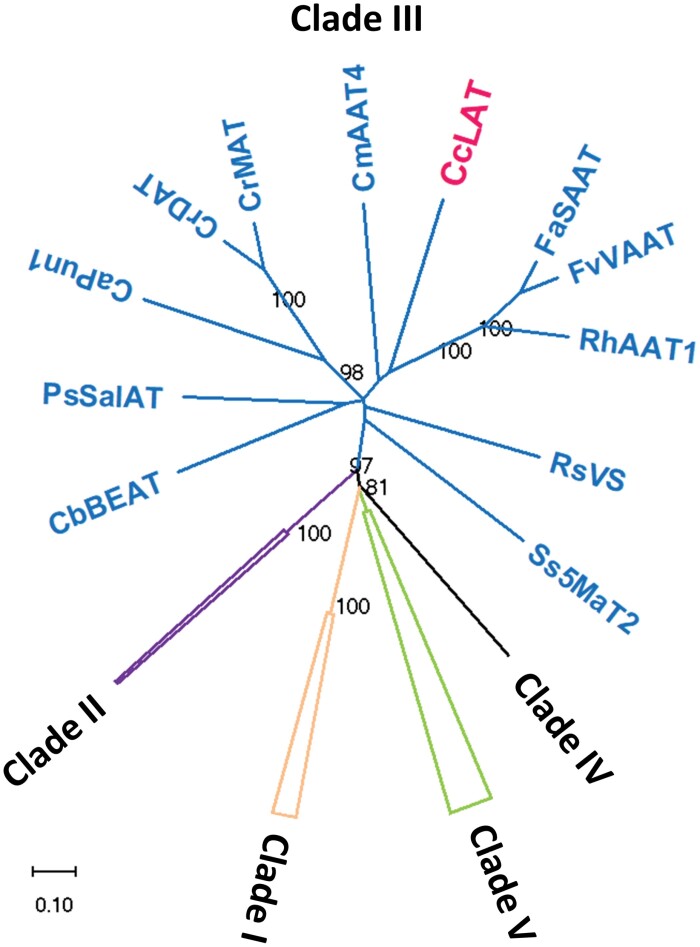
Phylogenetic analysis of BAHD acyltransferases. The tree presented was re-constructed based on the phylogenetic analysis of the superfamily of BAHD acyltransferases (Clade I–V) performed by D’Auria (2006). Bootstrap confidence percentages >70% are shown. *C. creticus* subsp. *creticus* labdane acetyltransferase (CcLAT) is highlighted in pink font color.

The optimal (unrooted) tree depicting phylogenetic relationships among BAHD acyltransferases (Fig. 4; Supplementary Table S5) was constructed using the Neighbor–Joining method based on the Poisson correction model (Zuckerkandl and Pauling, 1965). The tree was drawn to scale, with branch lengths representing the number of amino acid substitutions per site, employing 100 bootstrap repetitions (Felsenstein, 1985). This analysis was based on the phylogenetic analysis of the superfamily of BAHD acyltransferases (Clade I–V) performed by D’Auria (2006).

### Bioinformatics analysis

Protein molecular weight (kDa) and pI for CcLDDS1, CcLDDS2, and CcLAT were calculated using the ExPASy server (Gasteiger *et al.*, 2003). Prediction of putative transit chloroplast peptide was carried out using the SignalP-5.0 and ChloroPV1.1 online prediction servers (Emanuelsson *et al.*, 1999, 2007). Sequence alignment was conducted using the ClustalW algorithm in the Sequence Alignment Editor of the Bioedit Software Program (Hall, 1999), as well as the AlignX program from the Vector NTI software package (Invitrogen-Thermo Fisher Scientific Inc., Waltham, MA, USA).

### Isolation of sequences of interest from *C. creticus* subsp. *creticus* cDNA and cloning into expression vectors

The full-length cDNA of the *CcLDDS1* ORF sequence was obtained through PCR conducted on mRNA extracted from trichomes isolated from young leaves (S2), utilizing KAPA Taq DNA Polymerase (Roche Sequencing Solutions Inc., Pleasanton, CA, USA) and a set of manually designed primers (Supplementary Table S6). Subsequently, TA cloning was performed on the resulting PCR products using both the pGEM®-T Easy Vector (Promega Corporation, Madison, WI, USA) and the pCR™II-TOPO™ Vector (Thermo Fisher Scientific Inc.) cloning systems.

The full-length cDNA of *CcLAT*, as well as cDNAs of *CcLDDS2*(Δ42) lacking the sequence corresponding to the N-plastidial signal peptide, were isolated by PCR performed on mRNA from young leaves (S2). Amplification was carried out using Phusion® Hot Start Flex DNA Polymerase (2 U μl^–1^) (New England Biolabs Inc., Ipswich, MA, USΑ) and/or KAPA HiFi HotStart DNA Polymerase (1 U μl^–1^) (Roche Sequencing Solutions Inc.). Specially designed cloning primers (Supplementary Table S6) were employed to introduce the necessary restriction site overhangs at the 5' and 3' end of the amplified gene sequences (Invitrogen-Thermo Fisher Scientific Inc.).

The vector and amplicon DNA were subjected to double digest using restriction endonucleases (for directional cloning) (New England Biolabs Inc.). The digestion mix, consisting of vector, insert, enzyme, and buffer, was allowed to incubate for ~3.5 h in a 37 °C water bath. Following incubation, a small amount of the linearized vector DNA was compared with an equivalent amount of undigested circular vector DNA through electrophoresis in a 1% agarose gel. Subsequently, T4 DNA ligase (New England Biolabs Inc.) catalyzed the ATP-aided formation of phosphodiester bonds between the sticky ends of the insert and the vector, using ATP as cofactor. Throughout the procedure, purification of PCR products was carried out using the NucleoSpin® Gel and PCR Clean-up Kit (Macherey-Nagel GmbH & Co. KG, Germany) following the manufacturer’s specifications.

Prior to the initiation of gene expression studies, the gene sequences that were isolated and cloned into expression vectors were validated through DNA sequencing performed by CeMIA SA (Larissa, Greece). The obtained results from DNA sequencing were analyzed and visualized using the BioEdit (Ibis Therapeutics, Carlsbad, CA, USA) and the ContigExpress (Thermo Fisher Scientific Inc.) software packages.

### *In vitro* functional characterization of *CcLDDS1*

The mature form of *CcLDDS1* was expressed in a complex with DnaK to enhance solubility in *E. coli.* To that end, the *CcLDDS1*(Δ40) cDNA sequence was successfully transferred from the pGEM-Teasy-*CcLDDS1* construct into the *E. coli* pXCK-K vector (Kyratsous *et al.*, 2009) utilizing specifically designed restriction enzyme overhangs (*Bam*HI/*Not*I, Supplementary Table S6).

Upon sequence confirmation, the pXCK-K*-CcLDDS1*(Δ40) construct was used to transform *E. coli* BL21-CodonPlus(DE3)-RIL competent cells (Agilent Technologies, Inc.). Duplicate bacterial starter cultures were prepared, including: (i) untreated cells (negative control); (ii) cells transformed with the plasmid vector pXCK-K (negative control); and (iii) cells transformed with the pXCK-K*-CcLDDS1*(Δ40) construct.

Prior to induction, bacterial starter cultures were incubated in a shaking incubator (200–250 rpm) set at 37 °C until the exponential phase (OD_600_ ~0.6) was reached. Induction was tested using 0.1 mM and 0.4 mM isopropyl-β-d-thiogalactopyranoside (IPTG). Following induction, cultures were allowed to grow at 24–26 °C for an additional 14 h and 18 h.

Protein profiles of the crude, soluble, and insoluble fractions were analyzed by SDS–PAGE. The 157 kDa 6×His-tagged protein complex DnaK/CcLDDS1(Δ40) was detected in the total (crude) extract at both IPTG concentrations tested during the initial electrophoresis stage. After confirming the successful expression of the protein complex, *E. coli* cells were disrupted using ultrasound sonication treatment (20 s, five repetitions) and the various fractions were separated via centrifugation (13 000 *g*, 20 min, 4 °C). Post-centrifugation, the protein complex was detected in both the soluble (supernatant) and insoluble fractions (cell debris–pellet).

The protein complex was purified using TALON Metal Affinity Resin (Takara Bio Inc., Otsu, Shiga, Japan). Subsequently, pure CcLDDS1(Δ40) was isolated from the purified protein complex through a thrombin enzymatic reaction.

### Expression of *CcLDDS1* in *Nicotiana benthamiana*

The pART7/27 binary vector system (Gleave, 1992) was employed to build a chimeric genetic construct enclosing the complete ORF sequence of *CcLDDS1* (2427 bp). To that end, the cDNA sequence of the *CcLDDS1* gene was PCR-amplified from the pCRII-TOPO-*CcLDDS1* construct and directionally subcloned into the pART7 expression vector (*Kpn*I/*Xba*I, Supplementary Table S6). Upon sequence confirmation, the pART27-*CcLDDS1* construct was utilized to transform the *Agrobacterium tumefaciens* disarmed strain GV3101. Additionally, as a negative control, bacterial cultures transformed with the pART27 vector were also prepared.

To that aim, individual bacterial colonies were cultivated in YEB agar plates containing antibiotics (5 g l^–1^ beef extract, 1 g l^–1^ yeast extract, 5 g l^–1^ peptone, 5 g l^–1^ sucrose, 0.5 g l^–1^ MgCl_2_, and 1.5% agar supplemented with 30 μg ml^–1^ rifampicin, 50 μg ml^–1^ gentamicin, and 50 μg ml^–1^ spectinomycin). Bacterial cells were pelleted by centrifugation (3500 *g*, 15 min, room temperature), resuspended in agroinfiltration medium (Sparkes *et al.*, 2006), and introduced to transgenic *N. benthamiana* plants constitutively expressing the p19 silencing suppressor gene (*N. benthamiana*-p19). The infiltration was performed into the abaxial air spaces of 2- to 4-week-old plant leaves using a syringe without a needle.

To ensure transient expression of the *CcLDDS1* transgene, infiltrated plants were incubated for 4.5 d. Leaf samples collected from each plant were freeze-dried in a container filled with liquid nitrogen. Next, frozen leaves were ground utilizing a mortar and pestle with the addition of small amounts of liquid nitrogen. A 4 g aliquot of pulverized leaves was extracted with 3 × 4 ml of *n*-hexane, following the procedure outlined in Božić *et al.* (2015). Finally, the extracted plant tissue was analyzed by GC-MS.

### *In vivo* functional characterization in *Saccharomyces cerevisiae*

The AM113 yeast strain, developed to increase diterpene production, was employed as a host system for the *in vivo* functional characterization of *CcLDDS1*(Δ40), *CcLDDS2*(Δ42), and *CcLAT* [AM113 genotype: Mat a/alpha, GALp-(K6R)*HMG2*::HOX2, *ura3*, *his3*, *trp1*, P_TDH3_-*HMG2*(K6R)*X*2-::*leu2*, P_TDH3_-*HMG2*(K6R)::HO1, *ERG9/erg9*, *UBC7/ubc7*, *SSM4/ssm4*, *MCT1/mct1*, *WHI2/whi2*, P_TDH3_-*CcGGDPS1*-FLO8, P_TDH3_-*SfFDPS1*-FLO1 derived from AM108] (Kampranis and Makris, 2012).

Gene sequences were isolated and cloned into the yeast pxTDH3myc expression vectors, namely pUTDH3myc (P_TDH3_, 2 μ, *URA3*, myc tag), pHTDH3myc (P_TDH3_, 2 μ *HIS3*, myc tag), and pWTDH3myc (P_TDH3_, 2 μ, *TRP1*, myc tag). Both the AM113 yeast strain and pxTDH3myc expression vectors have been successfully employed in the functional characterization of plant diTPSs, as demonstrated in previous studies (Brückner *et al.*, 2014; Božić *et al.*, 2015).

The N-terminally truncated form of *CcLDDS1*(Δ40) was subcloned from PCRII/TOPO-*CcLDDS1* using *Bam*HI/*Not*I artificial restriction sites into pHTDH3myc, pWTDH3myc, and pUTDH3myc expression vectors. The PCR-isolated *CcLDDS2*(Δ42) sequence was introduced into *Bam*HI/*Sph*I-digested pWTDH3myc. Additionally, the PCR-amplified *CcLAT* cDNA, flanked by *Bam*HI/*Not*I adapters, was directionally cloned into pHTDH3myc. Lastly, the pWTDH3myc-*CcCLS* construct has previously been accomplished by Ignea *et al.* (2015).

The gene *SmCPSKSL1* (GenBank ID: JN001323.1), coding for labda-7,13(*E*)-dien-15-ol synthase in *Selaginella moellendorffii* (Mafu *et al.*, 2011), was subcloned from pDEST17-*SmCPSKSL1* into pWTDH3myc using 5'-*Not*I and 3'-*Xho*I cohesive ends. The primer sequences with specially designed restriction sites are presented in Supplementary Table S6.

Yeast transformation was performed using 1 μg of each DNA plasmid, following a slightly modified LiAc-mediated transformation method (Gietz and Schiestl, 2007). Yeast fermentation procedures were conducted according to Brückner *et al.* (2014) and Trikka *et al.* (2015a). Hexane extraction was performed as reported in Božić *et al.* (2015). Decane extraction, adapted from Trikka *et al.* (2015b), was carried out using Erlenmeyer flasks and a shaking incubator.

### GC-MS

GC-MS analysis was conducted on a Shimadzu GCMS-QP2010 Ultra instrument system equipped with an AOC20i-AOC20s autosampler (Shimadzu Corp., Kyoto, Japan). For the analysis, 2 μl of samples were injected onto the column at 230 °C in splitless mode. Helium (99.999%) was used as the carrier gas with a constant flow rate of 1 ml min^–1^ under electron ionization (EI) mode. Compound separation was achieved on a capillary, non-polar DB-5MS column (30 m×0.25 mm internal diameter×0.25 μm film thickness) (Agilent Technologies, Inc.).

**Fig. 5.**
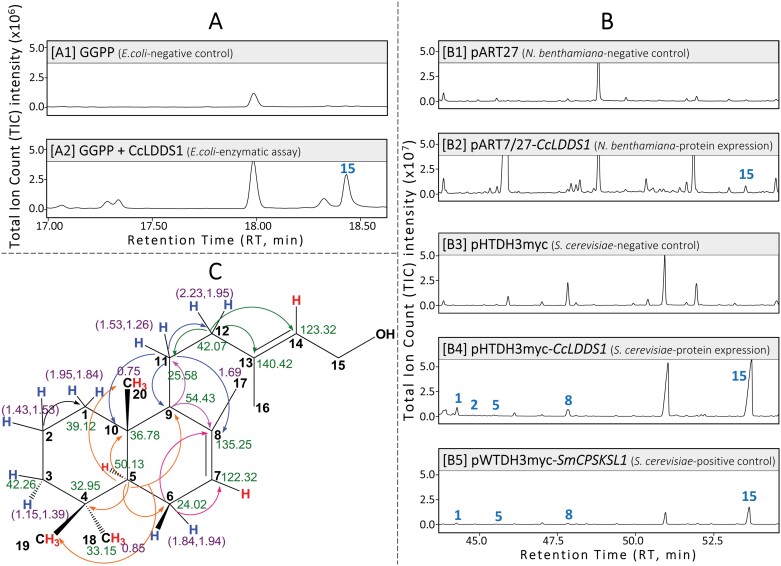
GC-MS analysis of *n*-hexane extracts from the *in vitro* (A) and *in vivo* (B) expression of *C. creticus* subsp*. creticus* labda-7,13(*E*)-dien-15-yl diphosphate (*endo-*7,13-CPP) synthase encoding gene *CcLDDS1*. Total ion current (TIC) chromatograms. (A) 1–2: dephosphorylated enzymatic product(s) of the *in vitro* enzymatic assay. Negative control: dephosphorylated product(s) of geranylgeranyl diphosphate (GGPP). (B) 1–2: transient expression of the *CcLDDS1* gene in *N. benthamiana.* Negative control: product of the pART27 empty vector. (B) 3–5: AM113 yeast strain transformed with the *CcLDDS1*(Δ40) gene. Positive control: AM113 yeast strain transformed with the *SmCPSKSL1* gene [SmCPSKSL1: *Selaginella moellendorffii* bifunctional class I/II labda-7,13(*E*)-dien-15-ol synthase, Mafu *et al.*, 2011]. Negative control: product of the pHTDH3myc empty vector. (C) Structure of peak 15 [labda-7,13(*E*)-dien-15-ol] as verified by 1D (^1^H-NMR, ^13^C-NMR) and 2D (COSY H-H, HSQC, HMBC) NMR analysis. Peaks: 1, labda-7,13(16),14-triene; 2, non-identified compound; 5, labda-7,12,14-triene; 8, labda-7,14-dien-13-ol; and 15, labda-7,13(*E*)-dien-15-ol.

**Fig. 6.**
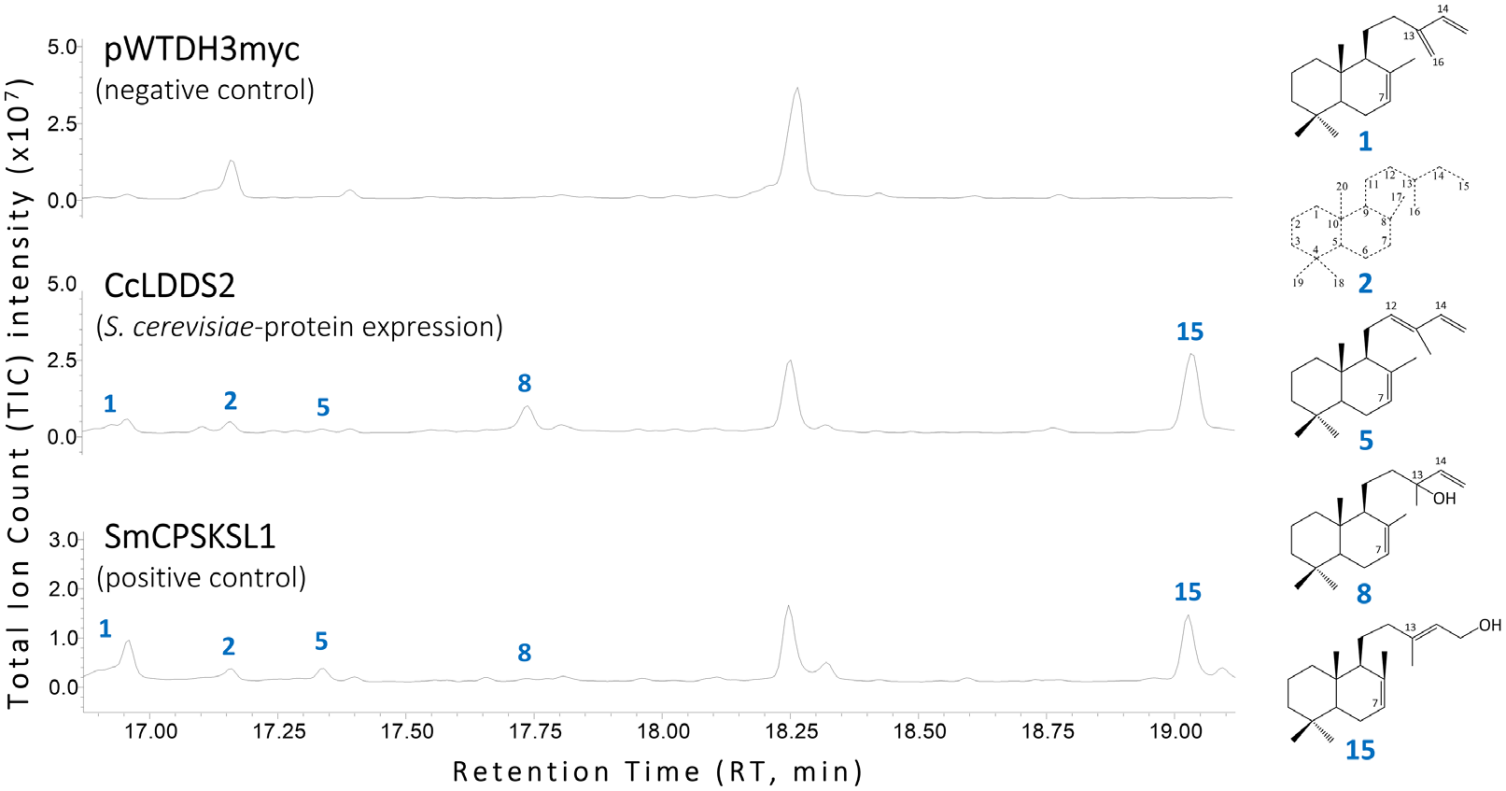
*In vivo* functional characterization of *C. creticus* subsp*. creticus* labda-7,13(*E*)-dien-15-yl diphosphate (*endo*-7,13-CPP) synthase encoding gene *CcLDDS2* using GC-MS. Total ion current (TIC) chromatograms of decane extracts from the *S. cerevisiae* AM113 strain transformed with the *CcLDDS2*(Δ42) gene. Positive control: AM113 yeast strain transformed with the *SmCPSKSL1* gene [SmCPSKSL1: *Selaginella moellendorffii* bifunctional class I/II labda-7,13(*E*)-dien-15-ol synthase] (Mafu *et al.*, 2011). Negative control: the product of the pWTDH3myc empty vector. Peaks: 1, labda-7,13(16),14-triene; 2, non-identified compound; 5, labda-7,12,14-triene; 8, labda-7,14-dien-13-ol; and 15, labda-7,13(*E*)-dien-15-ol.

**Fig. 7.**
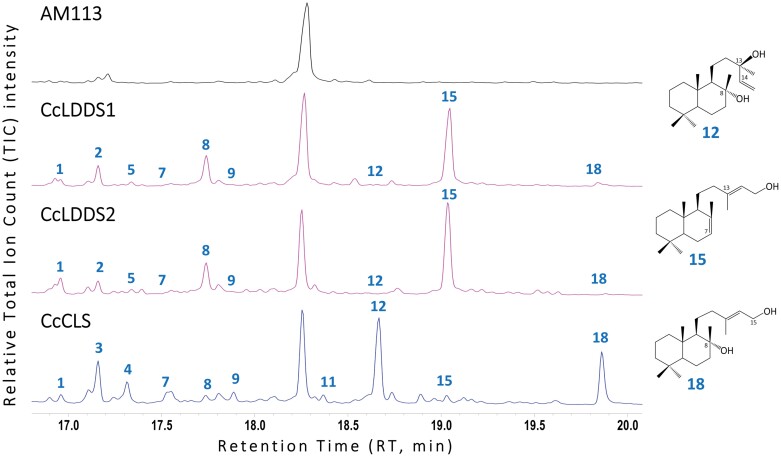
GC-MS analysis of the heterologous expression of CcLDDS1, CcLDDS2, and CcCLS in *S. cerevisiae* AM113 strain. Total ion current (TIC) chromatograms of decane extracts of AM113 yeast strain transformed with the constructs pWTDH3myc-*CcLDDS1*(Δ40), pWTDH3myc-*CcLDDS2*(Δ42), and pWTDH3myc-*CcCLS.* Negative control: AM113 untreated cells. Peaks identified: 1, labda-7,13(16),14-triene; 2, non-identified compound; 3, manoyl oxide, 4, 13-*epi*-manoyl oxide; 5, labda-7,12,14-triene; 7, manool; 8, labda-7,14-dien-13-ol; 9, 13(16),14-labdien-8-ol; 11, *cis*-abienol; 12, sclareol; 15, labda-7,13(*E*)-dien-15-ol; and 18, labda-13(*E*)-ene-8*α*,15-diol.

**Fig. 8.**
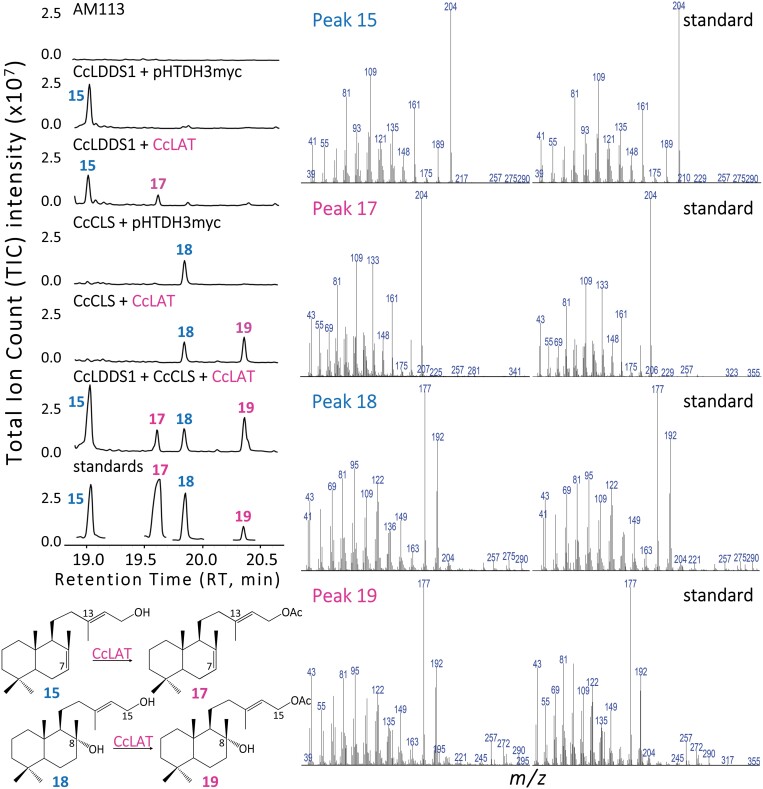
GC-MS analysis of *C. creticus* subsp*. creticus* labdane acetyltransferase (CcLAT) *in vivo* functional characterization using authentic standards. Total ion current (TIC) chromatograms of decane extracts of *S. cerevisiae* AM113 strain transformed with the constructs pUTDH3myc-*CcLDDS1*(Δ40), pWTDH3myc*-CcCLS*, and pHTDH3myc*-CcLAT.* Negative control: AM113 untreated cells and pHTDH3myc empty vector. Mass spectra of the produced peaks: 15, labda-7,13(*E*)-dien-15-ol; 17, labda-7,13(*E*)-dien-15-yl acetate; 18, labda-13(*E*)-ene-8*α*,15-diol; and 19, labda-13-ene-8*α*-ol-15-yl acetate.

A GC thermal cycle of 24 min duration, as described by Morrone *et al.* (2011), was employed to analyze samples from organic extracts of: (i) *in vitro* characterization of the *CcLDDS1* gene product (Fig. 5A); (ii) transformed yeast cultures producing *CcLDDS1* and *CcLDDS2* gene products (Fig. 7); and (iii) *in vivo* characterization of *CcLDDS2* (Fig. 6) and *CcLAT* gene products (Fig. 8).

A second GC thermal cycle of 76 min duration was employed to analyze *n*-hexane extracts of the *in vivo* characterization of the *CcLDDS1* gene product (Fig. 5B). Here, the oven temperature (60 °C) was initially raised by 3 °C min^–1^ up to 240 °C (5 min) and then raised again by 10 °C min^–1^ until reaching 300 °C, where it was maintained for an additional 5 min.

A third GC thermal cycle of 56 min duration was employed to analyze *n*-hexane extracts of *C. creticus* subsp. *creticus* tissues (Fig. 1; Supplementary Fig. S1; Supplementary Table S1). The GC thermal cycle employed consisted of the oven temperature raised from 50 °C (3 min) at a rate of 12 °C min^–1^ up to 160 °C, followed by an increase of 3 °C min^–1^ up to 230 °C, and finally by 20 °C min^–1^ until reaching 325 °C where it remained for 15 min.

The GC-MS chromatograms were analyzed using the GCMSsolutions (Shimadzu Corp., Kyoto, Japan) software package. Comparative analyses of the produced peaks were conducted using: (i) commercial MS libraries, including the Flavour & Fragrance Natural & Synthetic Compounds GCMS library (FFNSC 2, 2012) and the National Institute of Standards & Technology, Wiley (NIST) mass spectral library; (ii) hexane extracts of labda-7,13(*E*)-dien-15-ol, labda-13(*E*)-ene-8*α*,15-diol, labda-7,13(*E*)-dien-15-yl acetate, and labda-13(*E*)-ene-8*α*-ol-15-yl acetate standards (Fig. 8), which were kindly provided to us by Professor C. Demetzos (Department of Pharmacy, National and Kapodistrian University of Athens, Greece). These standards were isolated from *C. creticus* subsp. *creticus* and structurally analyzed using NMR; and (iii) hexane extract of a transformed yeast culture expressing the bifunctional class I/II labda-7,13(*E*)-dien-15-ol synthase from *S. moellendorffii* (SmCPSKSL1, Mafu *et al.*, 2011) (Figs 5, 6). The *SmCPSKSL1* gene sequence was kindly provided to us by Professor R.J. Peters (Department of Biochemistry, Biophysics and Molecular Biology Iowa State University, USA).

Retention index (RI) values were calculated and compared against their reported values in the scientific literature (Pichette *et al.*, 1998; Anastasaki *et al.*, 1999; Demetzos *et al.*, 2002; Skaltsa *et al.*, 2003; Saroglou *et al.*, 2006; Skorić *et al.*, 2012, 2022; Kännaste *et al.*, 2018) and in the GC-MS mass spectral libraries: (i) Flavour & Fragrance Natural & Synthetic Compounds GC-MS library (FFNSC 2, 2012); (ii) Adams (2007); and (iii) massfinder 3 (Hochmuth, 2007) (Supplementary Table S2).

### NMR structural analysis of labda-7,13(*E*)-dien-15-ol

Labda-7,13(*E*)-dien-15-ol was obtained by extracting 5600 ml of the AM113-transformed yeast culture, expressing the pseudo-mature protein product of the *CcLDDS1*(Δ40) gene, using an equal volume of *n*-hexane. This process was repeated three times. The solvent (16 800 ml of *n*-hexane) was then removed under reduced pressure in a rotary evaporator at 65 °C (Büchi, New Castle, DE, USA), resulting in 128 mg of dried extract. The dried extract was subsequently dissolved in *n*-hexane and subjected to column chromatography (90 × 4.5 cm) on silica gel 60/particle size 0.063–0.04 mm (Merck & Co., Inc., Kenilworth, NJ, USA) using solvents of increasing polarity (*n*-hexane→EtOAc→MeOH). Structural identification was performed using ^1^H NMR proton, gCOSY, gHSQC, and gHMBC spectra, which were acquired in CDCl_3_ on Agilent (Varian) DD2, 500 MHz and 125 MHz for ^13^C NMR (Agilent Technologies, Inc.) (Fig. 5C; Supplementary Figs S4–S8; Supplementary Table S7).

### Quantitative real-time PCR

qRT-PCRs were performed on the Applied Biosystems StepOne Real-Time of PCR System (Thermo Fisher Scientific Inc.) using the KAPA SYBR FAST qPCR Kit (Roche Sequencing Solutions Inc.) and manually designed primers producing amplicons of ~100 bp length (Supplementary Table S8). PCR amplicons were verified using DNA sequencing.

The cycling conditions for qRT-PCR were as follows: 3 min/95 °C for one cycle, (15 s/95 °C, 15 s/68 °C, 20 s/72 °C) for 40 cycles, followed by a single melting curve cycle: 15 s/95 °C, 1 min/60 °C, temperature increase 60–95 °C at a rate 1 °C s^–1^, 15 s/95 °C.

The relative transcript abundance was calculated using the equation: 2^-ΔCT (ΔCT=CT_Target gene_–CT_Reference genes_, CT: cycle threshold value). Here the average CT value of *CcActin* (actin) and *CcELFa* (elongation factor a) served as CT reference genes, [CT_RG_=(CT_CcActin_+CT_CcELFa_)/2].

Experiments were conducted on three biological replicates using two technical replicate measurements. Transcript abundance values, measured through qRT-PCR analysis, were expressed in arbitrary units and normalized using *C. creticus* subsp. *creticus* endogenous *CcActin* and *CcELFa* genes. Student’s *t*-test was employed to evaluate the statistical significance of the differences in gene expression among the examined plant tissues and are depicted as *P<0.05, **P<0.01, ***P<0.001 with confidence level equal to 0.95.

## Results

### Tissue-specific accumulation of labdane-related diterpenoids in *C. creticus* subsp. *creticus* tissues

A comprehensive analysis of LRDs was conducted to obtain a better understanding of their tissue-specific accumulation (Figs 1, 2; Supplementary Fig. S1; Supplementary Table S1). A total of 19 LRDs were identified in the plant aerial parts, with primarily accumulation in younger leaves (LS1, 25.33%; LS2, 24.45%) compared with older leaves (LS3, 16.94%; LS4, 11.64%), followed by stems (8.98%), flowers (5.90%), blossoms (4.22%), and fruits (2.18%), while roots had almost undetectable levels. Notably, the as yet unidentified labdane-type diterpenoid and the labda-13(*E*)-ene-8*α*-ol-15-yl acetate were predominantly found in stems and flowers, respectively.

Among the 19 identified LRDs, labda-7,13(*E*)-dien-15-yl acetate was the most abundant compound (40.75%), primarily found in young leaves, followed by labda-7,13(*E*)-dien-15-ol (17.04%) and labda-7,13(16),14-triene (8.30%). The group of *ent*-manoyl oxides, including *ent*-3*β*-hydroxy-13-*epi*-manoyl oxide (5.08%), *ent*-13-*epi*-manoyl oxide (4.23%), *ent*-3*β*-acetoxy-13-*epi*-manoyl oxide (3.60%), and *ent*-manoyl oxide (2.85%) were also identified. Additionally, labda-13(*E*)-ene-8*α*,15-diol (3.80%) and labda-13(*E*)-ene-8*α*-ol-15-yl acetate (4.45%), along with labda-7,12,14-triene (2.28%) and labda-7,14-dien-13-ol (3.35%), were detected at significantly lower levels. Finally, 13(16),14-labdien-8-ol (1.48%), the as yet unidentified labdane (1.02%), *cis*-abienol (0.60%), *ent*-copalol (0.40%), *ent*-kaurene (0.38%), neoabienol (0.20%), sclareol (0.17%), and manool (0.01%), were identified as the least produced LRDs in all examined plant tissues (Figs 1, 2; Supplementary Fig. S1; Supplementary Table S1).

### RNA-seq

Trichomes isolated from S2 leaves were selected for RNA-seq analysis based on the accumulation of LRDs and the expression of *CcCLS* gene in young leaves (Falara *et al.*, 2008, 2010) (BioProject ID: PRJEB71924). The contig and unigene statistics, along with the assembly quality, are summarized in Supplementary Table S9. Sequences of interest were categorized into three main functional classes: (Q) biosynthesis, transport, and catabolism of secondary metabolites, (S) function unknown, and (V) defense mechanisms (Supplementary Fig. S3).

### Bioinformatic sequence analysis and identification of *CcLDDS1*, *CcLDDS2*, and *CcLAT* genes

A total of 43 contigs, sharing strong sequence similarities with known genes coding for class II (DxDD) plant diTPSs, were retrieved by Basic Local Alignment Search Tool (BLAST) analysis (Supplementary Table S4). Among them, contig 68 (2824 nt) contained an ORF sequence of 2427 bp, corresponding to a potential 808 amino acid putative class II diTPS (DTDD) with a mol. wt of 92.52 kDa and isoelectric point (pI) of 5.95. Additionally, a 55 amino acid chloroplast transit peptide in the N-terminal protein domain was identified. The newly discovered diTPS was named CcLDDS1 (*C. creticus* Labdane-type Diterpenoid Diphosphate Synthase 1, GenBank ID: MT666220).

In an effort to enrich the number of full-length contigs, additional BLAST searches were carried out against contigs obtained from RNA-seq analysis of isolated trichomes from S2 wounded leaves of *C. creticus* subsp. *creticus* (BioProject ID: PRJNA1074482). The analysis identified contigs 936 (686 nt) and 541 (1075 nt) as parts of a single ORF sequence (2490 bp) encoding a putative class II diTPS (829 amino acids, DxDD) (Supplementary Fig. S9) with estimated mol. wt 94 kDa and pI of 5.77. Additionally, a 50 amino acid chloroplast transit peptide located in the N-terminal protein domain was identified. This putative diTPS was termed CcLDDS2 (*C. creticus* Labdane-type Diterpenoid Diphosphate Synthase 2, GenBank ID: MT666221).

The amino acid sequence alignment of CcLDDS1, CcLDDS2, and the previously reported CcCLS (Falara *et al.*, 2010) is presented in Supplementary Fig. S10. The sequences of CcLDDS1 and CcLDDS2 shared similarities equal to 72% and 75% with CcCLS, respectively, while sharing only 60% similarity with each other (Supplementary Table S10).

Phylogenetic analysis was performed on the newly identified diTPS sequences comparing them against 132 characterized members of the TPS-b, TPS-c, TPS-d, TPS-e/f, and TPS-h families of plant diTPSs (Fig. 3; Supplementary Table S3). Protein sequences from 42 plant species, consisting of angiosperms, gymnosperms, lycophytes, a liverwort, and a bryophyte were considered in this analysis. CcLDDS1 and CcLDDS2 clustered together with CcCLS in the TPS-c subfamily branch of diTPSs, inferring their function as putative class II diTPSs (DxDD).

Contig sequences obtained from RNA-seq were compared against known BAHD acyltransferase-encoding genes employing BLAST search. A putative alcohol BAHD acetyltransferase-encoding gene sequence was identified from the trichomes EST library of *C. creticus* subsp. *creticus* in a previous study (Falara *et al.*, 2008). BAHD acetyltransferases play an active role in the biosynthetic pathway of various terpenoids (D’Auria, 2006). Here, it was investigated whether this class of enzymes is involved in the acetylation reaction leading to the formation of labda-7,13(*E*)-dien-15-yl acetate and labda-13(*E*)-ene-8*α*-ol-15-yl acetate in *C. creticus* subsp. *creticus*. BLAST search of the partial ESTs against the contigs obtained from RNA-seq retrieved a complete putative gene sequence (contig 149, 1640 nt) and four additional partial sequences (contigs: 6138, 11 027, 3042, and 4520) ranging from 143 nt to 246 nt.

The full-length sequence was subjected to bioinformatic analysis, revealing an ORF of 1347 bp, which encoded a putative acetyltransferase consisting of 448 amino acids, with a mol. wt of 50.59 kDa and pI of 8.42. The newly identified BAHD acetyltransferase was termed CcLAT (*C. creticus* Labdane AcetylTransferase, GenBank ID: MT666224). CcLAT contained the characteristic motifs HXXXD and DFGWG (NFGWG) of the BAHD superfamily of acetyltransferases (Supplementary Fig. S11).

Phylogenetic analysis of the newly identified CcLAT against members of the superfamily of BAHD acyltransferases was performed according to D’Auria (2006) and it was found that CcLAT clustered with *Rosa*×*hybrida* geraniol/citronellol acetyltransferase (RhAAT1, 457 amino acids—GenBank ID: AAW31948, 37% BLAST-p similarity) (Shalit *et al.*, 2003), *Fragaria vesca* alcohol acetyltransferase (FvVAAT, 455 amino acids—GenBank ID: CAC09062, 37% BLAST-p similarity) (Beekwilder *et al.*, 2004), and *Fragaria*×*ananassa* alcohol acetyltransferase (FaSAAT, 452 amino acids—GenBank ID: AAG13130, 36% BLAST-p similarity) (Aharoni *et al.*, 2000) (Fig. 4; Supplementary Table S5). The alignment of CcLAT, RhAAT1, FvVAAT, and FaSAAT protein sequences is presented in Supplementary Fig. S11.

### Functional characterization of the class II labdane-type diterpenoid synthase genes *CcLDDS1* and *CcLDDS2*

Functional characterization of *CcLDDS1* and *CcLDDS2* genes showed that they both encode active members of class II labdane-type diTPSs capable of catalyzing the formation of labda-7,13(*E*)-dien-15-ol, apparently via the formation of *endo*-7,13-CPP using GGPP as a substrate (Figs 5, 6). Furthermore, traces of four additional products, namely labda-7,13(16),14-triene (peak 1), a non-identified labdanic product (peak 2), labda-7,12,14-triene (peak 5), and labda-7,14-dien-13-ol (peak 8), were also detected.

*In vitro* characterization of the *CcLDDS1* gene product involved the overexpression of the construct pXCK-K-*CcLDDS1*(Δ40) in *E. coli*. The CcLDDS1 enzyme, following purification and incubation with GGPP, yielded a diphosphate product. Subsequently, the intermediate diphosphate product, following dephosphorylation with alkaline phosphatase, *n*-hexane extraction, and GC-MS analysis, revealed a peak (peak 15), which was confirmed as labda-7,13(*E*)-dien-15-ol by comparison with commercial MS libraries (Fig. 5A).

*In vivo* characterization was accomplished by expressing the *CcLDDS1* gene in model organisms *N. benthamiana* and *S. cerevisiae* using the constructs pART7/27-*CcLDDS1* and pHTDH3myc-*CcLDDS1*(Δ40), respectively (Fig. 5B). To serve as a positive control for the production of labda-7,13(*E*)-dien-15-ol, a transformed yeast culture was used carrying the *SmCPSKSL1* gene, coding for bifunctional class I/II labda-7,13(*E*)-dien-15-ol synthase in *S. moellendorffii* (Mafu *et al.*, 2011) (Fig. 5B-5). Finally, the structure of the main product (peak 15) was analyzed by NMR and was found to share spectra identical to labda-7,13(*E*)-dien-15-ol (Fig. 5C; Supplementary Figs S4–S8; Supplementary Table S7) (Mafu *et al.*, 2011; Zerbe *et al.*, 2015; Johnson *et al.*, 2019).

Functional characterization of *CcLDDS2* was conducted through the yeast expression system using the pWTDH3myc-*CcLDDS2*(Δ42) construct. As a positive control, a transformed yeast culture expressing the *SmCPSKSL1* gene was utilized (Fig. 6).

### GC-MS comparative analysis of yeast cells expressing *CcLDDS1*, *CcLDDS2*, and *CcCLS* genes

Earlier studies demonstrated that copal-8-ol diphosphate synthase isolated from *Salvia sclarea* (SsLPPS; Caniard *et al.*, 2012) and *Nicotiana glutinosa* (NgCLS; Criswell *et al.*, 2012), using GGPP as a substrate, led to the production of 8-OH-CPP along with the formation, in minor amounts, of the non-hydroxylated (+)-CPP (Caniard *et al.*, 2012) and of the double bond isomer *endo*-7,13-CPP (Criswell *et al.*, 2012). As CcCLS catalyzes the same reaction in *Cistus* leaves yielding 8-OH-CPP (Falara *et al.*, 2010), it was hypothesized that it might also exhibit low catalytic specificity; thus, a similar postulation was put forward for the other two class II diTPS genes, *CcLDDS1* and *CcLDDS2*, characterized in this study. Indeed, when the yeast culture volumes and extraction time of the above genes and *CcCLS* were increased by 2-fold compared with normal experimental conditions, minor peaks were also detected. In detail, a total of 12 peaks were identified by GC-MS namely: (i) labda-7,13(16),14-triene [peak 1, retention time (RT) 16.92 min]; (ii) a non-identified labdane (peak 2, RT 17.16 min); (iii) manoyl oxide (peak 3, RT 17.17 min); (iv) 13-*epi*-manoyl oxide (peak 4, RT 17.3 min); (v) labda-7,12,14-triene (peak 5, RT 17.34 min); (vi) manool (peak 7, RT 17.52 min); (vii) labda-7,14-dien-13-ol (peak 8, RT 17.74 min); (viii) 13(16)-14-labdien-8-ol (peak 9, RT 17.88 min); (ix) *cis*-abienol (peak 11, RT 18.36 min); (x) sclareol (peak 12, RT 18.66 min); (xi) labda-7,13(*E*)-dien-15-ol (peak 15, RT 19.05 min); and (xii) labda-13(*E*)-ene-8*α*,15-diol (peak 18, RT 19.86 min). These labdanes are known metabolites of *endo*-7,13-CPP, 8-OH-CPP, and (+)-CPP produced under enzymatic and non-enzymatic conditions such as the pH conditions during the extraction process (Schalk *et al.*, 2012).

The predominant detectable metabolic products of CcLDDS1 and CcLDDS2 were derivatives of *endo*-7,13-CPP, with labda-7,13(*E*)-dien-15-ol (peak 15) being the primary dephosphorylated alcohol derivative, whereas trace amounts of products originating from 8-OH-CPP and (+)-CPP were also detected. In the case of *CcCLS*, derivatives of the dephosphorylated 8-OH-CPP were the main peaks, including labda-13(*E*)-ene-8*α*,15-diol (peak 18) and sclareol (peak 12) as main metabolites. Additionally, traces of *endo*-7,13-CPP and (+)-CPP intermediate metabolites were also observed (Fig. 7).

### Functional characterization of the BAHD alcohol labdane acetyltransferase CcLAT

The full-length *CcLAT* cDNA was cloned into the pHTDH3myc vector and expressed in the AM113 yeast expression system. Transformed yeast cells carrying the pUTDH3myc-*CcLDDS1* and pWTDH3myc*-CcCLS* constructs, either alone or in combination, produced CcLAT substrates labda-7,13(*E*)-dien-15-ol (peak 15, RT 19.03 min) and labda-13(*E*)-ene-8*α*,15-diol (peak 18, RT 19.86 min). Here, it was shown that *CcLAT*, when co-expressed with *CcCLS* and *CcLDDS1*, was able to metabolize both substrates and produce the acetylated derivatives, namely labda-7,13(*E*)-dien-15-yl acetate (peak 17, RT 19.62 min) and labda-13(*E*)-ene-8*α*-ol-15-yl acetate (peak 19, RT 20.37 min), as confirmed with the use of standards (Fig. 8). These observations suggest that, *in planta*, CcLAT is actively involved in the final stage of the biosynthetic pathway of labdane-type diterpenoids, yielding acetylated labdanes.

### Tissue and developmental expression of genes involved in the biosynthesis of labdane-related diterpenoids

**Fig. 9.**
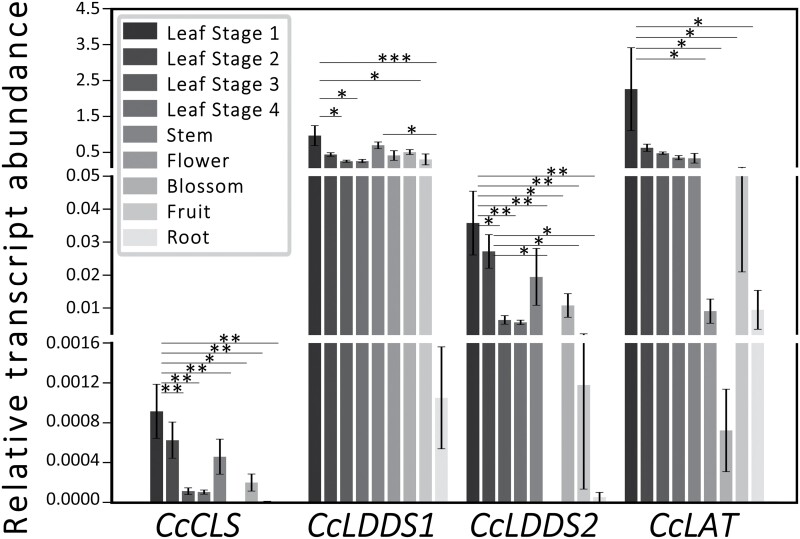
Relative transcript abundance of *CcCLS*, *CcLDDS1*, *CcLDDS2*, and *CcLAT* mRNAs in *C. creticus* subsp. *creticus* leaf stages 1–4, stem, flower, blossom, fruit, and root. Transcript abundance values were measured using qRT-PCR analysis, expressed in arbitrary units (AU), and normalized using *C. creticus* endogenous genes of actin (*CcActin*) and elongation factor a (*CcELFa*). Error bars represent the SD of three biological and two technical replicates. Asterisks denote significant differences between two indicated values (*P<0.05, **P<0.01, ***P<0.001, confidence level=0.95) based on Student’s *t*-test.

To study the regulation of gene expression, qPCR analysis was achieved by monitoring *CcLDDS1*, *CcLDDS2*, and *CcLAT* genes in various tissues of *C. creticus* subsp. *creticus*, including leaf stages 1–4, stem, flower, blossom, fruit, and root (Fig. 9). Overall, the highest expression levels were uncovered in all aerial parts, predominantly in young leaves and stems, while roots exhibited little to no expression. *CcLAT* mRNA exhibited the highest accumulation among all studied genes, predominantly in young leaves of S1, followed by older leaves and stems. Among diTPS genes, *CcLDDS1* transcripts showed the highest expression levels, with greater accumulation in young leaves of S1, followed by stems and blossoms. *CcLDDS2* mRNA levels were mainly accumulated in young leaves (S1–S2), followed by stems and blossoms. Finally, *CcCLS* transcripts showed the least expression across all tissues (Fig. 9).

## Discussion

### Labdane-type diterpenoid biosynthesis

The elucidation of the enzyme activity of CcLDDS1, CcLDDS2, and CcLAT has greatly contributed towards advancing our understanding of the catalytic landscape involved in labdane-type diterpenoid production in *C. creticus* subsp. *creticus*. Previous research in our lab on the *C. creticus* subsp. *creticus* trichome-specific EST library led to the *in vitro* characterization of CcCLS, which was the first characterized class II diTPS enzyme producing oxygen-containing labdane-type diterpenoids (Falara *et al.*, 2010). In this study, we report the functional characterization of three novel genes: (i) *CcLDDS1* and *CcLDDS2*, two class II *endo*-7,13-CPP synthase-encoding genes, and (ii) *CcLAT,* a BAHD alcohol acetyltransferase-encoding gene involved in the biosynthesis of labda-7,13(*E*)-dien-15-yl acetate and labda-13(*E*)-ene-8*α*-ol-15-yl acetate.

Yeast expression of the *CcLDDS1* and *CcLDDS2* genes verified the production of labda-7,13(*E*)-dien-15-ol followed by additional minor levels of *endo*-7,13-CPP structurally related labdane-type diterpenoids, namely labda-7,13(16),14-triene, a non-identified labdane, labda-7,12,14-triene, and labda-7,14-dien-13-ol, which were all formed by the absence of a class I diTPS gene. These compounds were also produced by SmCPSKSL1, a bifunctional, class I/II, labda-7,13(*E*)-dien-15-ol synthase (Mafu *et al.*, 2011) isolated from the lycophyte *S. moellendorffii* and by HsTPS1, a monofunctional, class II, *endo*-7,13-CPP synthase (Johnson *et al.*, 2019) isolated from *Hyptis suaveolens*, a member of the mint family.

To the best of our knowledge, no monofunctional class I diTPS producing labda-7,13(*E*)-dien-15-ol, labdatriene(s), and/or labda-7,14-dien-13-ol(s) has yet been identified in plants. The bacterial SCLAV_p0491 is the only currently characterized labda-7,13(16),14-triene synthase (*Streptomyces clavuligerus*, Yamada *et al.*, 2016). Successful combinatorial biosynthesis of labda-7,13(16),14-triene, labda-7,12,14-triene, and labda-7,14-dien-13-ol by monofunctional, plant and bacterial, class I diTPSs, using *endo*-7,13-CPP as a substate, has been reported (Jia *et al.*, 2016, 2019; Yamada *et al.*, 2016; Johnson *et al.*, 2019).

Production of labdane-type diterpenoids, namely labda-13(*E*)-ene-8*α*,15-diol, (13*R*) and (13*S*) manoyl oxide epimers, (13*R*) and (13*S*) sclareol epimers, 13(16)-14-labdien-8-ol, and putative copalol, has been reported by the expression of 8-OH-CPP synthase (or copal-8-ol diphosphate synthase), in the presence of phosphatases, without class I TPS-mediated enzymatic reaction (Caniard *et al.*, 2012; Schalk *et al.*, 2012; Pateraki *et al.*, 2014; Ignea *et al.*, 2015; Kim *et al.*, 2015; Papaefthimiou *et al.*, 2019).

Experimental parameters, such as pH conditions during the extraction process, affect scaffold stability, resulting in the non-enzymatic formation of labdanes. An illustration of this is the generation of sclareol by acid hydrolysis and/or manoyl oxide isomers by acid dehydration of 8-OH-CPP (LDPP) (Schalk *et al.*, 2012).

Minor amounts of labda-7,13(*E*)-dien-15-ol were identified by GC-MS analysis of the *in vivo* expression of the *N. glutinosa* copal-8-ol diphosphate synthase gene (*NgCLS*) (Criswell *et al.*, 2012). Furthermore, *in vitro* experiments using the *S. sclarea* copal-8-ol diphosphate synthase (SsLPPS) coupled with the class I sclareol synthase (SsSS) showed the presence of manool in minor quantities, suggesting that its substrate, (+)-CPP, is produced in trace amounts (Caniard *et al.*, 2012).

A previously reported mutagenic study aimed at identifying the catalytic base responsible for the class II bifunctional active site of abietadiene synthase (AgAS) of the gymnosperm *Abies grandis. *That study revealed the ability of mutant AgAS:H348A/D621A to produce 8-OH-CPP, (+)-CPP, and *endo*-7,13-CPP intermediate metabolites (Criswell *et al.*, 2012). Similar observations were also made in different site-directed studies of *Oryza sativa syn*-CPP synthase (OsCPS4, Potter *et al.*, 2016) and *Salvia divinorum* clerodienyl diphosphate synthase (SdCPS2) (Pelot *et al.*, 2017). Specifically, the *OsCPS4* mutant *H501* was found to produce *syn*-halimadienyl diphosphate (Potter *et al.*, 2016), while modifications of four catalytic residues in the sequence of SdCPS2 enabled the production of four distinct products (Pelot *et al.*, 2017).

These findings provide insight into the enzymatic plasticity of plant TPSs. Based on these observations, we hypothesized that the genes *CcCLS*, *CcLDDS1*, and *CcLDDS2*, when expressed in yeast (Fig. 7), could also produce an array of labdane-type compounds, albeit in minor amounts, due to low catalytic specificity. To test this hypothesis, a modified protocol was followed, where incubation time and volume of yeast cultures were prolonged and augmented, respectively, which by a comparative analysis of the products, revealed the presence of *endo*-7,13-CPP-, 8-OH-CPP-, and (+)-CPP-related metabolites, in varying amounts, demonstrating a surprising enzymatic plasticity.

### Putative biosynthetic pathway of labda-7,13(*E*)-dien-15-yl acetate and labda-13(*E*)-ene-8*α*-ol-15-yl acetate in *C. creticus* subsp. *creticus* trichomes

**Fig. 10.**
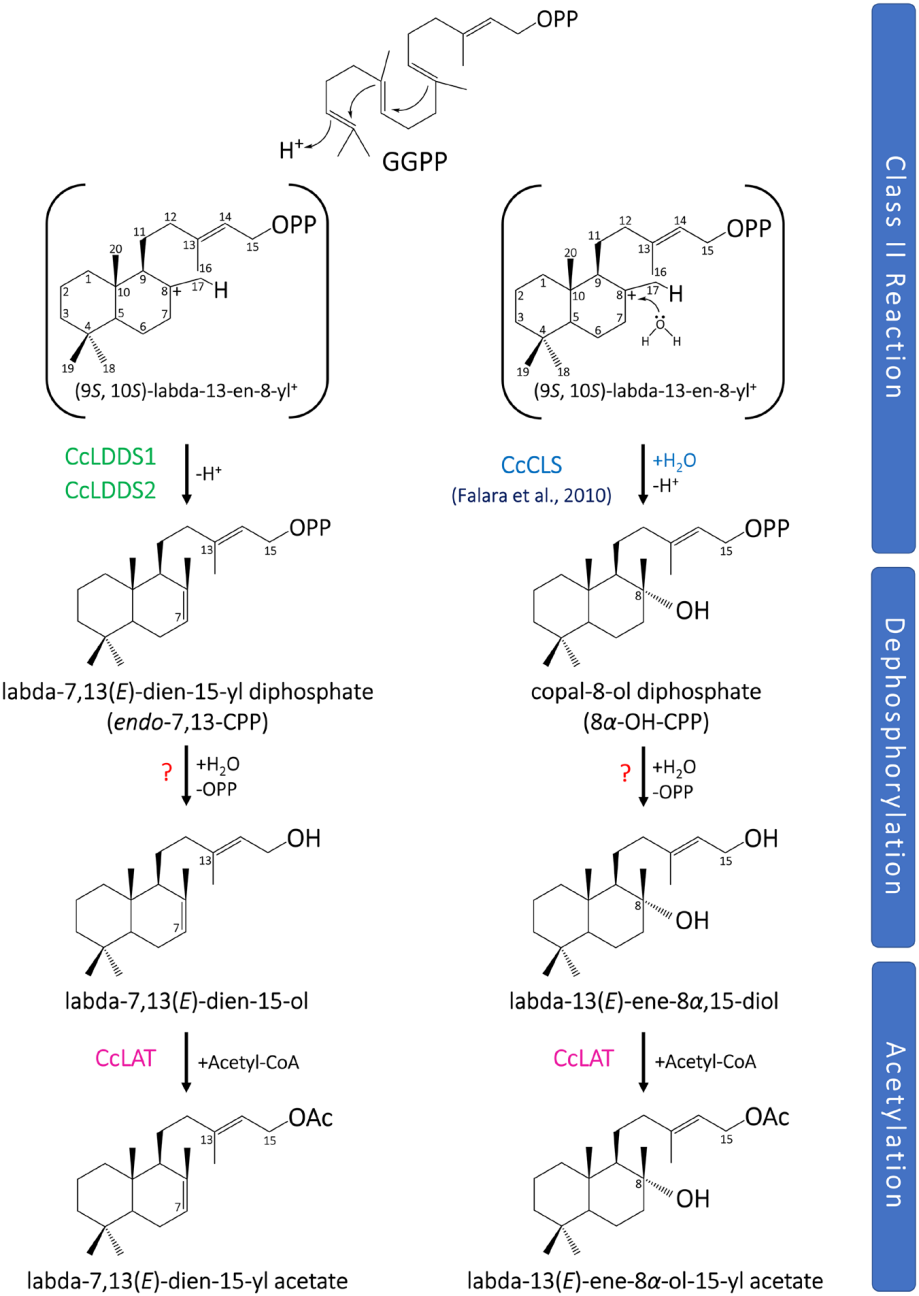
Proposed pathway of labda-7,13(*E*)-dien-15-yl acetate and labda-13(*E*)-ene-8*α*-ol-15-yl acetate produced in *C. creticus* subsp. *creticus*. The first committed step in labdane-type diterpenoid biosynthesis involves the formation of a diphosphate ester with the characteristic *trans*-decalin labdanic structure. In *C. creticus* subsp. *creticus*, class II labdane-type diTPSs (DxDD) catalyze the protonation-initiated cyclization of (*E*,*E*,*E*)-GGPP via formation of the labda-13-en-8-yl^+^ intermediate. Class II enzymes CcLDDS1 and CcLDDS2 terminate the carbocations via deprotonation at C-7, leading to the formation of *endo*-7,13-CPP. An alternative route catalyzed by CcCLS (Falara *et al.*, 2010) involves the incorporation of a water molecule prior to deprotonation, yielding the hydroxylated product copal-8-ol diphosphate (8-OH-CPP). In a subsequent step, a group of as yet unknown enzymes facilitate the removal of the diphosphate group, resulting in the production of labda-7,13(*E*)-dien-15-ol and labda-13(*E*)-ene-8*α*,15-diol. Finally, a labdane acetyltransferase of the BAHD superfamily, namely CcLAT, catalyzes the conversion of labda-7,13(*E*)-dien-15-ol and labda-13(*E*)-ene-8*α*,15-diol into their acetylated derivatives: labda-7,13(*E*)-dien-15-yl acetate and labda-13(*E*)-ene-8*α*-ol-15-yl acetate.

Class II enzymes CcLDDS1, CcLDDS2, and CcCLS, using GGPP as a substate, catalyze the first committed step resulting in the formation of the characteristic labdane-type scaffold of *endo*-7,13-CPP and 8*α*-OH-CPP diphosphate intermediates (Fig. 10). Subsequently, a hydrolysis reaction, catalyzed by an as yet unknown enzyme, results in the removal of the diphosphate moiety of *endo*-7,13-CPP and 8*α*-OH-CPP intermediates, which finally results in the production of derivatives, namely labda-7,13(*E*)-dien-15-ol and labda-13(*E*)-ene-8*α*,15-diol, respectively. Further research is required to determine whether this hydrolysis reaction is catalyzed by a TPS-based or an alternative phosphatase-based route (Fig. 10).

The production of labda-7,13(*E*)-dien-15-ol and labda-13(*E*)-ene-8*α*,15-diol by a monofunctional class I TPS has not yet been reported. The bifunctional enzyme SmCPSKSL1, characterized in the lycophyte *S. moellendorffii*, converts GGPP to *endo*-7,13-CPP and subsequently to labda-7,13(*E*)-dien-15-ol through a combination of class II and class I TPS-mediated reactions (Mafu *et al.*, 2011). However, no class I enzyme associated with the production of labda-7,13(*E*)-dien-15-ol and labda-13(*E*)-ene-8*α*,15-diol has been identified in *C. creticus* yet.

Terpenoid production in plants typically follows a ‘canonical’ TPS-based pathway. However, an alternative, phosphatase-based, TPS-independent pathway has recently been identified (Sun *et al.*, 2016). In *Grindelia robusta*, the formation of labda-7,13(*E*)-dien-15-ol from *endo*-7,13-CPP has been proposed to occur through the activity of endogenous phosphatases rather than class I TPS activity (Zerbe *et al.*, 2015). Similarly, no class I enzyme producing labda-13(*E*)-ene-8*α*,15-diol has been characterized yet, to the best of our knowledge. It is plausible to hypothesize that the production of labda-7,13(*E*)-dien-15-ol and labda-13(*E*)-ene-8*α*,15-diol from *endo*-7,13-CPP and 8-OH-CPP, respectively, could potentially be catalyzed by an endogenous phosphatase, a Nudix hydrolase, or even a combination of these enzymatic activities. Nudix hydrolases have demonstrated diphosphohydrolase activity, eliminating the first phosphate of terpenoid diphosphate substrates (Magnard *et al.*, 2015; Henry *et al.*, 2018; Liu *et al.*, 2018; Li *et al.*, 2020; Sun *et al.*, 2020; Bergman *et al.*, 2021).

The final step of the pathway, leading to the production of labda-7,13(*E*)-dien-15-yl acetate and labda-13(*E*)-ene-8*α*-ol-15-yl acetate, is catalyzed by a BAHD alcohol acetyltransferase (Fig. 10). The presence of an acetyl group has been linked with enhanced biological activities (Chinou *et al.*, 1994). Acetylated labdanes are compounds rarely identified in plants. To the best of our knowledge, the only other known labda-7,13(*E*)-dien-15-yl acetate has been reported in *Salvia leriifolia* root essential oil, a plant of the *Lamiaceae* family endemic to Iran (Sarhadi *et al.*, 2021). Labda-13(*E*)-ene-8*α*-ol-15-yl acetate has been identified in various *Cistus* species, including *Cistus ladanifer* (Mariotti *et al.*, 1997), *Cistus monspeliensis* (Angelopoulou *et al.*, 2002), *Cistus parviflorus* (Angelopoulou *et al.*, 2001), and *Cistus salviifolius* (Demetzos *et al.*, 2002). Additionally, this compound has been found in *Croton ciliatoglanduliferus* (Morales-Flores *et al.*, 2007) and *Psiadia arguta* (Mahadeo *et al.*, 2020).

This study provides insights into the catalytic activity of the BAHD alcohol acetyltransferase CcLAT in the acetylation reaction of labda-7,13(*E*)-dien-15-ol and labda-13(*E*)-ene-8*α*,15-diol using the yeast expression system for pathway reconstitution (Fig. 8). Future research should focus on unraveling the biosynthetic pathway of less explored *ent*-labdanes, such as *ent*-3*β*-acetoxy-13-*epi*-manoyl oxide. Furthermore, the potential involvement of Nudix hydrolases in the dephosphorylation reaction of labdane-type diterpenoid diphosphate esters should also be investigated further.

### Tissue-specific accumulation of labdane-related diterpenoids and corresponding gene expression

This study examined the accumulation of LRDs (Figs 1, 2; Supplementary Fig. S1; Supplementary Table S1) and the relative expression patterns of *CcCLS*, *CcLDDS1*, *CcLDDS2*, and *CcLAT* genes in various plant tissues, including leaves, stems, flowers, blossoms, fruits, and roots (Fig. 9). The biosynthesis of LRDs predominantly takes place in the glandular capitate trichomes covering the aerial parts of the plant. The higher expression of genes correlated with the increased accumulation of metabolic products in young leaves, suggesting tissue and developmental control.

The relative expression levels of *CcLDDS1*, *CcLDDS2*, and *CcLAT* genes (Fig. 9) correlated with the production of labda-7,13(*E*)-dien-15-ol (17.04%) and its acetylated derivative, labda-7,13(*E*)-dien-15-yl acetate (40.75%) (Fig. 1). Notably, *CcLDDS1* exhibited higher overall expression among diTPS-encoding genes across all examined tissues. Additionally, the low relative expression of the *CcCLS* gene (Fig. 9) coincided with the low accumulation of its dephosphorylated metabolic product, namely labda-13(*E*)-ene-8*α*,15-diol (3.80%) (Fig. 1). Conclusively, all genes appear to be developmentally regulated and exhibit a tissue-specific pattern of expression. These results align with previous reported findings on *CcCLS* transcript accumulation and the production of major LRDs in *C. creticus* subsp. *creticus* tissues (Falara *et al.*, 2010), providing valuable insights into the correlation between gene expression and metabolite accumulation.

In conclusion, this study significantly advances our understanding of the biosynthetic pathways in *C. creticus* subsp. *creticus*, a non-model plant, by characterizing *CcLDDS1* and *CcLDDS2*, two class II *endo*-7,13-CPP-encoding genes and *CcLAT*, a BAHD alcohol acetyltransferase-encoding gene involved in the biosynthesis of labda-7,13(*E*)-dien-15-yl acetate and labda-13(*E*)-ene-8*α*-ol-15-yl acetate. Labdane-type diterpenoids, identified in the plant’s resin known as ‘Ladano’, are bioactive compounds characterized by a distinct decalin–labdanic scaffold. Acetylated labdanes are rarely produced by plants and have been shown to exhibit enhanced antibacterial, antifungal, and cytotoxic activities. This study presents novel labdane-type diterpenoids, including *cis*-abienol, neoabienol, *ent*-copalol, and one as yet unidentified labdane, expanding our knowledge of the plant’s secondary reservoir. These findings lay the groundwork for future research, in particular the exploration of the biosynthetic pathway of production of labdane-type diterpenoids such as *ent*-13-*epi*-manoyl oxide, *ent*-3*β*-hydroxy-13-*epi*-manoyl oxide, and *ent*-3*β*-acetoxy-13-*epi*-manoyl oxide in *C. creticus* subsp. *creticus.* Labdanes present favorable physicochemical and biological properties that could potentially be used in pharmaceutical and crop protection applications. The successful reconstitution of acetylated labdanes in the yeast expression system marks an initial step towards large-scale and sustainable industrial production. In a nutshell, this work has not only further elucidated the steps of labdane-type diterpenoid biosynthesis, but it has also provided the basis for future research in the field of pharmaceutical crops.

## Supplementary data

The following supplementary data are available at *JXB* online.

Fig. S1. Total ion current (TIC) chromatograms of GC-MS analysis of *n*-hexane extracts of *C. creticus* subsp. *creticus* tissues, namely leaf stages 1–4, stem, flower, blossom, fruit, and root.

Fig. S2. Mass spectra of peaks 1–19 and their comparison with the FFNSC 2 GC-MS library.

Fig. S3. Functional classification of unigenes derived from RNA-seq of *C. creticus* subsp. *creticus* trichomes.

Fig. S4. ^13^C spectrum of labda-7,13(*E*)-dien-15-ol.

Fig. S5. ^1^H spectrum of labda-7,13(*E*)-dien-15-ol.

Fig. S6. COZY spectrum of labda-7,13(*E*)-dien-15-ol.

Fig. S7. HMBC spectrum of labda-7,13(*E*)-dien-15-ol.

Fig. S8. HSQC spectrum of labda-7,13(*E*)-dien-15-ol.

Fig. S9. Alignment of CcLDDS2, contig 936, and contig 541 produced by the ClustalW algorithm utilizing the AlignX program from the Vector NTI software package.

Fig. S10. Amino acid sequence alignment of the class II labdane-type diterpenoid synthases CcCLS (Falara *et al.*, 2010), CcLDDS1, and CcLDDS2 characterized in *C. creticus* subsp. *creticus*.

Fig. S11. Protein sequence alignment of BAHD acetyltransferases.

Table S1. List of labdane-related diterpenoids (LRDs) identified in *n*-hexane extracts from various tissues of *C. creticus* subsp. *creticus* by GC-MS analysis.

Table S2. Retention index (RI) values calculated for each of the 19 peaks identified in this study compared with their corresponding RI values as cited in GC/MS libraries (FFNSC 2; Adams, 2007; massfinder 3) and the literature.

Table S3. List of known plant diterpenoid synthases (diTPSs) that were used for the phylogenetic analysis of CcLDDS1 and CcLDDS2.

Τable S4. RNA-seq data analysis of *C. creticus* subsp. *creticus* trichomes highlighting translated contigs sharing strong sequence similarity to known class II diterpenoid synthases (diTPSs).

Table S5. List of BAHD acetyltransferases (Clades I–V) according to D’Auria (2006) used for *C. creticus* subsp. *creticus* labdane acetyltransferase (CcLAT) phylogenetic analysis.

Table S6. List of primers used for gene cloning in this study (manually designed).

Table S7. NMR analysis of labda-7,13(*E*)-dien-15-ol.

Table S8. List of primers used for qRT-PCR analysis (manually designed).

Table S9. Summary of Contig and Unigene statistics of RNA-seq data analysis from *C. creticus* subsp. *creticus* trichomes isolated from leaves of developmental stage 2.

Table S10. CcCLS (Falara *et al.*, 2010), CcLDDS1, and CcLDDS2 protein sequence similarity (%) (BLAST-p, NCBI).

erae098_suppl_Supplementary_Figures_S1-S11_Tables_S1-S10

## Data Availability

Nucleotide sequences of enzymes reported in this paper have been deposited at the NCBI GenBank™ under the accession numbers: CcACTIN (MT674938), CcLDDS1 (MT666220), CcLDDS2 (MT666221), and CcLAT (MT666224). Transcriptome assembly has been deposited at the ENA-EBI under the BioProject PRJEB71924.
