# Supplementary material for: Chemical and transcriptomic analyses of leaf trichomes from Cistus creticus subsp. creticus reveal the biosynthetic pathways of certain labdane-type diterpenoids and their acetylated forms
Source: J Exp Bot. 2024 Mar 23;75(11):3431–51. doi: 10.1093/jxb/erae098 (PMC11156806; doi:10.1093/jxb/erae098)
Supplement: erae098_suppl_Supplementary_Figures_S1-S11_Tables_S1-S10 [file erae098_suppl_supplementary_figures_s1-s11_tables_s1-s10.pdf]

Journal of Experimental Botany

Chemical and transcriptomic analyses of leaf trichomes from *Cistus creticus* subsp. *creticus* reveal the biosynthetic pathways of certain labdane-type diterpenoids and their acetylated forms

Antigoni S. Papanikolaou, Dimitra Papaefthimiou, Dragana Matekalo, Christina-Vasiliki Karakousi, Antonios M. Makris and Angelos K. Kanellis

Supplementary Data

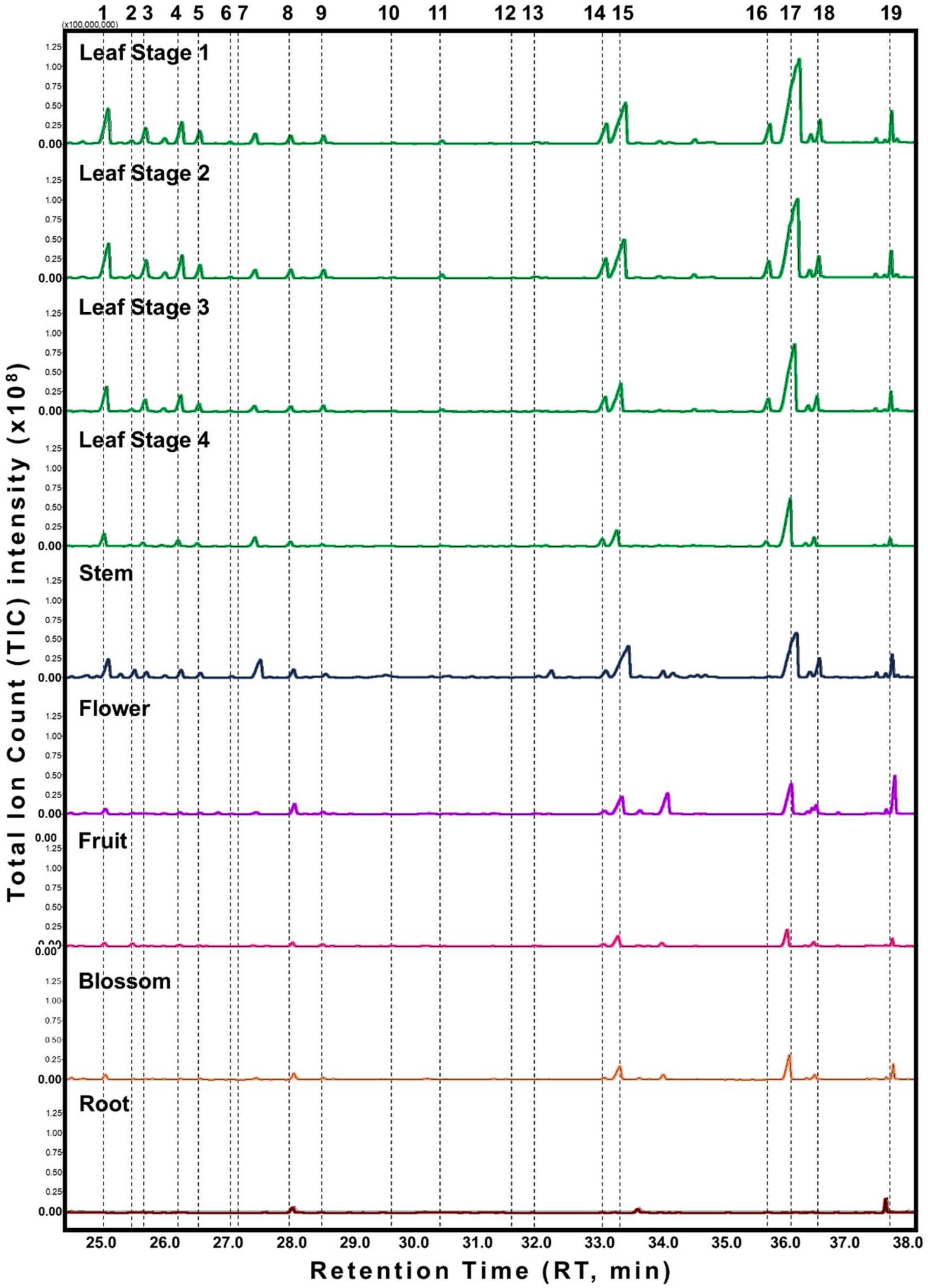

Figure S1. Total ion current (TIC) chromatograms of GC-MS analysis of *n*-hexane extracts of *C. creticus* subsp. *creticus* tissues, namely leaf stages 1-4, stem, flower, blossom, fruit, and root. Peaks 1-19 detected correspond to equal numbers of labdane-related diterpenoids (LRDs) metabolites produced.

Peaks 1-19 mass spectra ⟷ FFNSC 2 library 1st hit result

Peak 1: labda-7,13(16),14-triene, MW=272

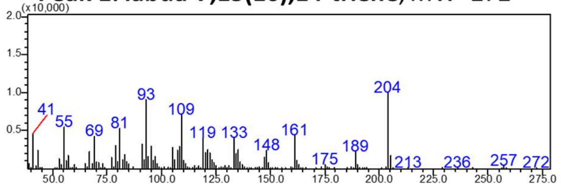

labda-7,13(*E*)-dien-15-ol (similarity 90%)

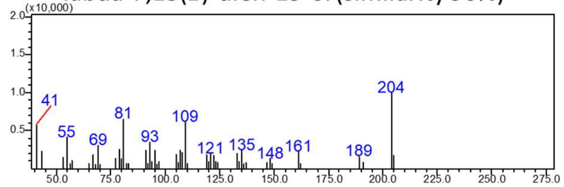

Peak 2: non-identified

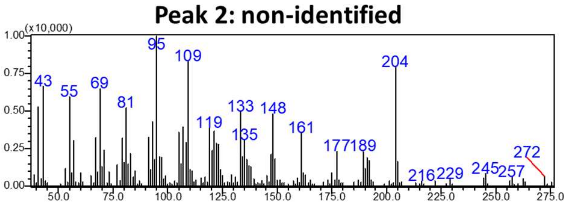

labda-7,13(*E*)-dien-15-ol (similarity 87%)

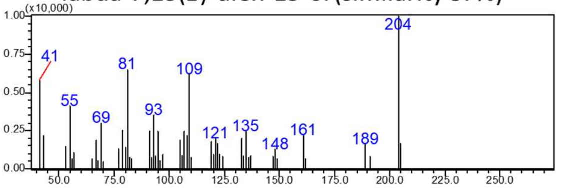

Peak 3: manoyl oxide, MW=290

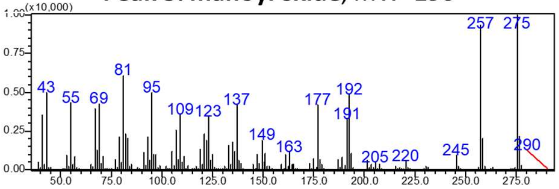

manoyl oxide (similarity 93%)

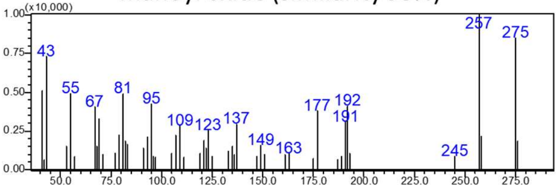

Peak 4: 13-*epi*-manoyl oxide, MW=290

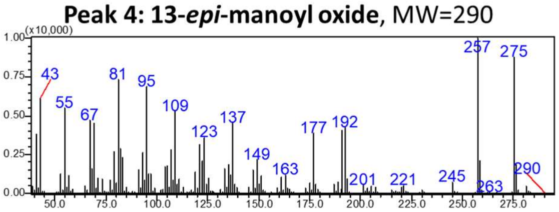

manoyl oxide (similarity 95%)

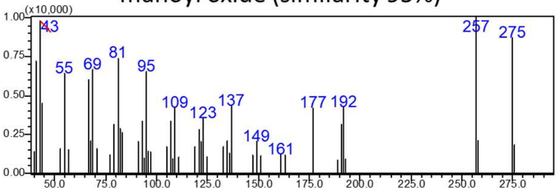

Peak 5: labda-7,12,14-triene, MW=272

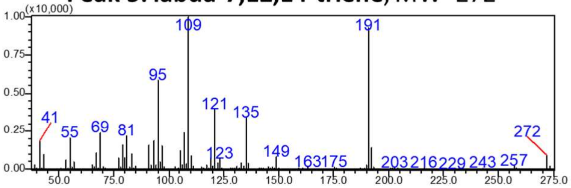

iso E super <gamma->

Peak 6: *ent*-kaurene, MW=272

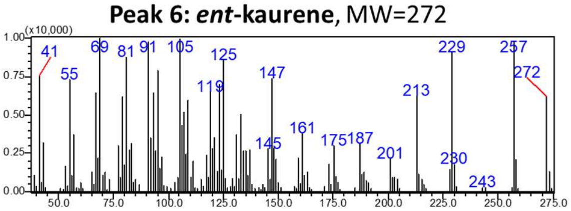

kaurene (similarity 86%)

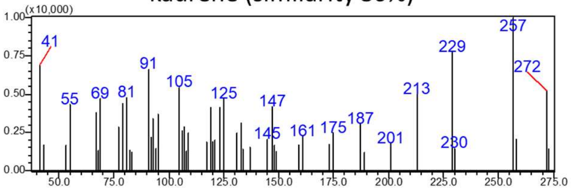

Peak 7: manool, MW=290

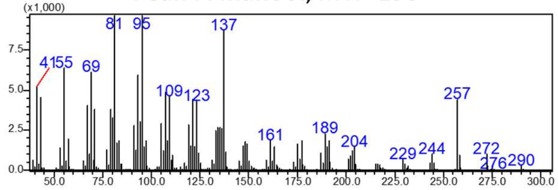

manool (similarity 82%)

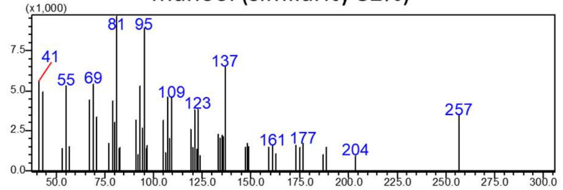

continued on the next page…

Peaks 1-19 mass spectra ⟷ FFNSC 2 library 1st hit result

Peak 8: labda-7,14-dien-13-ol, MW=290

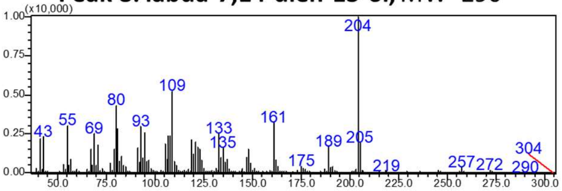

labda-7,13(*E*)-dien-15-ol (similarity 90%)

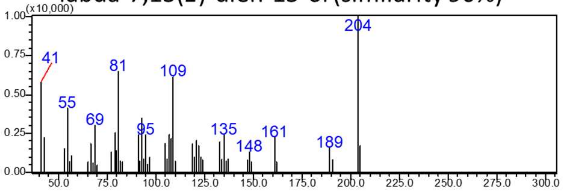

Peak 9: 13(16),14-labdien-8-ol, MW=290

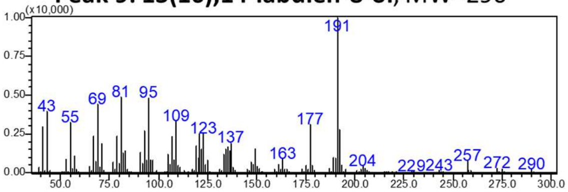

labda-13(*E*)-ene-8*α*,15-diol (similarity 84%)

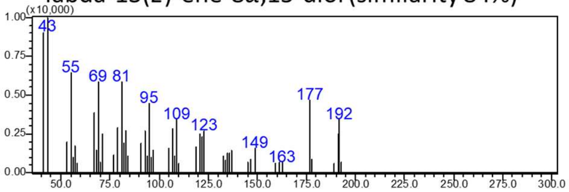

Peak 10: neoabienol, MW=290

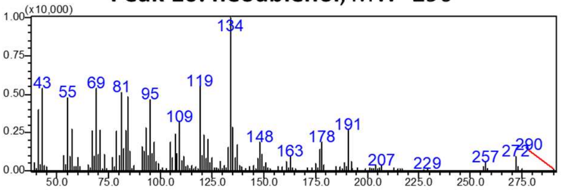

abienol (similarity 92%)

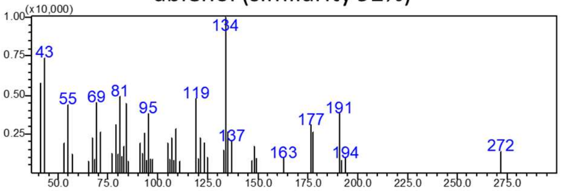

Peak 11: *cis*-abienol, MW=290

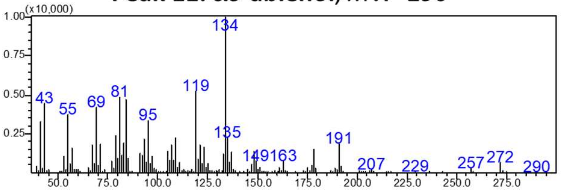

abienol (similarity 91%)

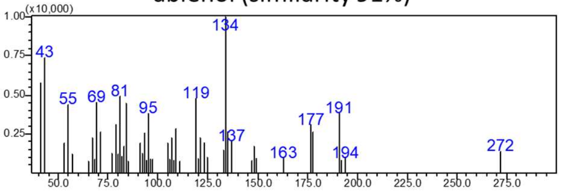

Peak 12: sclareol, MW=308

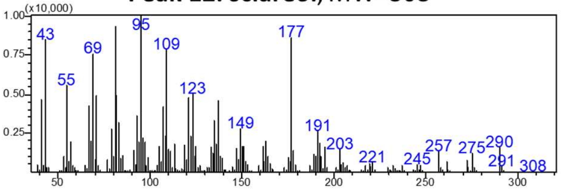

sclareol (similarity 86%)

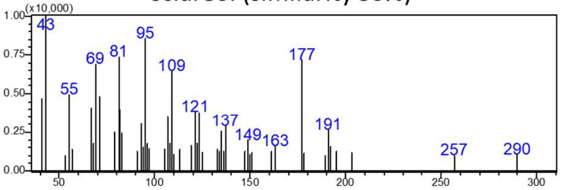

Peak 13: *ent*-copalol, MW=290

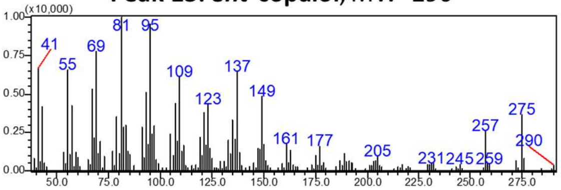

manool (similarity 86%)

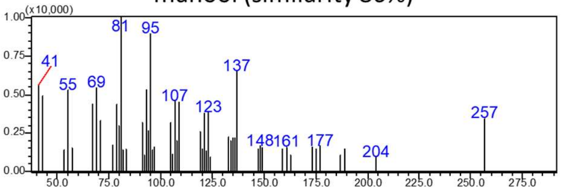

Peak 14: 3*β*-hydroxy-13-*epi*-manoyl oxide, MW=306

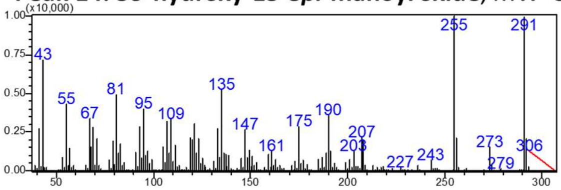

no hit compound

continued on the next page…

Peaks 1-19 mass spectra ⟷ FFNSC 2 library 1st hit result

Peak 15: labda-7,13(*E*)-dien-15-ol, MW=290

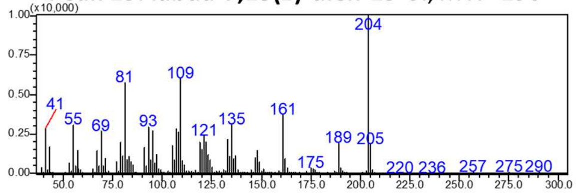

labda-7,13(*E*)-dien-15-ol (similarity 91%)

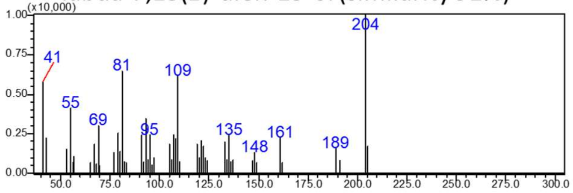

Peak 16: 3*β*-acetoxy-13-*epi*-manoyl oxide, MW=348

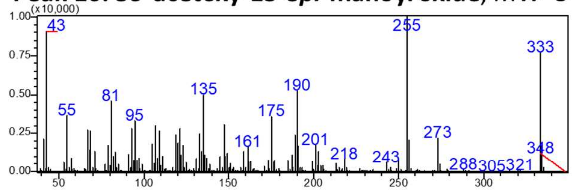

no hit compound

Peak 17: labda-7,13(*E*)-dien-15-yl acetate, MW=332

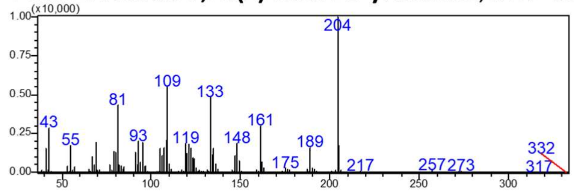

labda-7,13(*E*)-dien-15-ol (similarity 89%)

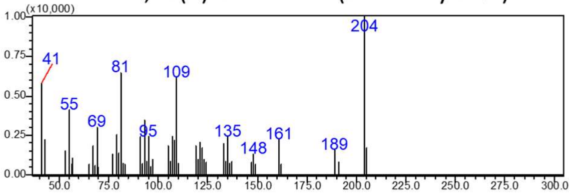

Peak 18: labda-13(*E*)-ene-8*α*,15-diol, MW=308

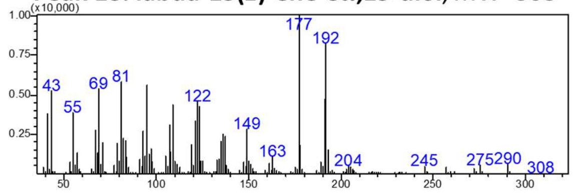

labda-13(*E*)-ene-8*α*,15-diol (similarity 86%)

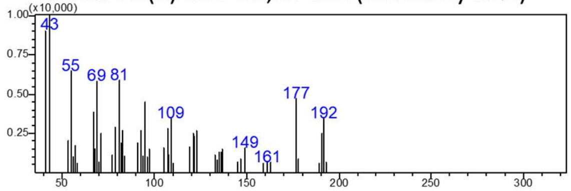

Peak 19: labda-13(*E*)-ene-8*α*-ol-15-yl acetate, MW=350

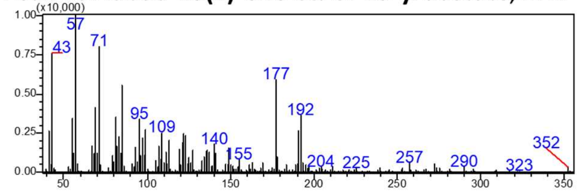

no hit compound

Figure S2. Mass spectra of peaks 1-19 and their comparison with the FFNSC 2 GC-MS library. Left column: mass spectra and molecular weight (MW) of the 19 peaks identified in this study. Right column: 1st hit result of the FFNSC 2 GC-MS library (FFNSC 2, 2012).

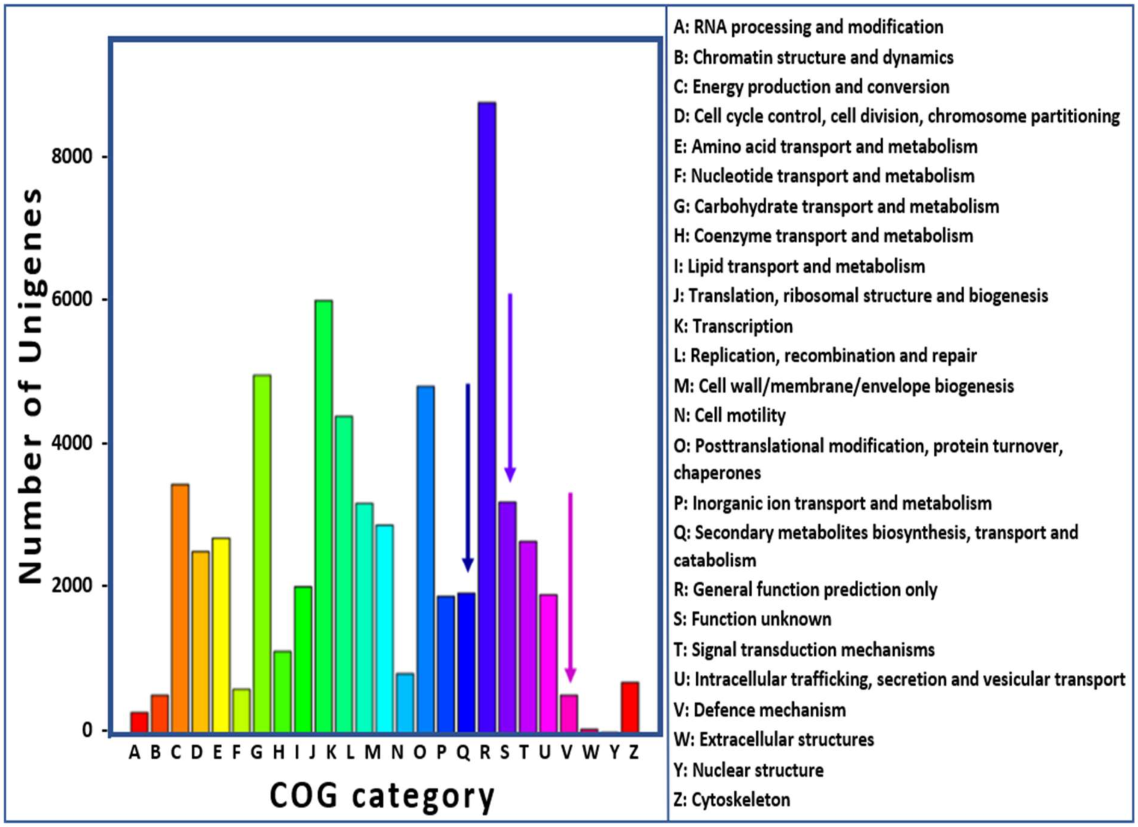

Figure S3. Functional classification of unigenes derived from RNA-seq of *C. creticus* subsp. *creticus* trichomes. Annotations were obtained using BLAST-x (E-value < 10^{-5}) search against the Clusters of Orthologous Group (COG) database.

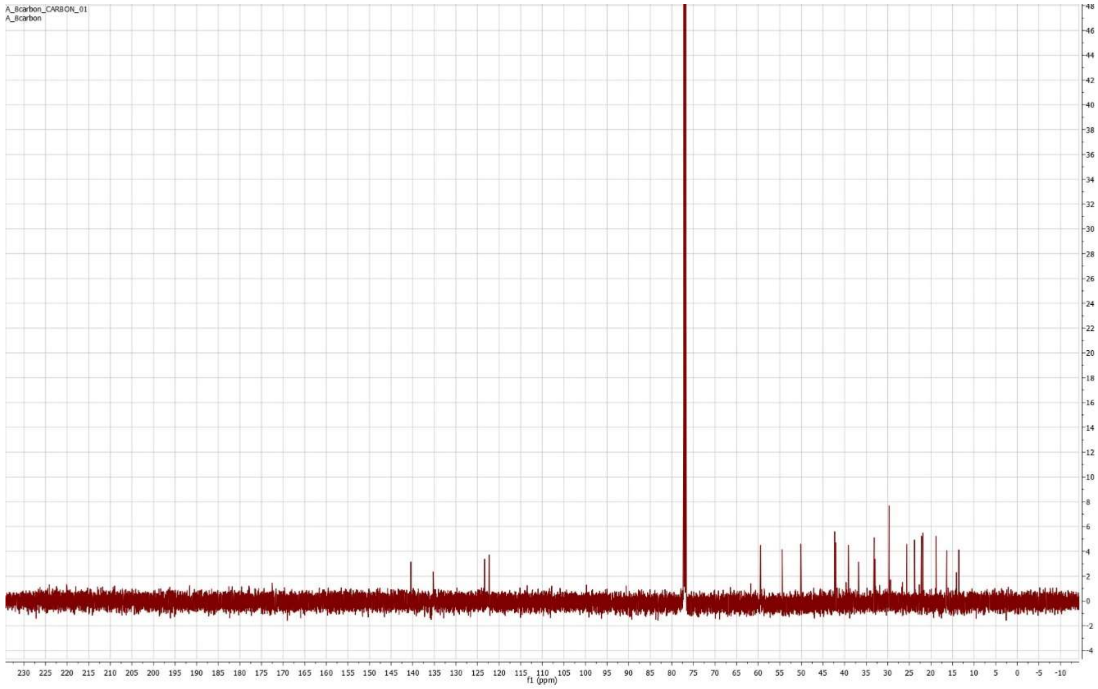

Figure S4. ^{13}C spectrum of labda-7,13(*E*)-dien-15-ol.

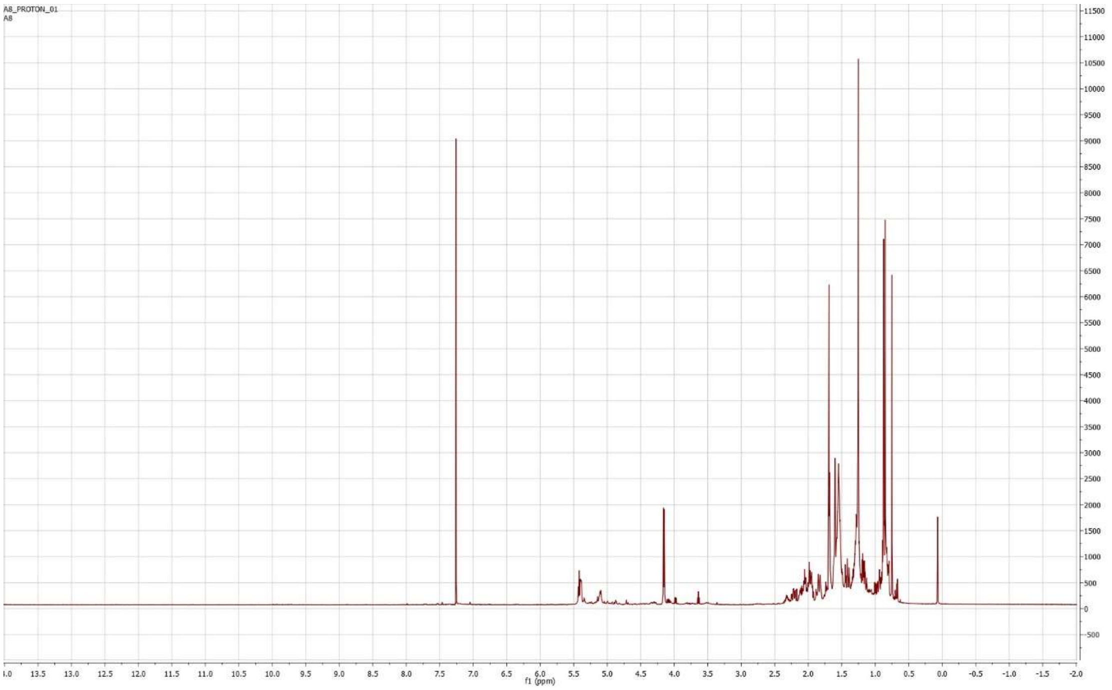

Figure S5. ^{1}H spectrum of labda-7,13(*E*)-dien-15-ol.

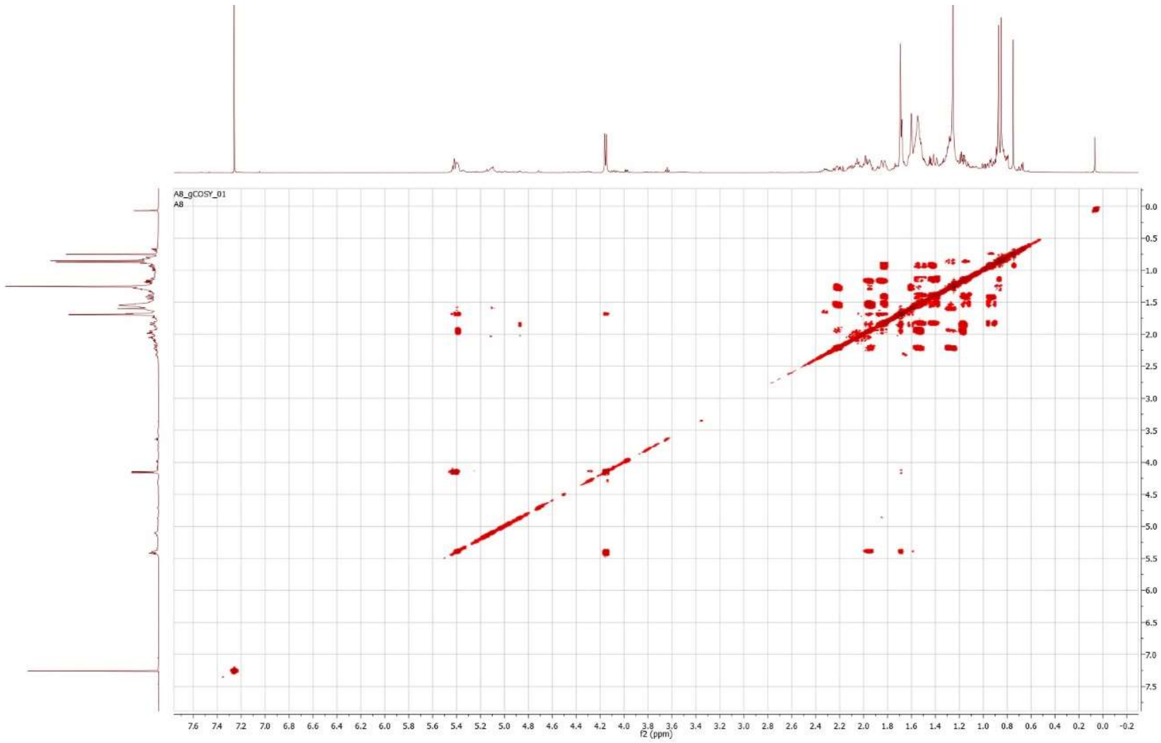

Figure S6. COZY spectrum of labda-7,13(*E*)-dien-15-ol.

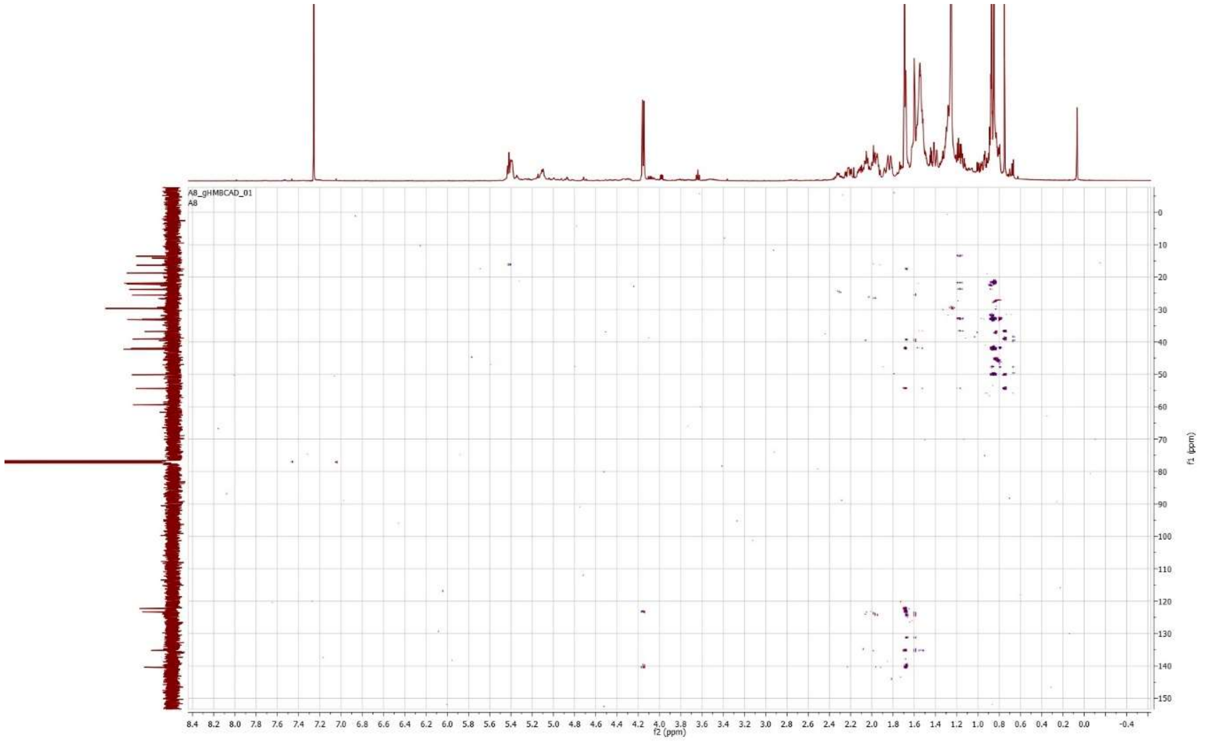

Figure S7. HMBC spectrum of labda-7,13(*E*)-dien-15-ol.

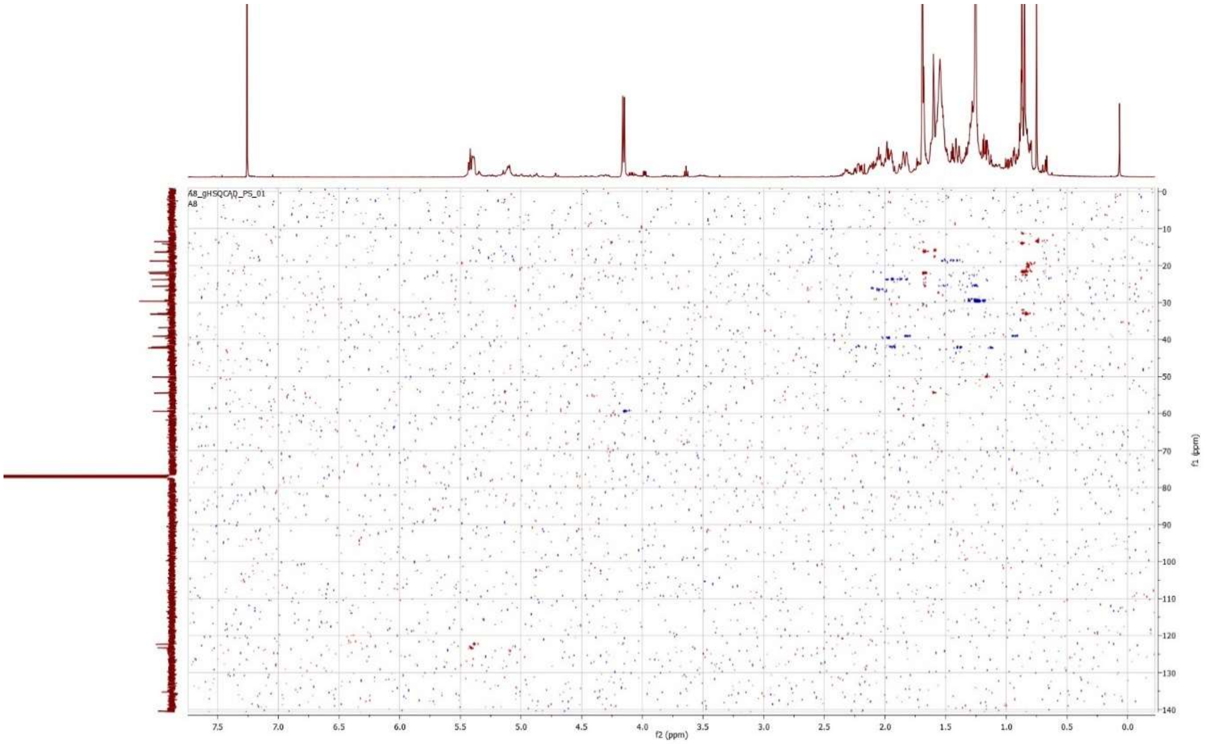

Figure S8. HSQC spectrum of labda-7,13(*E*)-dien-15-ol.

```
                    1                                                                   70
  CcLDDS2    (1) ----MMASTLALANLFHQVTTNHSIHVNYPFQPGNWLLLAKVKGRGKFDTRLRCSGATPKSPAQGSITKD
936contig    (1) ENHIMMASTLALANLFHQVTTNHSIHVNYPFQPGNWLLLAKVKGRGTFDTRLRCSGATPKSPAQGSITKD
541contig    (1) ----------------------------------------------------------------------
                 71                                                                  140
  CcLDDS2   (67) ISLNSDAGGDGVVEAKQRLQTIADDKTNGGADDLGIAVSDRVRDGVDALKSMFNSMGGGEINVSAYDTAW
936contig   (71) ISLNSDAGGDGVVEAKQRLQTIADDKTNGGADDLGIAVSDRVRDGVDALKSMFNSMGGGEINVSAYDTAW
541contig    (1) ----------------------------------------------------------------------
                 141                                                                 210
  CcLDDS2  (137) IALVKDVNGSGGPQFPSSLQWIVDNQLPDGSWGDGQLFSAYDRLLNTLACVVALKSWNIRPDKYEKGLKF
936contig  (141) IALVKDVNGSGGPQFPSSLQWIVDNQLPDGSWGDGQLFSAYDRLLNTLACVVALKSWNIRPDKYEKGLKF
541contig    (1) ----------------------------------------------------------------------
                 211                                                                 280
  CcLDDS2  (207) LKENISKLEKENAEASEQMLTGFEVVFLSLVDIARGLDIHIISLDSPVFQDLTARRNLKFAKIPMDLMHN
936contig  (211) LKENISKLEEENEQHM------------------------------------------------------
541contig    (1) ----------------------------------------------------------------------
                 281                                                                 350
  CcLDDS2  (277) VPTSLLYSLEGFAELELDWEKLLKLQSRGGSFLSSPASTALALMQTKDKNCLEYLNDVVQKFNGGAPCQY
936contig  (227) ----------------------------------------------------------------------
541contig    (1) ---------------------------------------------------------------------Y
                 351                                                                 420
  CcLDDS2  (347) PVEIFERIWVIDRLQRLGISRYFQLEIKDCLDYVSKYWTQYGCSWEKDSRMCDLDDTCMAFRILRLHGYH
936contig  (227) ----------------------------------------------------------------------
541contig    (2) PVEIFERIWVIDRLQRLGISRYFQLEIKDCLDYVSKYWTQYGCSWEKDSRMCDLDDTCMAFRILRLHGYH
                 421                                                                 490
  CcLDDS2  (417) VSPEAFRHFENGGEFFCFPYQTTQSVTVNFNLYRATQVMFPGEKILEEAKQFSLNYLREKQAANKFLDKW
936contig  (227) ----------------------------------------------------------------------
541contig   (72) VSPEAFRHFENGGEFFCFPYQTTQSVTVNFNLYRATQVMFPGEKILEEAKQFSLNYLREKQAANKFLDKW
                 491                                                                 560
  CcLDDS2  (487) VILKDLPGEVEYALAVPWFASLPRVETRFYIEQYGGDNDVWIAKTFYRMGYVNNNVNLEVAKLDFNNCQA
936contig  (227) ----------------------------------------------------------------------
541contig  (142) VILKDLPGEVEYALAVPWFASLPRVETRFYIEQYGGDNDVWIAKTFYRMGYVNNNVNLEVAKLDFNNCQA
                 561                                                                 630
  CcLDDS2  (557) LHRIEWQAMQKWFVESKFCDFGASKNPLLIAYFLAASNIFEQERSVERLAWAKTTFLMEVIGSLFHDEVN
936contig  (227) ----------------------------------------------------------------------
541contig  (212) LHRIEWQAMQKWFVESKFCDFGASKNPLLIAYFLAASNIFEQERSVERLAWAKTTFLMEVIGSLFHDEVN
                 631                                                                 700
  CcLDDS2  (627) SRDQRGAFIQEFKTINAKFGEGHIHGRKIEAKGLKNNLITILFTTLNQLSLEAMVACGRDINPYLRHSWE
936contig  (227) ----------------------------------------------------------------------
541contig  (282) SRDQRGAFIQEFKTINAKFGEGHIHGRKIEAKGLKNNLITILFTTLNQLSLEAMVACGRDINPYLRHSWE
                 701                                                                 770
  CcLDDS2  (697) KWMMMWEQEGDMYKGAAELLVNTVNLCSGRLFSNDTLSHPNYERLVTLSNKVCHQLGNTLGGNHNEDSDI
936contig  (227) ----------------------------------------------------------------------
541contig  (352) KWMMMWK---------------------------------------------------------------
                 771                                                          833
  CcLDDS2  (767) KDTKIEIAMQELVQLVHQNSSDDISTDLKQTFFAVVRSFYYAAHCDQGTINSHIVKVLFESVV
936contig  (227) ---------------------------------------------------------------
541contig  (359) ---------------------------------------------------------------
```

Figure S9. Alignment of CcLDDS2, contig 936, and contig 541 produced by the ClustalW algorithm utilizing the AlignX program from the Vector NTI software package. The conserved DxDD motif is depicted in bold and underlined font (**D**:Asp: Aspartic Acid and **L**:Leu: Leucine). Color code reveals the degree of similarity between aligned amino acid sequences. Conservative areas are highlighted in cyan, while common amino acids are shown in blue font. Non-similar amino acids are shown in black font letters.

```
                 1                                                                    70
CcCLS      (1)   -MAFT-------FTSAHLFLPVTENHSVHVNYSIPPGNWRLWSTAKGGSNKLDIRRLRCSARRTPEPLA-
CcLDDS1    (1)   MLPSTSSSSLHFFLPPPPHHSYTTTNHHLIHFPSPPGIWRGRAKDKTRG-----NRRRCSATS-KS----
CcLDDS2    (1)   MMAST-------LALANLFHQVTTNHSIHVNYPFQPGNWLLLAKVKGRGKF--DTRLRCSGATPKSPAQG
                 71                                                                  140
CcCLS      (62)  --------QGSNGGRDGVEAIQRLQTIADDKIDGGANELGIVVWDLIRDGVDAVKSMFDSMGDGDISISA
CcLDDS1    (61)  --------------RVQVAGEYANVPIVDDEING-----AVDFEIPVKDRVDAVKSMLSSIDDGEVTASA
CcLDDS2    (62)  SITKDISLNSDAGGDGVVEAKQRLQTIADDKTNGGADDLGIAVSDRVRDGVDALKSMFNSMGGGEINVSA
                 141                                                                 210
CcCLS      (124) YDTAWVALVKDVNGSGGPQFPSSLQWIVDNQLPDGSWGDSEVFSAYDRLLKTLACVVALKSWNIRPDKCQ
CcLDDS1    (112) YDAAWLALVKDVNGGNGPQFPSCLRWIVENQLPDGSWGDGEIFLAHDRLINTLACVVALKSWNIHPDKCE
CcLDDS2    (132) YDTAWIALVKDVNGSGGPQFPSSLQWIVDNQLPDGSWGDGQLFSAYDRLLNTLACVVALKSWNIRPDKYE
                 211                                                                 280
CcCLS      (194) KGLKFFRDNISKLEKENVEASAQMLSGFEVVFLSLIEVARRLDIQ-IPLHSPVFEDLIARRNLKFAKIPL
CcLDDS1    (182) KGLNFFKENISKLEK---ENEQHMPIGFEVAFPSLLEVARRLNMIDICYDSPIFETINARRNLKLTKIPK
CcLDDS2    (202) KGLKFLKENISKLEKENAEASEQMLTGFEVVFLSLVDIARGLDIHIISLDSPVFQDLTARRNLKFAKIPM
                 281                                                                 350
CcCLS      (263) DLMHNVPTSLLNSLEGMTGVELDWEKLLKLQSQDGSFITSPSSTAFALMQTNDTKCLGYLKFVVQKFNGG
CcLDDS1    (249) DILHNETTTLLYSLEGMGMAGLDWEKLLKLKCKDGSFFSSPSSTAFALMETKDADCLNYLNNMVQKFNGG
CcLDDS2    (272) DLMHNVPTSLLYSLEGFAELELDWEKLLKLQSRGGSFLSSPASTALALMQTKDKNCLEYLNDVVQKFNGG
                 351                                                                 420
CcCLS      (333) APGQYPVEIFERIWVVDRLQRLGISRYFQLEIKECCLDYAFKHWT-QYGSSWARNTPVYDLDDTCMAFRI
CcLDDS1    (319) VPNTYPLDMFERIWVVDRLQRLGISRYFQKEVKD-CVDYVYRHWSSEHGICWAGNIDVYDTDDTAMGFRI
CcLDDS2    (342) APCQYPVEIFERIWVIDRLQRLGISRYFQLEIKD-CLDYVSKYWT-QYGCSWEKDSRMCDLDDTCMAFRI
                 421                                                                 490
CcCLS      (402) LRLHGYDVSAEAFRHFEKNGVFFCFGWETTQSVTVNFNLYRATQVAFPGENILKEAKQFSFNFLMKKQAA
CcLDDS1    (388) LRLHGYNVSAEVFNQFEKMGEFFSFPGQANESVTGIFNLYRAAQVMFPGEKILDKAKQFAFKFLCKKQAA
CcLDDS2    (410) LRLHGYHVSPEAFRHFENGGEFFCFPYQTTQSVTVNFNLYRATQVMFPGEKILEEAKQFSLNYLREKQAA
                 491                                                                 560
CcCLS      (472) REFQDKWVILKDFPGELKYALEFPWYASLPRVETRFYVEQYGGDNDVWIGKTLYRMPYINNNVYLELAKL
CcLDDS1    (458) NELLDKWIIMKDLPGEVGYGLKMPWYASLPRVESRFYIEQYGGGNDVWIGKTLYRMPYVNNNDYLELAKL
CcLDDS2    (480) NKFLDKWVILKDLPGEVEYALAVPWFASLPRVETRFYIEQYGGDNDVWIAKTFYRMGYVNNNVNLEVAKL
                 561                                                                 630
CcCLS      (542) DFNNCQALHRKEWETMQKWFMESKLDEFGVSSKTLLESYFLAAASIFEPERSTERLAWAKTAFLMETIGS
CcLDDS1    (528) DFNGCQALHRMEWNTMQKWFTENKFDDFGMSRRTLLVTYFVAASSIFEPERSWERLAWTKTAFMVEAIGS
CcLDDS2    (550) DFNNCQALHRIEWQAMQKWFVESKFCDFGASKNPLLIAYFLAASNIFEQERSVERLAWAKTTFLMEVIGS
                 631                                                                 700
CcCLS      (612) YFDDEMNSKDLRKAFVQEFKNIYERR---------MEAKGTKWNLIIILLTTLNHL----TEVCGRDINS
CcLDDS1    (598) YFENEMNSEDQRRAFVQEYKNMNLSP--SHISG--VSRRMTKRNLISVLETTLNHLSLDAIVAYGRDIST
CcLDDS2    (620) LFHDEVNSRDQRGAFIQEFKTINAKFGEGHIHGRKIEAKGLKNNLITILFTTLNQLSLEAMVACGRDINP
                 701                                                                 770
CcCLS      (669) YLCHSWEKWMMMWEPEGDRYKGAAELLSNSINLSSGRLFSNDTLSHPNYEKLVTLSNKLCHQLGN--SRR
CcLDDS1    (664) PLRHSWEKWMMMWEEEGDKYKGAAELIVKTINLSSG--QDNAILSHPKYQKLMNLANTICHQLGHYQTQK
CcLDDS2    (690) YLRHSWEKWMMMWEQEGDMYKGAAELLVNTVNLCSGRLFSNDTLSHPNYERLVTLSNKVCHQLGN--TLG
                 771                                                                 840
CcCLS      (737) GNHNEDSDIK-----DTKIEIAMQELVQLVHQNSSDDISMDLKQTFFAVVRSFYYAAHCDRGTINSHIVK
CcLDDS1    (732) GHDNNGSSMAGFLDGNVKVETDMQELVQLVLENTSDDGDKRLKETFFGVARSFYYAAYCDLGTINFHIAK
CcLDDS2    (758) GNHNEDSDIK-----DTKIEIAMQELVQLVHQNSSDDISTDLKQTFFAVVRSFYYAAHCDQGTINSHIVK
                 841
CcCLS      (802) VLFESVV-
CcLDDS1    (802) VLFERVD-
CcLDDS2    (823) VLFESVV-
```

Figure S10. Amino acid sequence alignment of the class II labdane-type diterpenoid synthases CcCLS (Falara et al., 2010), CcLDDS1, and CcLDDS2 characterized in *C. creticus* subsp. *creticus*. Sequence alignment was produced by the ClustalW algorithm, utilizing the AlignX program from the Vector NTI software package (Invitrogen). The conserved DxDD motif is highlighted in bold and underlined font (**D**:Asp: Aspartic Acid, **L**:Leu: Leucine, and **T**:Thr: Threonine). Color code reveals the degree of similarity between the protein sequences examined. Identical sections are highlighted in yellow and red font, conservative sections are highlighted in cyan and blue font, and similar sections are highlighted in green and black font. Finally, weakly similar and non-similar sections are shown in green and black font letters, respectively.

```
                  10        20        30        40        50        60        70        80        90       100
          ....|....|....|....|....|....|....|....|....|....|....|....|....|....|....|....|....|....|....|....|
CcLAT     MKLGKNRMKKMEISVITSETVN-FPSHASTQPFKLSLIDQMFPSSLYFPVILFFNPPTTPLDFAT---QLKKSLAQTLLSFYPIAGSTLDNVYIDRFDKG
RhAAT1    -------MEKIEVSIISRDTIKPSAASSSLHPYKLSIIDQFTP-TTYFPVIFFYPITDRVFNLPQTLTDLKNTVSQALTLYHPLSGRIKNNLYIDDFEAG
FvVAAT    -------MEKIEVSIISKHTIKPSTSSSPLQPYKLTLLDQLTP-PSYVPMVFFYPITGPAVFNLQTLADLRHALSETLTLYYPLSGRVKNNLYIDDFEEG
FaSAAT    -------MEKIEVSINSKHTIKPSTSSTPLQPYKLTLLDQLTP-PAYVPIVFFYPITDHDFNLPQTLADLRQALSETLTLYYPLSGRVKNNLYIDDFEEG

                 110       120       130       140       150       160       170       180       190       200
          ....|....|....|....|....|....|....|....|....|....|....|....|....|....|....|....|....|....|....|....|
CcLAT     VRFIETRVTNFNSLSDYLQELEIETLNLLFPCQPH--LEVTEPPKEQVLVQLNRFNCGGIALGICFSHKIIDGATGSLFLKNWAANNTEMHFPDGKKKNE
RhAAT1    IPYLEARVN--FHMIDFLRLPKIEWLNEFVPMAPYRKETIS-EFLPLLGIQVNIFDSG-IAIGVSFSHKINDGQTASCFLKSWVAIFRGY-------RNK
FvVAAT    VPYLEARVN--CDMNDFLRLPKIECLNEFVPIKPFSMEAISDERYPLLGVQVNIFNSG-IAIGVSVSHKLIDGRTSDCFLKSWCAVFRGS-------RDK
FaSAAT    VPYLEARVN--CDMTDFLRLRKIECLNEFVPIKPFSMEAISDERYPLLGVQVNVFDSG-IAIGVSVSHKLIDGGTADCFLKSWGAVFRGC-------REN

                 210       220       230       240       250       260       270       280       290       300
          ....|....|....|....|....|....|....|....|....|....|....|....|....|....|....|....|....|....|....|....|
CcLAT     IKHPDLSAGSQLFPPQESNSQDIPTFIVG---DHKKRKTKRFGFTSEAVEKLRLKCKGKVVKKPSRVEALTAFIWKSMVSASR---SVTTVVKPSVIVQV
RhAAT1    IIHPNLSQAALLLPSRDDLPEKYVAMMERMWFGEKKVVTRRFVFDAKAISALQDEGKSEYVPKPSRVQALTGFLWKHQLAASRALSSG-TSTRFSVASQT
FvVAAT    IIHPNLSQAALLFPPRDDLPEKYARQMEGLWFVGKKVATRRFVFGAKAISVIQDEAKSESVPKPSRVQAVTSFLWKHLIATSRALTSGTTSTRLSIATQV
FaSAAT    IIHPSLSEAALLFPPRDDLPEKYVDQMEALWFAGKKVATRRFVFGVKAISSIQDEAKSESVPKPSRVHAVTGFLWKHLIAASRALTSGTTSTRLSIAAQA

                 310       320       330       340       350       360       370       380       390       400
          ....|....|....|....|....|....|....|....|....|....|....|....|....|....|....|....|....|....|....|....|
CcLAT     VNLRSRTNPP-LPQNAIGSLIWRGAVTHDK-------EEMELSELVCMQREMLQNISENGFQVIKER--------MKMTQDMFSKSMDRVELYGFSSWLG
RhAAT1    VNLRSKMNMKTTLDNAIGNIFLWASARLDLNDTAPGSSDLKLCDLVNLLNESIKEFNSDYLEILKGKEGYGGMCDLLDFMEEGSFVEPAPEFYSFSSWTR
FvVAAT    VNIRSRRNMETVWDNAIGNLIWFAPAILELSHTTLEISDLKLCDLVNLLNGSVKQCNGDYFETFMGKEGYGSMCEYLDFQRTMSSMEPAPEIYLFTSWTN
FaSAAT    VNLRTRMNMETVLDNATGNLFWWAQAILELSHTTPEISDLKLCDLVNLLNGSVKQCNGDYFETFKGKEGYGRMCEYLDFQRTMSSMEPAPDIYLFSSWTN

                 410       420       430       440       450       460       470       480
          ....|....|....|....|....|....|....|....|....|....|....|....|....|....|....|....|.
CcLAT     FGFHEINFGWGKPVWIGIFGETGVPSTNLTILKELGGSTNSVEAWITFEEEKMSILENDPEFLAFAIPNPPIKLLD-----
RhAAT1    F-FDQVDFGWGRPSWVGFSGRVETR--NFTIFVETQCDDG-IDAWVTVDEKQMAMLEQDPQFLAFASPNPRISIASSVGMD
FvVAAT    F-FNQIDFGWGRTSWIGVAGKIESAFCNLTTLVPTPCDTG-IEAWVNLEEEKMAMLEQDPQFLALASPKTLISRY------
FaSAAT    F-FNPIDFGWGRTSWIGVAGKIESASCKFIILVPTQCGSG-IEAWVNLEEEKMAMLEQDPHFLALASPKTLI---------
```

Figure S11. Protein sequence alignment of BAHD acetyltransferases. *C. creticus* subsp. *creticus* CcLAT acetyltransferase (CcLAT – 448 amino acids) is aligned with *Rosa hybrida* (RhAAT1 – 457 amino acids – GenBank ID: AAW31948) (Shalit et al., 2003), *Fragaria vesca* (FvVAAT – 455 amino acids – GenBank ID: CAC09062) (Beekwilder et al., 2004), and *Fragaria* x *ananassa* (FaSAAT – 452 amino acids – GenBank ID: AAG13130) (Aharoni et al., 2000) BAHD acetyltransferases. Both HXXXD and DFGWG conserved sequence motifs of the BAHD superfamily of acetyltransferases are marked with red box (**H**:His: Histidine, **K**:Lys: Lysine, **I**:Ile: Isoleucine, **D**:Asp: Aspartic Acid, **N**:Asn: Asparagine, **L**:Leu: Leucine, **F**:Phe: Phenylalanine, **G**:Gly: Glycine, **W**:Trp: Tryptophan). Alignment was conducted with the Bioedit Software Program using the ClustalW algorithm.

Table S1. List of labdane-related diterpenoids (LRDs) identified in *n*-hexane extracts from various tissues of *C. creticus* subsp. *creticus* by GC-MS analysis. Each column depicts retention time (RT, min), name of LRD, and peak area (Area, AU: arbitrary units). Tissues used: leaf stages (1-4), stem, flower, blossom, fruit, and root. Samples were analyzed using nonadecane as an internal standard (n=3, ± SE).

	RT (min)	LRD	Leaf Stage 1		Leaf Stage 2		Leaf Stage 3		Leaf Stage 4		Stem		Flower		Blossom		Fruit		Root	
			**Area (AU)**	**± SE**	**Area (AU)**	**± SE**	**Area (AU)**	**± SE**	**Area (AU)**	**± SE**	**Area (AU)**	**± SE**	**Area (AU)**	**± SE**	**Area (AU)**	**± SE**	**Area (AU)**	**± SE**	**Area (AU)**	**± SE**
**1**	24.99	labda-7,13(16),14-triene	1.2E+08	4.0E+06	1.1E+08	7.6E+06	7.1E+07	2.6E+06	4.2E+07	2.9E+06	2.7E+07	9.5E+06	1.2E+07	7.3E+05	1.1E+07	1.1E+06	5.4E+06	7.3E+05	3.2E+05	9.5E+04
**2**	25.43	non-identified	8.3E+06	3.7E+05	7.6E+06	5.0E+05	6.8E+06	2.9E+05	5.7E+06	4.7E+05	1.0E+07	5.4E+06	2.5E+06	2.1E+05	2.2E+06	2.8E+05	5.7E+06	5.0E+05	0.0E+00	0.0E+00
**3**	25.65	*ent*-manoyl oxide	3.7E+07	1.6E+06	4.4E+07	3.2E+06	2.7E+07	1.5E+06	1.6E+07	1.4E+06	8.4E+06	3.1E+06	2.1E+06	4.4E+05	1.8E+06	2.3E+05	7.6E+05	9.6E+04	4.0E+05	1.6E+04
**4**	26.22	*ent*-13-*epi*-manoyl oxide	5.6E+07	2.0E+06	5.9E+07	5.5E+06	4.1E+07	2.3E+06	2.4E+07	2.7E+06	1.1E+07	5.2E+06	3.5E+06	1.6E+05	2.8E+06	4.9E+05	6.5E+06	2.4E+06	5.8E+04	1.0E+04
**5**	26.54	labda-7,12,14-triene	3.4E+07	2.4E+06	3.2E+07	1.4E+06	1.8E+07	2.0E+06	1.1E+07	5.8E+05	7.3E+06	1.9E+06	3.3E+06	1.6E+05	3.3E+06	1.6E+05	1.2E+06	9.8E+04	4.5E+04	1.7E+04
**6**	27.02	*ent*-kaurene	5.0E+06	5.9E+05	3.8E+06	3.1E+05	2.9E+06	1.8E+05	1.8E+06	1.8E+05	2.2E+06	1.4E+06	9.8E+05	4.6E+03	7.7E+05	1.2E+04	9.6E+05	6.3E+05	0.0E+00	0.0E+00
**7**	27.18	manool	1.1E+05	3.7E+04	6.9E+04	2.0E+02	1.7E+04	2.4E+02	1.7E+04	7.0E+03	9.4E+04	4.2E+04	2.3E+04	1.8E+02	3.8E+04	7.9E+02	2.3E+04	5.6E+02	0.0E+00	0.0E+00
**8**	28.00	labda-7,14-dien-13-ol	2.3E+07	1.2E+06	2.0E+07	6.0E+05	2.0E+07	1.4E+06	1.6E+07	1.3E+06	2.1E+07	1.3E+06	2.2E+07	3.8E+05	1.3E+07	1.4E+06	1.2E+07	1.4E+06	1.5E+07	3.0E+06
**9**	28.54	13(16),14-labdien-8-ol	1.7E+07	2.4E+06	1.8E+07	1.2E+06	1.1E+07	5.8E+05	7.9E+06	9.5E+05	5.2E+06	2.5E+06	3.3E+06	3.0E+05	4.9E+06	2.9E+05	3.8E+06	5.9E+05	1.3E+05	8.4E+03
**10**	29.66	neoabienol	2.9E+06	2.7E+05	2.7E+06	2.4E+04	1.6E+06	8.9E+03	1.0E+06	2.8E+04	1.0E+06	5.6E+05	1.9E+05	1.4E+04	2.1E+05	1.9E+03	2.2E+05	3.6E+03	0.0E+00	0.0E+00
**11**	30.46	*cis*-abienol	8.7E+06	8.9E+05	8.8E+06	4.7E+05	5.2E+06	1.2E+05	3.0E+06	2.1E+05	1.9E+06	7.6E+05	5.6E+05	2.8E+04	2.9E+05	2.9E+03	2.6E+05	8.4E+02	0.0E+00	0.0E+00
**12**	31.62	sclareol	1.8E+06	3.2E+05	1.7E+06	1.1E+05	1.4E+06	5.7E+04	1.4E+06	1.7E+05	1.1E+06	3.1E+05	4.3E+05	4.6E+03	2.6E+05	1.9E+03	1.6E+05	5.9E+02	0.0E+00	0.0E+00
**13**	31.96	*ent*-copalol	5.3E+06	7.8E+04	4.5E+06	9.7E+04	2.8E+06	8.7E+03	1.7E+06	1.6E+04	2.0E+06	5.8E+05	1.3E+06	1.3E+05	8.7E+05	7.8E+04	7.3E+05	1.5E+04	0.0E+00	0.0E+00
**14**	33.08	*ent*-3*β*-hydroxy-13-*epi*-manoyl oxide	6.9E+07	4.8E+06	5.8E+07	2.9E+06	4.8E+07	1.5E+06	3.1E+07	2.5E+06	1.8E+07	4.2E+06	7.5E+06	2.7E+05	5.8E+06	8.7E+05	6.6E+06	2.1E+06	1.7E+05	5.8E+04
**15**	33.36	labda-7,13(*E*)-dien-15-ol	2.1E+08	6.0E+06	2.0E+08	1.8E+07	1.1E+08	1.9E+06	8.1E+07	7.6E+06	9.8E+07	5.3E+07	5.4E+07	3.1E+06	4.9E+07	6.8E+06	1.9E+07	4.5E+06	4.2E+05	7.2E+04
**16**	35.74	*ent*-3*β*-acetoxy-13-*epi*-manoyl oxide	5.1E+07	4.9E+06	4.2E+07	3.5E+06	3.7E+07	2.3E+06	2.4E+07	4.4E+06	1.5E+07	1.2E+07	1.1E+06	4.7E+03	5.2E+05	2.3E+04	3.7E+06	1.8E+06	3.6E+05	1.2E+05
**17**	36.14	labda-7,13(*E*)-dien-15-yl acetate	4.9E+08	4.3E+07	5.0E+08	3.6E+07	3.6E+08	1.3E+07	2.5E+08	1.9E+07	1.6E+08	6.6E+07	1.1E+08	1.2E+07	8.0E+07	1.2E+07	2.1E+07	8.7E+06	7.7E+04	3.1E+02
**18**	36.52	labda-13(*E*)-ene-8*α*,15-diol	4.1E+07	1.2E+06	3.9E+07	3.6E+06	2.8E+07	1.3E+06	2.4E+07	2.2E+06	2.2E+07	1.1E+07	1.3E+07	5.4E+05	8.6E+06	9.2E+04	7.0E+06	8.0E+05	4.2E+05	1.1E+05
**19**	37.71	labda-13(*E*)-ene-8*α*-ol-15-yl acetate	3.9E+07	2.5E+06	3.4E+07	2.6E+06	2.4E+07	2.0E+06	1.8E+07	1.9E+06	2.0E+07	8.5E+06	5.2E+07	7.3E+06	1.8E+07	2.4E+06	9.5E+06	1.3E+06	0.0E+00	0.0E+00

Table S2. Retention index (RI) values calculated for each of the 19 peaks identified in this study compared with their corresponding RI values as cited in GC/MS libraries (FFNSC 2; Adams, 2007; massfinder 3) and the literature.

	**LRD**	**this study**	**FFNSC 2**	**Adams, 2007**	**mass finder 3**	Pichette et al., 1998	**Anastasaki et al., 1999**	**Demetzos et al., 2002**	**Skaltsa et al., 2003**	**Saroglou et al., 2006**	**Skorić et al., 2012**	**Kännaste et al., 2018**	**Skorić et al., 2022**
1	**labda-7,13(16),14-triene**	**1970**			1978						1967.8	1983	
2	**non-identified**	**1987**											
3	**manoyl oxide**	**1995**	1989	1987	2007		1989	1989		1990	1986.0	2009	1984
4	**13-*epi*-manoyl oxide**	**2016**	2022	2010	2023	2002	2010	2010	2012		2007.5	2030	2007
5	**labda-7,12,14-triene**	**2027**			2036						2024.8		
6	***ent*-kaurene**	**2045**	2045	2043	2056				2036				
7	**manool**	**2050**	2062	2057	2070			2056				2064	
8	**labda-7,14-dien-13-ol**	**2081**			2096						2082.6	2097	
9	**13(16),14-labdien-8-ol (isoabienol)**	**2100**			2124	2107			2101	2120		2121	
10	**neoabienol**	**2141**				2144							
11	***cis*-abienol**	**2170**	2152	2149	2146	2150							
12	**sclareol**	**2212**	2225	2223	2231						2217.9		2221
13	***ent*-copalol**	**2225**			2265								
14	**3β-hydroxy-13-*epi*-manoyl oxide**	**2266**					2268	2270	2239				
15	**labda-7,13(*E*)-dien-15-ol**	**2276**	2293	2292			2280	2286			2282.2		2291
16	**3β-acetoxy-13-*epi*-manoyl oxide**	**2372**					2376	2376	2370				2391
17	**labda-7,13(*E*)-dien-15-yl acetate**	**2388**		2392			2393				2392.3		2398
18	**labda-13(*E*)-ene-8α,15-diol**	**2408**	2420	2422			2409	2408	2409		2412.5		2427
19	**labda-13(*E*)-ene-8α-ol-15-yl acetate**	**2499**					2505	2505					

Table S3. List of known plant diterpenoid synthases (diTPSs) that were used for the phylogenetic analysis of CcLDDS1 and CcLDDS2. The columns depict name, product(s), class of monofunctional (class I, II) and/or bifunctional (class I/II) diTPSs, motif position, sequence length (amino acids), mono-, bi-functional type, substrate(s), plant species, plant group, NCBI Accession Number and references of 134 members of the terpene synthases subfamilies TPS-b, TPS-c, TPS-d, TPS-e/f, and TPS-h. *C. creticus* subsp. *creticus* labda-7,13(*E*)-dien-15-yl diphosphate synthases CcLDDS1 & CcLDDS2, functionally characterized in this study, are highlighted in purple font color.

No	Name	Product (-s)	Class (I, II & I/II)	Motif (position)	Length	Monofunctional / Bifunctional	Substrate(s)	Plant species	Plant Group	NCBI Accession Number	Reference(s)
**TPS-b**											
1	TrTPS8	miltiradiene<br>*ent*-manool<br>*syn*-manool	I	DDIHD (302-306)	537	M	(+)-CPP<br>*ent*-CPP<br>*syn*-CPP	*Tripterygium regelii*	AD	ASP45391	Inabuy et al., 2017
2	TwTPS27	miltiradiene<br>(13*S*)-*ent*-manoyl oxide	I	DDIHD (342-346)	589	M	(+)-CPP<br>*ent*-8-OH-CPP	*Tripterygium wilfordii*	AD	ANO43011	Hansen et al., 2017
**TPS-c**											
1	AtCPS	*ent*-CPP	II	DIDD (377-380)	802	M	GGPP	*Arabidopsis thaliana*	AD	AAA53632	Sun and Kamiya, 1994
2	CcCLS	8α-OH-CPP (copal-8-ol diphosphate)	II	DLDD (391-394)	808	M	GGPP	*Cistus creticus* subsp. *creticus*	AD	ADJ93862	Falara et al., 2010
**3**	**CcLDDS1**	***endo*-7,13-CPP**	**II**	**DTDD (377-380)**	**808**	**M**	**GGPP**	***Cistus creticus* subsp. *creticus***	**AD**	**MT666220**	**This study**
**4**	**CcLDDS2**	***endo*-7,13-CPP**	**II**	**DLDD (399-402)**	**829**	**M**	**GGPP**	***Cistus creticus* subsp. *creticus***	**AD**	**MT666221**	**This study**
5	CfTPS1	(+)-CPP	II	DIDD (376-379)	786	M	GGPP	*Coleus forskohlii* [syn. *Plectranthus barbatus*]	AD	AHW04046	Pateraki et al., 2014
6	CfTPS2	8α-OH-CPP	II	DIDD (358-361)	773	M	GGPP	*Coleus forskohlii*	AD	AHW04047	Pateraki et al., 2014
7	CmCPS1	*ent*-CPP	II	DIDD (382-385)	823	M	GGPP	*Cucurbita maxima*	AD	AAD04292	Smith et al., 1998
8	CmCPS2	*ent*-CPP	II	DIDD (388-391)	827	M	GGPP	*Cucurbita maxima*	AD	AAD04293	Smith et al., 1998
9	EpTPS7	*ent*-CPP	II	DVDD (387-390)	807	M	GGPP	*Euphorbia peplus*	AD	AGN70883	Zerbe et al., 2013
10	GrTPS1	8α-OH-CPP	II	DLDD (355-358)	765	M	GGPP	*Grindelia robusta*	AD	AGN70887	Zerbe et al., 2013
11	GrTPS2	*endo*-7,13-CPP	II	DIDD (323-326)	733	M	GGPP	*Grindelia robusta*	AD	AKP96361	Zerbe et al., 2015
12	HaCPS1L	*ent*-CPP	II	DIDD (374-377)	798	M	GGPP	*Helianthus annuus*	AD	CBL42915	Pugliesi et al., 2011
13	HsTPS1	*endo*-7,13-CPP	II	DLDD (366-369)	776	M	GGPP	*Mesosphaerum suaveolens*	AD	AZB50380	Johnson et al., 2019
14	HvCPS1	*ent*-CPP	II	DVDD (378-381)	826	M	GGPP	*Hordeum vulgare* subsp. *vulgare*	AM	AAT49065	Spielmeyer et al., 2004
15	IeCPS1	*ent*-CPP	II	DIDD (277-280)	710	M	GGPP	*Isodon eriocalyx*	AD	AEP03177	Li et al., 2012
16	IeCPS2	*ent*-CPP	II	DVDD (369-372)	794	M	GGPP	*Isodon eriocalyx*	AD	AEP03175	Li et al., 2012

No	Name	Product (-s)	Class (I, II & I/II)	Motif (position)	Length	Monofunctional / Bifunctional	Substrate(s)	Plant species	Plant Group	NCBI Accession Number	Reference(s)
**TPS-c** (continued…)											
17	IlCPS2	*endo*-7,13-CPP	II	DLDD (372-375)	794	M	GGPP	*Isodon lophanthoides* var. *gerardianus*	AD	QXT24222	Yang et al., 2021
18	IrCPS1	(+)-CPP	II	DIDD (381-384)	796	M	GGPP	*Isodon rubescens*	AD	APJ36371	Jin et al., 2017
19	IrCPS4	*ent*-CPP	II	DVDD (371-374)	783	M	GGPP	*Isodon rubescens*	AD	APJ36374	Jin et al., 2017
20	IrTPS3	(+)-CPP	II	DIDD (383-386)	798	M	GGPP	*Isodon rubescens*	AD	ARO38141	Pelot et al., 2017a
21	IrTPS5	*ent*-CPP	II	DVDD (371-374)	796	M	GGPP	*Isodon rubescens*	AD	ARO38143	Pelot et al., 2017a
22	LsCPS1	*ent*-CPP	II	DIDD (376-379)	799	M	GGPP	*Lactuca sativa*	AD	BAB12440	Sawada et al., 2008
23	MvCPS1	9-OH-CPP	II	DIDD (371-374)	776	M	GGPP	*Marrubium vulgare*	AD	AIE77090	Zerbe et al., 2014
24	MvCPS3	(+)-CPP	II	DIDD (371-374)	785	M	GGPP	*Marrubium vulgare*	AD	AIE77092	Zerbe et al., 2014
25	NtCPS2	8α-OH-CPP	II	DVDD (382-385)	802	M	GGPP	*Nicotiana tabacum*	AD	CCD33018	Sallaud et al., 2012
26	OsCPS1	*ent*-CPP	II	EVDD (418-421)	867	M	GGPP	*Oryza sativa*	AM	Q6ET36	Prisic et al., 2004
27	OsCPS2	*ent*-CPP	II	DIDD (374-377)	800	M	GGPP	*Oryza sativa*	AM	Q6Z5I0	Prisic et al., 2004
28	OsCPS4	*syn*-CPP	II	DIDD (365-368)	767	M	GGPP	*Oryza sativa*	AM	Q6E7D7	Xu et al., 2004
29	PgCPS	*ent*-CPP	II	DVDD (326-329)	761	M	GGPP	*Picea glauca*	G	ADB55707	Keeling et al., 2010
30	PsCPS	*ent*-CPP	II	DVDD (326-329)	761	M	GGPP	*Picea sitchensis*	G	ADB55709	Keeling et al., 2010
31	PtTPS17	*ent*-CPP	II	DIDD (359-362)	795	M	GGPP	*Populus trichocarpa*	AD	ALM22923	Irmisch et al., 2015
32	PvCPS1	*ent-neo-cis-trans*-KPP	II	DVDD (366-369)	796	M	GGPP	*Panicum virgatum*	AM	AXK78845	Pelot et al., 2018
33	PvCPS3	(+)-*endo*-8,13-CPP	II	DIDD (339-342)	772	M	GGPP	*Panicum virgatum*	AM	AXK78846	Pelot et al., 2018
34	PvCPS8	*syn*-CPP	II	DIDD (357-360)	756	M	GGPP	*Panicum virgatum*	AM	AXK78847	Pelot et al., 2018
35	PvCPS11	*ent*-8α-OH-CPP<br>*ent*-8β-OH-CPP	II	DVDD (380-383)	828	M	GGPP	*Panicum virgatum*	AM	AXK78848	Pelot et al., 2018
36	PvCPS14	*ent*-CPP	II	DVDD (393-396)	837	M	GGPP	*Panicum virgatum*	AM	AXK78849	Pelot et al., 2018
37	PvCPS15	*ent*-CPP	II	DVDD (404-407)	848	M	GGPP	*Panicum virgatum*	AM	AXK78850	Pelot et al., 2018
38	RoCPS1	(+)-CPP	II	DIDD (375-378)	799	M	GGPP	*Rosmarinus officinalis*	AD	AHL67261	Brückner et al., 2014
39	SdCPS1	*ent*-CPP	II	DIDD (373-376)	789	M	GGPP	*Salvia divinorum*	AD	APH81399	Pelot et al., 2017
40	SdCPS2	KPP	II	DSDD (368-371)	788	M	GGPP	*Salvia divinorum*	AD	APH81400	Pelot et al., 2017
41	SdKPS	KPP	II	DSDD (368-371)	787	M	GGPP	*Salvia divinorum*	AD	AOZ15895	Chen et al., 2017
42	SfCPS	(+)-CPP	II	DIDD (372-375)	796	M	GGPP	*Salvia fruticosa*	AD	AJQ30184	Božić et al., 2015
43	SmCPS	(+)-CPP	II	DIDD (370-373)	793	M	GGPP	*Salvia miltiorrhiza*	AD	ABV57835	Gao et al., 2009
44	SmCPS1	(+)-CPP	II	DIDD (370-373)	793	M	GGPP	*Salvia miltiorrhiza* f. alba	AD	AHJ59321	Cui et al., 2015
45	SmCPS2	(+)-CPP	II	DIDD (373-376)	798	M	GGPP	*Salvia miltiorrhiza* f. alba	AD	AHJ59322	Cui et al., 2015

Table S3 (continued…)

No	Name	Product (-s)	Class (I, II & I/II)	Motif (position)	Length	Monofunctional / Bifunctional	Substrate(s)	Plant species	Plant Group	NCBI Accession Number	Reference(s)
**TPS-c** (continued…)											
46	SmCPS4	*ent*-8*β*-OH-CPP	II	DSDD (357-360)	776	M	GGPP	*Salvia miltiorrhiza* f. alba	AD	AKN91186	Cui et al., 2015
47	SmCPS5	*ent*-CPP	II	DIDD (372-375)	793	M	GGPP	*Salvia miltiorrhiza* f. alba	AD	AHJ59324	Cui et al., 2015
48	SrCPS	*ent*-CPP	II	DIDD (373-376)	787	M	GGPP	*Stevia rebaudiana*	AD	AAB87091	Richman et al., 1999
49	SrCPS2	8*α*-OH-CPP	II	DIDD (365-368)	777	M	GGPP	*Stevia rebaudiana*	AD	ALJ30096	Kim et al., 2015
50	SsLPPS	8*α*-OH-CPP	II	DIDD (372-375)	785	M	GGPP	*Salvia sclarea*	AD	AFU61897	Caniard et al., 2012
51	SsLPS	8*α*-OH-CPP	II	DIDD (372-375)	784	M	GGPP	*Salvia sclarea*	AD	AET21248	Schalk et al., 2012
52	TaCPS1	*ent*-CPP		DIDD (371-374)	797	M	GGPP	*Triticum aestivum*	AM	BAH56558	Toyomasu et al., 2009; Wu et al., 2012
53	TaCPS2	(+)-CPP	II	DIDD (360-363)	757	M	GGPP	*Triticum aestivum*	AM	BAH56559	Toyomasu et al., 2009; Wu et al., 2012
54	TaCPS3	*ent*-CPP	II	DVDD (384-387)	831	M	GGPP	*Triticum aestivum*	AM	BAH56560	Toyomasu et al., 2009; Wu et al., 2012
55	TaCPS4	*ent*-CPP	II	DVDD (335-338)	782	M	GGPP	*Triticum aestivum*	AM	BAP01383	Wu et al., 2012
56	TrTPS1	*ent*-CPP	II	DIDD (382-385)	816	M	GGPP	*Tripterygium regelii*	AD	ASP43411	Inabuy et al., 2017
57	TrTPS2	(+)-CPP	II	DVDD (385-388)	807	M	GGPP	*Tripterygium regelii*	AD	ASP43410	Inabuy et al., 2017
58	TwTPS3	*ent*-CPP	II	DIDD (382-385)	816	M	GGPP	*Tripterygium wilfordii*	AD	ANO43020	Hansen et al., 2017
59	TwTPS7	(+)-CPP	II	DVDD (385-388)	807	M	GGPP	*Tripterygium wilfordii*	AD	ALE19955	Andersen-Ranberg et al., 2016
60	TwTPS9	(+)-CPP	II	DVDD (385-388)	807	M	GGPP	*Tripterygium wilfordii*	AD	ANO43022	Hansen et al., 2017
61	TwTPS10	KPP	II	DIDD (379-382)	815	M	GGPP	*Tripterygium wilfordii*	AD	ANO43021	Hansen et al., 2017
62	TwTPS14	KPP	II	DIDD (379-382)	815	M	GGPP	*Tripterygium wilfordii*	AD	ALE19956	Andersen-Ranberg et al., 2016
63	TwTPS21	*ent*-8-OH-CPP	II	DIDD (379-382)	815	M	GGPP	*Tripterygium wilfordii*	AD	ALE19957	Andersen-Ranberg et al., 2016
64	ZmAN1	*ent*-CPP	II	DVDD (378-381)	823	M	GGPP	*Zea mays*	AM	AAA73960	Bensen et al., 1995
65	ZmAN2	*ent*-CPP	II	EVDD (374-377)	827	M	GGPP	*Zea mays*	AM	AAT70084	Harris et al., 2005
66	ZmCPS3	(+)-CPP	II	DVDD (363-366)	785	M	GGPP	*Zea mays*	AM	Zm00001d0 24512	Murphy et al., 2018
67	ZmCPS4	*endo*-8,13-CPP	II	DIDD (318-321)	778	M	GGPP	*Zea mays*	AM	Zm00001d0 48874	Murphy et al., 2018
**TPS-d**											
1	AgAs	abietadiene	I/II	DIDD (402-405) & DDLYD (621-625)	868	B	GGPP	*Abies grandis*	G	AAB05407	Stoffer-Vogel et al., 1996
2	AbCAS	*cis*-abienol	I/II	DIDD (403-406) & DDLYD (622-626)	867	B	GGPP	*Abies balsamea*	G	AEL99953	Zerbe et al., 2012

Table S3 (continued…)

No	Name	Product (-s)	Class (I, II & I/II)	Motif (position)	Length	Monofunctional / Bifunctional	Substrate(s)	Plant species	Plant Group	NCBI Accession Number	Reference(s)
						**TPS-d** (continued…)					
3	GbLPS	levopimaradiene	I/II	DVDD (405-408) & DDLYD (624-628)	873	B	GGPP	*Ginkgo biloba*	G	AAL09965	Schepmann et al., 2001
4	PaLAS	levopimaradiene /abietadiene	I/II	DIDD (392-395) & DDLYD (611-615)	859	B	GGPP	*Picea abies*	G	AAS47691	Martin et al., 2004
5	Palso	isopimaradiene	I/II	DIDD (400-403) & DDLYD (619-623)	867	B	GGPP	*Picea abies*	G	AAS47690	Martin et al., 2004
6	TbTS	taxadiene	I	DDMAD (613-617)	862	M	GGPP	*Taxus brevifolia*	G	AAC49310	Wildung and Croteau, 1996
						**TPS-e/f**					
1	AtKS	*ent*-kaurene	I	DDFFD (531-535)	785	M	*ent*-CPP	*Arabidopsis thaliana*	AD	AAC39443	Yamaguchi et al., 1998
2	CfTPS3	(13*R*)-manoyl oxide	I	DDFFD (330-334)	598	M	8α-OH-CPP	*Coleus forskohlii*	AD	AHW04048	Pateraki et al., 2014
		miltiradiene					(+)-CPP				
3	CfTPS4	miltiradiene	I	DDFFD (331-335)	587	M	8α-OH-CPP	*Coleus forskohlii*	AD	AHW04049	Pateraki et al., 2014
		(13*R*) & (13*S*)-manoyl oxide					(+)-CPP				
4	CfTPS14	*ent*-kaurene	I	DDFFD (543-547)	792	M	*ent*-CPP	*Coleus forskohlii*	AD	AGN70881	Zerbe et al., 2013
5	CmKS	*ent*-kaurene	I	DDFYD (536-540)	789	M	*ent*-CPP	*Cucurbita maxima*	AD	AAB39482	Yamaguchi et al., 1996
6	EpTPS1	*ent*-kaurene	I	DDFFD (530-534)	783	M	*ent*-CPP	*Euphorbia peplus*	AD	AGN70882	Zerbe et al., 2013
7	EpTPS8	*ent*-sandaracopimaradiene	I	DDFFD (540-544)	792	M	*ent*-CPP	*Euphorbia peplus*	AD	ALE19952	Andersen-Ranberg et al., 2016
		(+)-sandaracopimaradiene					(+)-CPP				
8	EpTPS23	*ent*-sandaracopimaradiene	I	DDFFE (533-537)	782	M	*ent*-CPP	*Euphorbia peplus*	AD	ALE19953	Andersen-Ranberg et al., 2016
		(+)-sandaracopimaradiene					(+)-CPP				
9	GrTPS4	*ent*-kaurene	I	DDFFD (535-539)	795	M	*ent*-CPP	*Grindelia robusta*	AD	AKP96362	Zerbe et al., 2015
10	GrTPS6	manoyl oxide	I	DDFFD (557-561)	818	M	8α-OH-CPP	*Grindelia robusta*	AD	AGN70886	Zerbe et al., 2013
		pimaradiene- & abietadiene-type					*ent*-CPP				
		pimaradiene- & abietadiene-type					(+)-CPP				
11	HaKS2L	*ent*-kaurene	I	DDFID (528-532)	774	M	*ent*-CPP	*Helianthus annuus*	AD	CBL42916	Pugliesi et al., 2011
12	HaKS3L	*ent*-kaurene	I	DDFFD (525-529)	772	M	*ent*-CPP	*Helianthus annuus*	AD	CBL42917	Pugliesi et al., 2011
13	IrKSL1	miltiradiene	I	DDFFD (329-333)	597	M	(+)-CPP	*Isodon rubescens*	AD	APJ36376	Jin et al.,2017
14	IrKSL3	miltiradiene	I	DDVFD (529-533)	776	M	(+)-CPP	*Isodon rubescens*	AD	APJ36378	Jin et al.,2017
15	IrKSL4	*ent*-atiserene	I	DDLFD (556-560)	806	M	*ent*-CPP	*Isodon rubescens*	AD	ASC55316	Jin et al.,2017
16	IrKSL5	ent-kaurene	I	DDFFD (543-547)	794	M	ent-CPP	Isodon rubescens	AD	ASC55317	Jin et al.,2017

Table S3 (continued…)

No	Name	Product (-s)	Class (I, II & I/II)	Motif (position)	Length	Monofunctional / Bifunctional	Substrate(s)	Plant species	Plant Group	NCBI Accession Number	Reference(s)
**TPS-e/f** (continued…)											
17	IrKSL6	isopimaradiene	I	DDFFD (525-529)	775	M	(+)-CPP	*Isodon rubescens*	AD	ASC55318	Jin et al.,2017
18	IrTPS2	nezukol	I	DDVFD (530-534)	777	M	(+)-CPP	*Isodon rubescens*	AD	ARO38140	Pelot et al., 2017a
19	IrTPS4	miltiradiene	I	DDFFD (329-333)	626	M	(+)-CPP	*Isodon rubescens*	AD	ARO38142	Pelot et al., 2017a
20	LsKS1	*ent*-kaurene	I	DDFFD (537-541)	788	M	*ent*-CPP	*Lactuca sativa*	AD	BAB12441	Sawada et al., 2008
21	MvELS	9,13-epoxy-labda-14-ene	I	DDFFD (322-326)	580	M	9-OH-CPP	*Marrubium vulgare*	AD	AIE77094	Zerbe et al., 2014
		manoyl oxide					8*α*-OH-CPP				
		miltiradiene					(+)-CPP				
22	NtABS	*cis*-abienol	I	DDFFD (539-543)	792	M	8-OH-CPP	*Nicotiana tabacum*	AD	CCD33019	Sallaud et al., 2012
23	OsKS1	*ent*-kaurene	I	DDFFD (496-500)	756	M	*ent*-CPP	*Oryza sativa* Japonica Group	AM	AAQ72559	Margis-Pinheiro et al., 2005
24	OsKSL4	*syn*-pimara-7,15-diene	I	DDFFD (589-593)	840	M	*syn*-CPP	*Oryza sativa* Indica Group	AM	AAU05906	Wilderman et al., 2004
25	OsKSL8 (OsDTC2)	*syn*-stemar-13-ene	I	DDLFD (551-555)	820	M	syn-CPP	*Oryza sativa* Japonica Group	AM	BAD34478	Nemoto et al., 2004; Xu et al., 2007
26	OsKSL10	*ent*-(sandaraco)pimara-8(14),15-diene	I	DDFFD (550-554)	815	M	*ent*-CPP	*Oryza sativa* Japonica Group	AM	Q2QQJ5.2	Xu et al., 2007
27	PgKS	*ent*-kaurene	I	DDFFD (513-517)	757	M	*ent*-CPP	*Picea glauca*	G	ADB55708	Keeling et al., 2010
28	PsKS	*ent*-kaurene	I	DDFFD (513-517)	757	M	*ent*-CPP	*Picea sitchensis*	G	ADB55710	Keeling et al., 2010
29	PtTPS19	*ent*-kaurene	I	DDFFD (531-535)	782	M	*ent*-CPP	*Populus trichocarpa*	AD	ALM22925	Irmisch et al., 2015
30	PvKSL2	*syn*-pimara-9(11),15-diene	I	DDLFD (573-577)	841	M	*syn*-CPP	*Panicum virgatum*	AM	AXK78851	Pelot et al., 2018
		ent-neo-cis-trans-kolavelool					*ent-neo-cis-trans*-KPP				
		ent-sandaracopimaradiene					*ent*-CPP				
		(13*R*)-*ent*-manoyl oxide					*ent*-8*α*/*β*-OH-CPP				
31	PvKSL3	9*β*-hydroxy-*syn*-pimara-15-ene	I	DDLFD (408-412)	673	M	*syn*-CPP	*Panicum virgatum*	AM	AXK78852	Pelot et al., 2018
		ent-sandaracopimaradiene					*ent*-CPP				
		(13*R*)-*ent*-manoyl oxide					*ent*-8*α*/*β*-OH-CPP				
32	PvKSL8	9*β*-hydroxy-*syn*-pimar-15-ene	I	DDLFD (569-573)	836	M	*syn*-CPP	*Panicum virgatum*	AM	AXK78855	Pelot et al., 2018
		ent-sandaracopimaradiene					*ent*-CPP				
		(13*R*)-*ent*-manoyl oxide					*ent*- 8*α*/*β*-OH-CPP				
33	PvKSL13	*ent*-kaurene	I	DDFFD (546-550)	807	M	*ent*-CPP	*Panicum virgatum*	AM	AXK78856	Pelot et al., 2018
34	PvKSL15	*ent*-kaurene	I	DDFFD (547-551)	808	M	*ent*-CPP	*Panicum virgatum*	AM	AXK78857	Pelot et al., 2018

Table S3 (continued…)

No	Name	Product (-s)	Class (I, II & I/II)	Motif (position)	Length	Monofunctional / Bifunctional	Substrate(s)	Plant species	Plant Group	NCBI Accession Number	Reference(s)
						**TPS-e/f** (continued…)					
35	RoKSL1	miltiradiene	I	DDFFD (331-335)	592	M	(+)-CPP	*Salvia rosmarinus (Rosmarinus officinalis)*	AD	AHL67262	Brückner et al., 2014
		manoyl oxide					8-OH-CPP				
36	RoKSL2	miltiradiene	I	DDLFD (333-337)	590	M	(+)-CPP	*Salvia rosmarinus*	AD	AHL67263	Brückner et al., 2014
		manoyl oxide					8-OH-CPP				
37	SdKS	*ent*-kaurene	I	DDFFD (542-546)	791	M	*ent*-CPP	*Scoparia dulcis*	AD	AEF33360	Yamamura et al., 2018
38	SdKSL1	*ent*-pimaradiene	I	DDFFD (331-335)	590	M	*ent*-CPP	*Salvia divinorum*	AD	APG42602	Pelot et al., 2017
39	SfKSL	miltiradiene	I	DDLFD (334-338)	584	M	(+)-CPP	*Salvia fruticosa*	AD	AJQ30185	Božić et al., 2015
40	SlTPS24	*ent*-kaurene	I	DDFFD (559-563)	820	M	*ent*-CPP	*Solanum lycopersicum*	AD	AEP82778	Falara et al., 2011
41	SmKSL	miltiradiene	I	DDFFD (334-338)	595	M	(+)-CPP	*Salvia miltiorrhiza*	AD	ABV08817	Gao et al., 2009; Cui et al., 2015
42	SmKSL2	(13*S*)-*ent*-manoyl oxide	I	DDFFD (546-550)	807	M	*ent*-8*β*-OH-CPP	*Salvia miltiorrhiza* f. alba	AD	AHJ59325	Cui et al., 2015
		ent-kaurene					ent-CPP				
43	SrKS	*ent*-kaurene	I	DDFFD (528-532)	784	M	*ent*-CPP	*Stevia rebaudiana*	AD	AAD34294	Richman et al., 1999
		(13*R*)-manoyl oxide & (13*S*)-manoyl oxide					*ent*-8*β*-OH-CPP				Kim et al., 2015
44	SrKSL	(13*R*)-manoyl oxide	I	DDFFD (536-540)	782	M	*ent*-8*β*-OH-CPP	*Stevia rebaudiana*	AD	ALJ30097	Kim et al., 2015
		(13*S*)-manoyl oxide									
		unknown product					*ent*-CPP				
45	SsSS(1)	sclareol	I	DDFFD (329-333)	575	M	8-OH-CPP	*Salvia sclarea*	AD	AFU61898	Caniard et al., 2012
46	SsSS(2)	sclareol	I	DDFFD (329-333)	575	M	8-OH-CPP	*Salvia sclarea*	AD	AET21246	Schalk et al., 2012
47	SpMilS	miltiradiene	I	DDLFD (334-338)	584	M	(+)-CPP	*Salvia pomifera*	AD	AJA38249	Ignea et al., 2015b; Trikka et al., 2015b
48	TaKSL6	*ent*-kaurene	I	DDFFD (602-606)	852	M	*ent*-CPP	*Triticum aestivum*	AM	BAL41693	Zhou et al., 2012
49	TrTPS13	*ent*-kaurene	I	DDFFD (530-534)	779	M	*ent*-CPP	*Tripterygium regelii*	AD	ASP43412	Inabuy et al., 2017
		sandaracopimaradiene & isopimaradiene					(+)-CPP				
		syn-pimara-7,15-diene & *syn*-stemod-13(17)-ene					*syn*-CPP				
		ent-kaurene					*TrTPS2* product				
50	TrTPS14	*ent*-kaurene	I	DDFFD (531-535)	780	M	*ent*-CPP	*Tripterygium regelii*	AD	ASP43413	Inabuy et al., 2017

No	Name	Product (-s)	Class (I, II & I/II)	Motif (position)	Length	Monofunctional / Bifunctional	Substrate(s)	Plant species	Plant Group	NCBI Accession Number	Reference(s)
**Table S3** (continued…)											
**TPS-e/f** (continued…)											
51	TrTPS15	(+)-manool<br>*ent*-manool<br>*syn*-manool	I	DDFFD (468-472)	717	M	(+)-CPP<br>*ent*-CPP<br>*syn*-CPP	*Tripterygium regelii*	AD	ASP43414	Inabuy et al., 2017
52	TwTPS2	*ent*-kaur-16-ene<br>(13*R*)-*ent*-manoyl oxide	I	DDFFD (484-488)	733	M	*ent*-CPP<br>*ent*-8-OH-CPP	*Tripterygium wilfordii*	AD	ALE19954	Andersen-Ranberg et al., 2016
53	TwTPS16	*ent*-kaur-16-ene<br>(13*R*)-*ent*-manoyl oxide	I	DDFFD (531-535)	780	M	*ent*-CPP<br>*ent*-8-OH-CPP	*Tripterygium wilfordii*	AD	ANO43010	Hansen et al., 2017
54	TwTPS17	*ent*-kaur-16-ene<br>(13*R*)-*ent*-manoyl oxide	I	DDFFD (532-536)	781	M	*ent*-CPP<br>*ent*-8-OH-CPP	*Tripterygium wilfordii*	AD	ANO43008	Hansen et al., 2017
55	TwTPS18	*ent*-kaur-16-ene<br>(13*R*)-*ent*-manoyl oxide	I	DDFFD (532-536)	781	M	*ent*-CPP<br>*ent*-8-OH-CPP	*Tripterygium wilfordii*	AD	ANO43009	Hansen et al., 2017
**TPS-h**											
1	SmCPSKSL1	labda-7,13(*E*)-dien-15-ol	I/II	DIDD (284-287) / DDLAD (501-505)	750	B	GGPP	*Selaginella moellendorffii*	L	AEK75338	Mafu et al., 2011
2	SmMDS	miltiradiene	I/II	DIDD (389-392) / DDLMD (611-615)	867	B	GGPP	*Selaginella moellendorffii*	L	BAL41682	Sugai et al., 2011
**Tree Root**											
1	JsCPS/KS	*ent*-kaurene	I/II	DLDD (423-426) / DDYFD (642-646)	886	B	GGPP	*Jungermannia subulata*	Li	BAJ39816	Kawaide et al., 2011
2	PpCPS/KS	*ent*-kaurene<br>*ent*-16α-hydroxy kaurane	I/II	DVDD (417-420) / DDYFD (635-639)	881	B	GGPP	*Physcomitrella patens*	B	BAF61135	Hayashi et al., 2006

GGPP: geranylgeranyl diphosphate, **CPP:** copalyl diphosphate, ***endo*-7,13-CPP:** labda-7,13(*E*)-dien-15-yl diphosphate, **8-OH-CPP:** 8-hydroxy-copalyl diphosphate (copal-8-ol diphosphate), **9-OH-CPP:** 9-hydroxy-copalyl diphosphate (peregrinol diphosphate), ***endo*-8,13-CPP:** labda-8,13(*E*)-dien-15-yl diphosphate, **(13*R*)-manoyl oxide:** (13-*epi*)-manoyl oxide, **KPP:** clerodienyl diphosphate, **AD:** Angiosperm Dicotyledon, **AM:** Angiosperm Monocotyledon, **G:** Gymnosperm, **L:** Lycophyte, **Li:** Liverwort, and **B:** Bryophyte.

Table S4. RNA-seq data analysis of *C. creticus* subsp. *creticus* trichomes highlighting translated contigs sharing strong sequence similarity to known class II diterpenoid synthases (diTPSs).

No	Contig information		BLAST suite results (NCBI database)	Translated contig sequence analysis
	Contig No	Contig Length (nt)	diTPS of highest sequence similarity – top hit result	Motif
**1***	**68**	**2824**	**CcCLS**	**DTDD**
2	254	501	CcCLS	N/A
**3***	**404**	**905**	**CcCLS**	**DLDD**
**4***	**541**	**1075**	**CcCLS**	**DLDD**
5	634	185	CcCLS	N/A
6	647	520	CcCLS	N/A
7	764	105	CcCLS	N/A
8	815	124	CcCLS	N/A
9	936	686	CcCLS	N/A
10	1315	499	CcCLS	N/A
**11***	**1500**	**182**	**CcCLS**	N/A
**12***	**1782**	**318**	**CcCLS**	N/A
13	1868	294	CcCLS	N/A
14	1904	131	CcCLS	N/A
15	2021	144	CcCLS	N/A
16	2753	186	CcCLS	N/A
17	2901	351	CcCLS	N/A
**18***	**3201**	**113**	**PREDICTED: *Durio zibethinus ent*-copalyl diphosphate synthase, (LOC111306089)**	**DIDD**
19	3268	147	CcCLS	N/A
20	4331	243	PREDICTED: *Ziziphus jujuba ent*-copalyl diphosphate synthase, (LOC107426403)	N/A
21	4582	176	CcCLS	N/A
22	5100	122	CcCLS	N/A
**23***	**6200**	**103**	**CcCLS**	N/A
24	8033	230	CcCLS	N/A
25	9061	112	CcCLS	N/A
26	9169	106	CcCLS	N/A
**27***	**12960**	**773**	**CcCLS**	**DGDD**
28	13087	192	CcCLS	N/A
29	15043	222	CcCLS	N/A
30	18827	147	CcCLS	N/A
31	22435	351	CcCLS	N/A

Table S4 (continued…)				
**No**	**Contig information**		**BLAST suite results (NCBI database)**	**Translated contig sequence analysis**
	**Contig No**	**Contig Length (nt)**	**diTPS of highest sequence similarity – top hit result**	**Motif**
32	24316	451	CcCLS	N/A
33	30811	278	CcCLS	N/A
**34***	**31893**	**214**	**CcCLS**	**DADD**
35	37398	310	CcCLS	N/A
36	39494	102	CcCLS	N/A
37	41015	282	PREDICTED: *Citrus sinensis ent*-copalyl diphosphate synthase, (LOC102620281)	N/A
38	52078	248	CcCLS	N/A
39	64685	160	PREDICTED: *Ricinus communis ent*-copalyl diphosphate (LOC8272032)	N/A
40	78280	113	CcCLS	N/A
41	86020	177	CcCLS	N/A
42	95029	104	CcCLS	N/A
43	11702	109	CcCLS	N/A

*** contigs containing the full length nucleotide sequence of a candidate diTPS gene**

*** contigs containing a partial sequence of the *CcCLS* ORF sequence**

*** translated contig sequences containing the DxDD motif**

N/A: translated contig sequences not containing any motif

Table S5. List of BAHD acetyltransferases (Clades I-V) according to D'Auria (2006) used for *C. creticus* subsp. *creticus* labdane acetyltransferase (CcLAT) phylogenetic analysis. Columns list: name of characterized enzymes, Genbank ID, length (amino acids), corresponding plant species, and references. CcLAT, functionally characterized in this study, is highlighted in purple font color.

No	BAHD acetyltransferase		Genbank ID	Length	Plant species	Reference
**Clade I (D'Auria, 2006)**						
1	Dv3MAT	malonyl-coenzyme A: anthocyanin 3-O-glucoside-6''-O-malonyltransferase	Q8GSN8.1	460	*Dahlia variabilis*	Suzuki et al., 2002
2	Dm3MAT1	anthocyanidin 3-O-glucoside-6''-O-malonyltransferase	AAQ63615.1	459	*Chrysanthemum* x *morifolium*	Suzuki et al., 2004b
3	Dm3MAT2	anthocyanidin 3-O-glucoside-3'',6''-O-dimalonyltransferase	AAQ63616.1	460	*Chrysanthemum* x *morifolium*	D'Auria et al., 2002
4	Gt5AT	anthocyanin 5-aromatic acyltransferase	BAA74428.1	469	*Gentiana triflora*	Fujiwara et al., 1998
5	Lp3MAT1	quercetin 3-O-glucoside-6''-O-malonyltransferase	AAS77404.1	461	*Lamium purpureum*	Suzuki et al., 2004a
6	NtMAT1	malonyltransferase	BAD93691.1	453	*Nicotiana tabacum*	Taguchi et al., 2005
7	Pf3AT	anthocyanin acyltransferase	BAA93475.1	446	*Perilla frutescens*	Yonekura-Sakakibara et al., 2000
8	Pf5MaT	malonyl CoA:anthocyanin 5-O-glucoside-6'''-O-malonyltransferase	AAL50565.1	447	*Perilla frutescens*	Suzuki et al., 2001
9	Sc3MaT	malonyl-coenzyme A: anthocyanidin 3-O-glucoside-6''-O-malonyltransferase	AAO38058.1	461	*Pericallis cruenta*	Suzuki et al., 2003
10	Ss5MaT1	malonyl CoA:anthocyanin 5-O-glucoside-6'''-O-malonyltransferase	AAL50566.1	462	*Salvia splendens*	Suzuki et al., 2001
11	Vh3MAT1	quercetin 3-O-glucoside-6''-O-malonyltransferase	AAS77402.1	461	*Verbera* x *hybrida*	Suzuki et al., 2004a
**Clade II (D'Auria, 2006)**						
1	AtCER2	CER2	AAM64817.1	421	*Arabidopsis thaliana*	Negruk et al., 1996
2	ZmGlossy2	unknown	CAA61258.1	426	*Zea mays*	Tacke et al., 1995
**Clade III (D'Auria, 2006)**						
1	CaPun1	acyltransferase	AAV66311.1	440	*Capsicum annuum*	Stewart et al., 2005
2	CbBEAT	benzylalcohol O-acetyltransferase	AAC18062.1	433	*Clarkia breweri*	Dudareva et al., 1998
**3**	**CcLAT**	**labdane acetyltransferase**	**MT666224**	**448**	***Cistus creticus* subsp. *creticus***	**This study**
4	CmAAT4	putative alcohol acyl-transferases	AAW51126.1	479	*Cucumis melo*	El-Sharkawy et al., 2005
5	CrDAT	deacetylvindoline O-acetyltransferase	Q9ZTK5.1	439	*Catharanthus roseus*	St-Pierre et al., 1998
6	CrMAT	minovincinine 19-hydroxy-O-acetyltransferase	AAO13736.1	443	*Catharanthus roseus*	Laflamme et al., 2001
7	FaSAAT	alcohol acyltransferase	AAG13130.1	452	*Fragaria* x *ananassa*	Aharoni et al., 2000
8	FvVAAT	unnamed protein product	CAC09062.1	455	*Fragaria vesca*	Beekwilder et al., 2004
9	PsSalAT	salutaridinol 7-O-acetyltransferase	AAK73661.1	474	*Papaver somniferum*	Grothe et al., 2001

**Table S5** (continued…)						
**No**	**BAHD acetyltransferase**		**Genbank ID**	**Length**	**Plant species**	**Reference**
10	RhAAT1	acetyl CoA geraniol/citronellol acetyltransferase	AAW31948.1	457	*Rosa* x *hybrida*	Shalit et al., 2003
11	RsVS	vinorine synthase	CAD89104.2	421	*Rauvolfia serpentina*	Bayer et al., 2004
12	Ss5MaT2	pelargonidin 3-O-(6-caffeoylglucoside) 5-O-(6-O-malonylglucoside) 4'''-malonyltransferase	Q6TXD2.1	417	*Salvia splendens*	Suzuki et al., 2004c
**Clade IV (D'Auria, 2006)**						
1	HvACT	agmatine coumaroyltransferase	AAO73071.1	439	*Hordeum vulgare*	Burhenne et al., 2003
**Clade V (D'Auria, 2006)**						
1	AsHHT1	hydroxyanthranilate hydroxycinnamoyltransferase 1	BAC78633.1	441	*Avena sativa*	Yang et al., 2004
2	AtCHAT	acetyl coenzyme A: cis-3-hexen-1-ol acetyl transferase	AAN09797.1	454	*Arabidopsis thaliana*	D'Auria et al., 2002
3	AtHCT	hydroxycinnamoyl-CoA shikimate/quinate hydroxycinnamoyl transferase	NP_199704.1	433	*Arabidopsis thaliana*	Hoffmann et al., 2005
4	CbBEBT	benzoyl coenzyme A: benzyl alcohol benzoyl transferase	AAN09796.1	456	*Clarkia breweri*	D'Auria et al.,2002
5	CmAAT1	unknown	CAA94432.1	455	*Cucumis melo*	El-Sharkawy et al., 2005
6	CmAAT3	putative alcohol acyl-transferases	AAW51125.1	459	*Cucumis melo*	El-Sharkawy et al., 2005
7	DcHCBT	anthranilate N-hydroxycinnamoyl/benzoyltransferase	CAB06430.1	446	*Dianthus caryophyllus*	Yang et al., 1997
8	LaHMT/HLT	(-)-13alpha-hydroxymultiflorine/ (+)-13alpha- hydroxylupanine O-tigloyltransferase	BAD89275.1	453	*Lupinus albus*	Okada et al., 2005
9	MpAAT1	alcohol acyl transferase	AAU14879.2	455	*Malus domestica*	Souleyre et al., 2005
10	MsAAT	unnamed protein product	CAC09063.1	419	*Musa sapientum*	Beekwilder et al., 2004
11	NtBEBT	benzoyl coenzyme A: benzyl alcohol benzoyl transferase	AAN09798.1	460	*Nicotiana tabacum*	D'Auria et al.,2002
12	NtHCT	hydroxycinnamoyl transferase	CAD47830.1	435	*Nicotiana tabacum*	Hoffmann et al., 2003
13	NtHQT	hydroxycinnamoyl CoA quinate transferase	CAE46932.1	436	*Nicotiana tabacum*	Niggeweg et al.,2004
14	PhBPBT	benzoyl-CoA:benzyl alcohol/phenylethanol benzoyltransferase	AAU06226.1	460	*Petunia* x *hybrida*	Boatright et al., 2004
15	TcBAPT	phenylpropanoyltransferase	AAL92459.1	445	*Taxus cuspidata*	Walker et al., 2002a
16	TcDBAT	10-deacetylbaccatin III-10-O-acetyl transferase	AAF27621.1	440	*Taxus cuspidata*	Walker and Croteau, 2000
17	TcDBBT	2-alpha-hydroxytaxane 2-O-benzoyltransferase	Q9FPW3.1	440	*Taxus cuspidata*	Walker and Croteau, 2000
18	TcDBNTBT	3'-N-debenzoyltaxol N-benzoyltransferase	AAM75818.1	441	*Taxus canadensis*	Walker et al., 2002b
19	TcTAT	taxadienol acetyl transferase	AAF34254.1	439	*Taxus cuspidata*	Walker et al., 2000
20	VlAMAT	anthraniloyal-CoA: methanol anthraniloyal transferase	AAW22989.1	449	*Vitis labrusca*	Wang and De Luca, 2005

Table S6. List of primers used for gene cloning in this study (manually designed).		
**Gene**	**Sequence (5’-3’)**	**ORF amplicon size (bp)**
CcLDDS1	CcLDDS1.FORWARD: ATGCTTCCCTCTACTTCTTCTTCC	1-2427
	CcLDDS1.FORWARD(*Kpn*I): GAGTGGTACCATGCTTCCCTCTACTTCTTCTTCC	
	CcLDDS1.REVERSE: TCAATCAACTCTTTCAAAAAGCAC	
	CcLDDS1.REVERSE(*Xba*I): ACTCTCTAGAAATCAATCAACTCTTTCAAAAAGCAC	
***CcLDDS1*(Δ40)**	CcLDDS1(Δ40).FORWARD(*Bam*HI): TACTCAGGATCCATGGGTAGAGCTAAAGACAAAACACGG	121-2427
	CcLDDS1.REVERSE(*Not*I): TACTCAGCGGCCGCCAAATCAATCAACTCTTTCAAAAAGCAC	
***CcLDDS2*(Δ42)**	CcLDDS2(Δ42).FORWARD(*Bam*HI): GCATGAGGATCCAAGTTTGATACCCGGCTCAGATGC	127-2490
	CcLDDS2.REVERSE(*Sph*I): GCATGAGCATGCTCAAACAACTGATTCAAAAAGTACTT	
CcLAT	CcLAT.FORWARD(*Bam*HI): GCATGAGGATCCATGAAATTAGGAAAAAATAGAATG	1-1347
	CcLAT.REVERSE(*Kpn*I): GCATGAGGTACCTCAATCCAATAACTTGATGG	
	CcLAT.REVERSE(*Not*I): GCATGAGCGGCCGCTTCAATCCAATAACTTGATGGGA	
SmCPSKSL1	SmCPSKSL1.FORWARD(*Not*I): GCATGAGCGGCCGCTCATGATAGAGGAAATGAGAAAATTGCTTGC	1-2253
	SmCPSKSL1.REVERSE(*Xho*I): GCATGACTCGAGTCATTCAGCTGCTTTATACAACACATT	

Table S7. NMR analysis of labda-7,13(*E*)-dien-15-ol.				
Pos.	^{1}H (nH; m; J(Hz))	^{13}C ($CDCl_3$)	COSY	HMBC
1	0.95(1H, dt, 13Hz,3.6Hz, H1a), 1.84 (1H, m, H1b)	39.1	H2a, H2b, H3a, H3b, $H_3$20	
2	1.43 (1H, m, H2a), 1.53 (1H, m, H2b)	18.8	H3a, H3b, H1a, H1b	C-1
3	1.15 (1H, d, 13.4Hz, H3a),1.39 (1H, t, H3b)	42.3	H2a, H1b	
4		32.9		
5	1.17 (1H, m, H5)	50.1	H6a, H6b, $H_3$18, $H_3$19	C-4, C-6, C-10, C-20, C-9
6	1.84 (1H, m, H6a), 1.94 (1H, m, H6b)	24.8		C-7, C-8
7	5.39 (1H, brs, H7)	122.3	H6a, H6b	
8		135.2		
9	1.60 (1H, s, H9)	54.4		C-11, C-17
10		36.8		
11	1.26 (1H, m, H11a),1.53(1H, m, H11b)	25.6	H12a, H12b	C-8, C-9, C-10, C-12
12	1.95 (1H, m, H12a), 2.23 (1H, dt, 4.7,12.4 Hz, H12b)	42.1	H11a, H11b	C-11, C-13, C-14
13		140.4		
14	5.42 (1H, t,6.8 Hz, H14)	123.2	H15a, H15b	C-12, C-16
15	4.15 (2H, d, 6.9 Hz, H15a-b)	59.4	H14	C-13, C-14
16	1.69 (3H, s, H16)	16.4		C-12, C-13, C-16
17	1.69 (3H, s, H17)	22.2		C-7, C-8, C-9
18	0.85 (3H, s, H18)	33.2		C-3, C-4, C-5, C-19
19	0.87 (3H, s, H19)	21.8		C-3, C-4, C-5, C-18
20	0.75 (3H, s, H20)	13.6		C-1, C-5, C-9, C-10

Table S8. List of primers used for qRT-PCR analysis (manually designed).		
**Gene**	**Sequence (5'-3')**	**Amplicon size (bp)**
CcActin	*CcActin* (371-395F): TTATGTTCGAGACCTTCAACACTCC	109
	CcActin (480-455R): ACCATCTCCAGAATCCAACACAATAC	
CcELFα	*CcELFα* (35-59F): AGCCTGGTATGGTTGTCACTTTTGG	105
	CcELFα (140-117R): ACATTGTCACCAGGGAGAGCTTCG	
CcCLS	*CcCLS* (1894-1919F): AACATCTACGAAAGAAGAATGGAAGC	97
	CcCLS (1991-1969R): CTACCACAGACCTCCGTCAAGTG	
CcLDDS1	*CcLDDS1* (2219-2243F): TGGCTGGTTTTTTGGACGGTAATGT	98
	CcLDDS1 (2317-2295R): GCCGCTTATCACCATCATCCGAC	
CcLDDS2	*CcLDDS2* (1846-1870F): GTTATTGGCTCTCTCTTTCACGATG	113
	CcLDDS2 (1959-1936R): TCTTCCATGTATGTGGCCTTCTCC	
CcLAT	*CcLAT* (757-779F): AAGAAGCCGAGCAGAGTGGAAGC	112
	CcLAT (869-844R): TTCACTACTTGGACTATTACTGATGG	

Table S9. Summary of Contig and Unigene statistics of RNA-seq data analysis from *C. creticus* subsp. *creticus* trichomes isolated from leaves of developmental stage 2.

a. RNA-seq output: total raw reads, total clean reads, total clean nucleotides (nt), Q stands for Phred quality score (Q20 = % bases of Q20) and N percentage for the occurrence of N (any nucleotide) and GC percentage.

**Total Raw Reads**	**Total Clean Reads**	**Total Clean Nucleotides (nt)**	**Q20 percentage**	**N percentage**	**GC percentage**
65,177,170	53,440,826	4,809,674,340	97.14%	0.00%	48.67%

b. Assembly quality statistics. Contig or unigene N50 is a weighted median statistic such that 50% of the entire assembly is contained in contigs or unigenes equal to or larger than this value. Total number, total length (nt), mean length, N50 value (shortest Contig/Unigene sequence length that 50% of the total assembled nucleotides are contained in), total consensus, district clusters, distinct singleton (unigenes).

	**Total Number**	**Total Length (nt)**	**Mean Length (nt)**	**N50**	**Total Consensus Sequences**	**Distinct Clusters**	**Distinct Singletons (unigenes)**
**Contig**	385,143	79,670,010	207	241	-	-	-
**Unigene**	114,239	50,255,324	440	477	114,239	25,470	88,769

Table S10. CcCLS (Falara et al., 2010), CcLDDS1, and CcLDDS2 protein sequence similarity (%) (BLAST-p, NCBI).			
	**CcCLS** (class II, 808 amino acids)	**CcLDDS1** (class II, 808 amino acids)	**CcLDDS2** (class II, 829 amino acids)
**CcCLS** (class II, 808 amino acids)	**100**	72	75
**CcLDDS1** (class II, 808 amino acids)	**72**	**100**	60
**CcLDDS2** (class II, 829 amino acids)	**75**	**60**	**100**

Additional List of References for Supplementary Data

(*References not included in the main manuscript*)

Andersen-Ranberg J, Kongstad KT, Nielsen MT, *et al*. 2016. Expanding the landscape of diterpene structural diversity through stereochemically controlled combinatorial biosynthesis. Angewandte Chemie International Edition **55**, 2142-2146.

Bayer A, Ma X, Stöckigt J. 2004. Acetyltransfer in natural product biosynthesis-functional cloning and molecular analysis of vinorine synthase. Bioorganic & Medicinal Chemistry **12**, 2787-2795.

Bensen RJ, Johal GS, Crane VC, Tossberg JT, Schnable PS, Meeley RB, Briggs SP. 1995. Cloning and characterization of the maize *An1* gene. The Plant Cell **7**, 75-84.

Boatright J, Negre F, Chen X, Kish CM, Wood B, Peel G, Orlova I, Gang D, Rhodes D, Dudareva N. 2004. Understanding *in vivo* benzenoid metabolism in petunia petal tissue. Plant Physiology **135**, 1993-2011.

Burhenne K, Kristensen BK, Rasmussen SK. 2003. A new class of *N*-hydroxycinnamoyltransferases: purification, cloning, and expression of a barley agmatine coumaroyltransferase (EC 2.3.1.64). Journal of Biological Chemistry **278**, 13919-13927.

Chen X, Berim A, Dayan FE, Gang DR. 2017. A (–)-kolavenyl diphosphate synthase catalyzes the first step of salvinorin A biosynthesis in *Salvia divinorum*. Journal of Experimental Botany **68**, 1109-1122.

Cui G, Duan L, Jin B, Qian J, Xue Z, Shen G, Snyder JH, Song J, Chen S, Huang L, Peters RJ, Qi X. 2015. Functional divergence of diterpene syntheses in the medicinal plant *Salvia miltiorrhiza*. Plant Physiology **169**, 1607-1618.

D'Auria JC, Chen F, Pichersky E. 2002. Characterization of an acyltransferase capable of synthesizing benzylbenzoate and other volatile esters in flowers and damaged leaves of *Clarkia breweri*. Plant Physiology **130**, 466-476.

Dudareva N, D'Auria JC, Nam KH, Raguso RA, Pichersky E. 1998. Acetyl-CoA: benzylalcohol acetyltransferase– an enzyme involved in floral scent production in *Clarkia breweri*. The Plant Journal **14**, 297-304.

El-Sharkawy I, Manríquez D, Flores FB, Regad F, Bouzayen M, Latche A, Pech JC. 2005. Functional characterization of a melon alcohol acyl-transferase gene family involved in the biosynthesis of ester volatiles. Identification of the crucial role of a threonine residue for enzyme activity. Plant Molecular Biology **59**, 345-362.

Falara V, Akhtar TA, Nguyen TTH, *et al*. 2011. The tomato terpene synthase gene family. Plant Physiology **157**, 770-789.

Gao W, Hillwig ML, Huang L, Cui G, Wang X, Kong J, Yang B, Peters RJ. 2009. A functional genomics approach to tanshinone biosynthesis provides stereochemical insights. Organic Letters **11**, 5170-5173.

Grothe T, Lenz R, Kutchan TM. 2001. Molecular characterization of the salutaridinol 7-*O*-acetyltransferase involved in morphine biosynthesis in opium poppy *Papaver somniferum*. Journal of Biological Chemistry **276**, 30717-30723.

Hansen NL, Heskes AM, Hamberger B, Olsen CE, Hallström BM, Andersen-Ranberg J, Hamberger B. 2017. The terpene synthase gene family in *Tripterygium wilfordii* harbors a labdane-type diterpene synthase among the monoterpene synthase TPS-b subfamily. The Plant Journal **89**, 429-441.

Harris LJ, Saparno A, Johnston A, Prisic S, Xu M, Allard S, Kathiresan A, Ouellet T, Peters RJ. 2005. The maize *An2* gene is induced by *Fusarium* attack and encodes an *ent*-copalyl diphosphate synthase. Plant Molecular Biology **59**, 881-894.

Hoffmann L, Besseau S, Geoffroy P, Ritzenthaler C, Meyer D, Lapierre C, Pollet B, Legrand M. 2005. Acyltransferase-catalysed *p*-coumarate ester formation is a committed step of lignin biosynthesis. Plant Biosystems-An International Journal Dealing with all Aspects of Plant Biology **139**, 50-53.

Hoffmann L, Maury S, Martz F, Geoffroy P, Legrand M. 2003. Purification, cloning, and properties of an acyltransferase controlling shikimate and quinate ester intermediates in phenylpropanoid metabolism. Journal of Biological Chemistry **278**, 95-103.

Ignea C, Ioannou E, Georgantea P, Loupassaki S, Trikka FA, Kanellis AK, Makris AM, Roussis V, Kampranis SC. 2015b. Reconstructing the chemical diversity of labdane-type diterpene biosynthesis in yeast. Metabolic Engineering **28**, 91-103.

Inabuy FS, Fischedick JT, Lange I, Hartmann M, Srividya N, Parrish AN, Xu M, Peters RJ, Lange BM. 2017. Biosynthesis of diterpenoids in *Tripterygium* adventitious root cultures. Plant Physiology **175**, 92-103.

Irmisch S, Müller AT, Schmidt L, Günther J, Gershenzon J, Köllner TG. 2015. One amino acid makes the difference: the formation of *ent*-kaurene and 16α-hydroxy-*ent*-kaurane by diterpene synthases in poplar. BMC Plant Biology **15**, 1-13.

Jin B, Cui G, Guo J, Tang J, Duan L, Lin H, Shen Y, Chen T, Zhang H, Huang L. 2017. Functional diversification of kaurene synthase-like genes in *Isodon rubescens*. Plant Physiology **174**, 943-955.

Keeling CI, Dullat HK, Yuen M, Ralph SG, Jancsik S, Bohlmann J. 2010. Identification and functional characterization of monofunctional *ent*-copalyl diphosphate and *ent*-kaurene synthases in white spruce reveal different patterns for diterpene synthase evolution for primary and secondary metabolism in gymnosperms. Plant Physiology **152**, 1197-1208.

Laflamme P, St-Pierre B, De Luca V. 2001. Molecular and biochemical analysis of a Madagascar periwinkle root-specific minovincinine-19-hydroxy-*O*-acetyltransferase. Plant Physiology **125**, 189-198.

Li J-L, Chen Q-Q, Jin QP, Gao J, Zhao PJ, Lu S, Zeng Y. 2012. *IeCPS2* is potentially involved in the biosynthesis of pharmacologically active *Isodon* diterpenoids rather than gibberellin. Phytochemistry **76**, 32-39.

Margis-Pinheiro M, Zhou XR, Zhu QH, Dennis ES, Upadhyaya NM. 2005. Isolation and characterization of a *Ds*-tagged rice (*Oryza sativa* L.) GA-responsive dwarf mutant defective in an early step of the gibberellin biosynthesis pathway. Plant Cell Reports **23**, 819-833.

Martin DM, Fäldt J, Bohlmann J. 2004. Functional characterization of nine Norway spruce *TPS* genes and evolution of gymnosperm terpene synthases of the *TPS-d* subfamily. Plant Physiology **135**, 1908-1927.

Murphy KM, Ma LT, Ding Y, Schmelz EA, Zerbe P. 2018. Functional characterization of two class II diterpene synthases indicates additional specialized diterpenoid pathways in maize (*Zea mays*). Frontiers in Plant Science **9**, 1542.

Negruk V, Yang P, Subramanian M, McNevin JP, Lemieux B. 1996. Molecular cloning and characterization of the *CER2* gene of *Arabidopsis thaliana*. The Plant Journal **9**, 137-145.

Nemoto T, Cho EM, Okada A, *et al*. 2004. Stemar-13-ene synthase, a diterpene cyclase involved in the biosynthesis of the phytoalexin oryzalexin S in rice. FEBS Letters **571**, 182-186.

Niggeweg R, Michael AJ, Martin C. 2004. Engineering plants with increased levels of the antioxidant chlorogenic acid. Nature Biotechnology **22**, 746-754.

Okada T, Hirai MY, Suzuki H, Yamazaki M, Saito K. 2005. Molecular characterization of a novel quinolizidine alkaloid O-tigloyltransferase: cDNA cloning, catalytic activity of recombinant protein and expression analysis in *Lupinus* plants. Plant and Cell Physiology **46**, 233-244.

Pelot KA, Chen R, Hagelthorn DM, Young CA, Addison JB, Muchlinski A, Tholl D, Zerbe P. 2018. Functional diversity of diterpene synthases in the biofuel crop switchgrass. Plant Physiology **178**, 54-71.

Pelot KA, Hagelthorn LM, Addison JB, Zerbe P. 2017a. Biosynthesis of the oxygenated diterpene nezukol in the medicinal plant *Isodon rubescens* is catalyzed by a pair of diterpene synthases. PLoS One **12**, e0176507.

Prisic S, Xu M, Wilderman PR, Peters RJ. 2004. Rice contains two disparate *ent*-copalyl diphosphate synthases with distinct metabolic functions. Plant Physiology **136**, 4228-4236.

Pugliesi C, Fambrini M, Salvini M. 2011. Molecular cloning and expression profile analysis of three sunflower (*Helianthus annuus*) diterpene synthase genes. Biochemical Genetics **49**, 46-62.

Richman AS, Gijzen M, Starratt AN, Yang Z, Brandle JE. 1999. Diterpene synthesis in *Stevia rebaudiana*: recruitment and up-regulation of key enzymes from the gibberellin biosynthetic pathway. The Plant Journal **19**, 411-421.

Sallaud C, Giacalone C, Töpfer R, Goepfert S, Bakaher N, Rösti S, Tissier A. 2012. Characterization of two genes for the biosynthesis of the labdane diterpene *Z*-abienol in tobacco (*Nicotiana tabacum*) glandular trichomes. The Plant Journal **72**, 1-17.

Sawada Y, Katsumata T, Kitamura J, Kawaide H, Nakajima M, Asami T, Nakaminami K, Kurahashi T, Mitsuhashi W, Inoue Y, Toyomasu T. 2008. Germination of photoblastic lettuce seeds is regulated via the control of

endogenous physiologically active gibberellin content, rather than of gibberellin responsiveness. Journal of Experimental Botany **59**, 3383-3393.

Schepmann HG, Pang J, Matsuda SPT. 2001. Cloning and characterization of *Ginkgo biloba* levopimaradiene synthase, which catalyzes the first committed step in ginkgolide biosynthesis. Archives of Biochemistry and Biophysics **392**, 263-269.

Smith MW, Yamaguchi S, Ait-Ali T, Kamiya Y. 1998. The first step of gibberellin biosynthesis in pumpkin is catalyzed by at least two copalyl diphosphate synthases encoded by differentially regulated genes. Plant Physiology **118**, 1411-1419.

Souleyre EJF, Greenwood DR, Friel EN, Karunairetnam S, Newcomb RD. 2005. An alcohol acyl transferase from apple (cv. Royal Gala), MpAAT1, produces esters involved in apple fruit flavor. The FEBS Journal **272**, 3132-3144.

Spielmeyer W, Ellis M, Robertson M, Ali S, Lenton JR, Chandler PM. 2004. Isolation of gibberellin metabolic pathway genes from barley and comparative mapping in barley, wheat and rice. Theoretical and Applied Genetics **109**, 847-855.

Stewart CJr, Kang BC, Liu K, Mazourek M, Moore SL, Yoo EY, Kim BD, Paran I, Jahn MM. 2005. The *Pun1* gene for pungency in pepper encodes a putative acyltransferase. The Plant Journal **42**, 675-688.

Stoffer-Vogel B, Wildung MR, Vogel G, Croteau R. 1996. Abietadiene synthase from grand fir (*Abies grandis*) cDNA isolation, characterization, and bacterial expression of a bifunctional diterpene cyclase involved in resin acid biosynthesis. Journal of Biological Chemistry **271**, 23262-23268.

St-Pierre B, Laflamme P, Alarco AM, De Luca V. 1998. The terminal *O*-acetyltransferase involved in vindoline biosynthesis defines a new class of proteins responsible for coenzyme A-dependent acyl transfer. The Plant Journal **14**, 703-713.

Sugai Y, Ueno Y, Hayashi KI, Oogami S, Toyomasu T, Matsumoto S, Natsume M, Nozaki H, Kawaide H. 2011. Enzymatic ^{13}C labeling and multidimensional NMR analysis of miltiradiene synthesized by bifunctional diterpene cyclase in *Selaginella moellendorffii*. Journal of Biological Chemistry **286**, 42840-42847.

Sun TP, Kamiya Y. 1994. The Arabidopsis *GA1* locus encodes the cyclase *ent*-kaurene synthetase A of gibberellin biosynthesis. The Plant Cell **6**, 1509-1518.

Suzuki H, Nakayama T, Nagae S, Yamaguchi MA, Iwashita T, Fukui Y, Nishino T. 2004a. cDNA cloning and functional characterization of flavonol 3-*O*-glucoside-6″-*O*-malonyltransferases from flowers of *Verbena hybrida* and *Lamium purpureum*. Journal of Molecular Catalysis B: Enzymatic **28**, 87-93.

Suzuki H, Nakayama T, Yamaguchi MA, Nishino T. 2004b. cDNA cloning and characterization of two *Dendranthema* × *morifolium* anthocyanin malonyltransferases with different functional activities. Plant Science **166**, 89-96.

Suzuki H, Nakayama T, Yonekura-Sakakibara K, Fukui Y, Nakamura N, Yamaguchi MA, Tanaka Y, Kusumi T, Nishino, T. 2002. cDNA cloning, heterologous expressions, and functional characterization of malonyl-coenzyme A: anthocyanidin 3-*O*-glucoside-6"-*O*-malonyltransferase from *Dahlia* flowers. Plant Physiology **130**, 2142-2151.

Suzuki H, Nakayama T, Yonekura-Sakakibara K, Fukui Y, Nakamura N, Nakao M, Tanaka Y, Yamaguchi MA, Kusumi T, Nishino T. 2001. Malonyl-CoA: anthocyanin 5-*O*-glucoside-6‴-*O*-malonyltransferase from scarlet sage (*Salvia splendens*) flowers. Enzyme purification, gene cloning, expression, and characterization. Journal of Biological Chemistry **276**, 49013-49019.

Suzuki H, Sawada S, Yonekura-Sakakibara K, Nakayama T, Yamaguchi M, Nishino T. 2003. Identification of a cDNA encoding malonyl-Coenzyme A: anthocyanidin 3-*O*-glucoside 6”-*O*-malonyltransferase from Cineraria (*Senecio cruentus*) flowers. Plant Biotechnology **20**, 229-234.

Suzuki H, Sawada SY, Watanabe K, Nagae S, Yamaguchi MA, Nakayama T, Nishino T. 2004c. Identification and characterization of a novel anthocyanin malonyltransferase from scarlet sage (*Salvia splendens*) flowers: an enzyme that is phylogenetically separated from other anthocyanin acyltransferases. The Plant Journal **38,** 994-1003.

Tacke E, Korfhage C, Michel D, Maddaloni M, Motto M, Lanzini S, Salamini F, Döring HP. 1995. Transposon tagging of the maize *Glossy2* locus with the transposable element *En/Spm*. The Plant Journal **8**, 907-917.

Taguchi G, Shitchi Y, Shirasawa S, Yamamoto H, Hayashida N. 2005. Molecular cloning, characterization, and downregulation of an acyltransferase that catalyzes the malonylation of flavonoid and naphthol glucosides in tobacco cells. The Plant Journal **42**, 481-491.

Toyomasu T, Kagahara T, Hirose Y, Usui M, Abe S, Okada K, Koga J, Mitsuhashi W, Yamane H. 2009. Cloning and characterization of cDNAs encoding *ent*-copalyl diphosphate synthases in wheat: insight into the evolution of rice phytoalexin biosynthetic genes. Bioscience, Biotechnology, and Biochemistry **73**, 772-775.

Walker K, Croteau R. 2000. Taxol biosynthesis: molecular cloning of a benzoyl-CoA: taxane 2α-*O*-benzoyltransferase cDNA from *Taxus* and functional expression in *Escherichia coli*. Proceedings of the National Academy of Sciences **97**, 13591-13596.

Walker K, Fujisaki S, Long R, Croteau R. 2002a. Molecular cloning and heterologous expression of the C-13 phenylpropanoid side chain-CoA acyltransferase that functions in Taxol biosynthesis. Proceedings of the National Academy of Sciences **99**, 12715-12720.

Walker K, Long R, Croteau R. 2002b. The final acylation step in taxol biosynthesis: cloning of the taxoid C13-side-chain *N*-benzoyltransferase from *Taxus*. Proceedings of the National Academy of Sciences **99**, 9166-9171.

Walker K, Schoendorf A, Croteau R. 2000. Molecular cloning of a taxa-4(20),11(12)-dien-5α-ol-*O*-acetyl transferase cDNA from *Taxus* and functional expression in *Escherichia coli*. Archives of Biochemistry and Biophysics **374**, 371-380.

Wang J, De Luca V. 2005. The biosynthesis and regulation of biosynthesis of Concord grape fruit esters, including 'foxy' methylanthranilate. The Plant Journal **44**, 606-619.

Wilderman PR, Xu M, Jin Y, Coates RM, Peters RJ. 2004. Identification of *syn*-pimara-7,15-diene synthase reveals functional clustering of terpene synthases involved in rice phytoalexin/allelochemical biosynthesis. Plant Physiology **135**, 2098-2105.

Wildung MR, Croteau R. 1996. A cDNA clone for taxadiene synthase, the diterpene cyclase that catalyzes the committed step of taxol biosynthesis. Journal of Biological Chemistry **271**, 9201-9204.

Wu Y, Zhou K, Toyomasu T, Sugawara C, Oku M, Abe S, Usui M, Mitsuhashi W, Chono M, Chandler PM, Peters RJ. 2012. Functional characterization of wheat copalyl diphosphate synthases sheds light on the early evolution of labdane-related diterpenoid metabolism in the cereals. Phytochemistry **84**, 40-46.

Xu M, Hillwig ML, Prisic S, Coates RM, Peters RJ. 2004. Functional identification of rice *syn*-copalyl diphosphate synthase and its role in initiating biosynthesis of diterpenoid phytoalexin/allelopathic natural products. The Plant Journal **39**, 309-318.

Xu M, Wilderman PR, Morrone D, Xu J, Roy A, Margis-Pinheiro M, Upadhyaya NM, Coates RM, Peters RJ. 2007. Functional characterization of the rice kaurene synthase-like gene family. Phytochemistry **68**, 312-326.

Yamaguchi S, Saito T, Abe H, Yamane H, Murofushi N, Kamiya Y. 1996. Molecular cloning and characterization of a cDNA encoding the gibberellin biosynthetic enzyme *ent*-kaurene synthase B from pumpkin (*Cucurbita maxima* L.). The Plant Journal **10**, 203-213.

Yamaguchi S, Sun TP, Kawaide H, Kamiya Y. 1998. The *GA2* locus of *Arabidopsis thaliana* encodes *ent*-kaurene synthase of gibberellin biosynthesis. Plant Physiology **116**, 1271-1278.

Yamamura Y, Taguchi Y, Ichitani K, Umebara I, Ohshita A, Kurosaki F, Lee JB. 2018. Characterization of *ent*-kaurene synthase and kaurene oxidase involved in gibberellin biosynthesis from *Scoparia dulcis*. Journal of Natural Medicines **72**, 456-463.

Yang R, Du Z, Qiu T, Sun J, Shen Y, Huang L. 2021. Discovery and functional characterization of a diverse diterpene synthase family in the medicinal herb *Isodon lophanthoides* var. *gerardiana*. Plant and Cell Physiology **62**, 1423-1435.

Yang Q, Reinhard K, Schiltz E, Matern U. 1997. Characterization and heterologous expression of hydroxycinnamoyl/benzoyl-CoA: anthranilate N-hydroxycinnamoyl/benzoyltransferase from elicited cell cultures of carnation, *Dianthus caryophyllus* L. Plant Molecular Biology **35**, 777-789.

Yang Q, Trinh HX, Imai S, Ishihara A, Zhang L, Nakayashiki H, Tosa Y, Mayama S. 2004. Analysis of the involvement of hydroxyanthranilate hydroxycinnamoyltransferase and caffeoyl-CoA 3-*O*-methyltransferase in phytoalexin biosynthesis in oat. Molecular Plant-Microbe Interactions **17**, 81-89.

Yonekura-Sakakibara K, Tanaka Y, Fukuchi-Mizutani M, Fujiwara H, Fukui Y, Ashikari T, Murakami Y, Yamaguchi M, Kusumi T. 2000. Molecular and biochemical characterization of a novel hydroxycinnamoyl-CoA: anthocyanin 3-*O*-glucoside-6''-*O*-acyltransferase from *Perilla frutescens*. Plant and Cell Physiology **41**, 495-502.

Zerbe P, Chiang A, Dullat H, O'Neil-Johnson M, Starks C, Hamberger B, Bohlmann J. 2014. Diterpene synthases of the biosynthetic system of medicinally active diterpenoids in *Marrubium vulgare*. The Plant Journal **79**, 914-927.

Zerbe P, Chiang A, Yuen M, Hamberger B, Hamberger B, Draper JA, Britton R, Bohlmann J. 2012. Bifunctional *cis*-abienol synthase from *Abies balsamea* discovered by transcriptome sequencing and its implications for diterpenoid fragrance production. Journal of Biological Chemistry **287**, 12121-12131.

Zerbe P, Hamberger B, Yuen MMS, Chiang A, Sandhu HK, Madilao LL, Nguyen A, Hamberger B, Bach SS, Bohlmann J. 2013. Gene discovery of modular diterpene metabolism in nonmodel systems. Plant Physiology **162**, 1073-1091.

Zhou K, Gao Y, Hoy JA, Mann FM, Honzatko RB, Peters RJ. 2012. Insights into diterpene cyclization from structure of bifunctional abietadiene synthase from *Abies grandis*. Journal of Biological Chemistry **287**, 6840-6850.
